# Identification of the lydiamycin biosynthetic gene cluster in a plant pathogen guides structural revision and identification of molecular target

**DOI:** 10.1073/pnas.2424388122

**Published:** 2025-05-19

**Authors:** Jonathan J. Ford, Javier Santos-Aberturas, Edward S. Hems, Joseph W. Sallmen, Lena A. K. Bögeholz, Guy Polturak, Anne Osbourn, Joseph A. Wright, Marina V. Rodnina, Danny Vereecke, Isolde M. Francis, Andrew W. Truman

**Affiliations:** ^a^Department of Molecular Microbiology, John Innes Centre, Norwich NR4 7UH, United Kingdom; ^b^Centre for Microbial Interactions, Norwich NR4 7UG, United Kingdom; ^c^Department of Physical Biochemistry, Max Planck Institute for Multidisciplinary Sciences, Göttingen 37077, Germany; ^d^Department of Biochemistry and Metabolism, John Innes Centre, Norwich NR4 7UH, United Kingdom; ^e^School of Chemistry, University of East Anglia, Norwich NR4 7TJ, United Kingdom; ^f^School of Nursing, Howest University of Applied Sciences, Bruges 8200, Belgium; ^g^Department of Biology, California State University, Bakersfield, CA 93311

**Keywords:** biosynthesis, nonribosomal peptides, antibiotics, peptide deformylase, *Rhodococcus*

## Abstract

The natural products actinonin and matlystatin feature an *N*-hydroxy-2-pentyl-succinamyl (HPS) chemophore that facilitates metal chelation and confers their metalloproteinase inhibitory activity. Actinonin is the most potent natural inhibitor of peptide deformylase (PDF) and exerts antimicrobial and herbicidal bioactivity by disrupting protein synthesis. Here, we used a genomics-led approach to identify candidate biosynthetic gene clusters (BGCs) hypothesized to produce HPS-containing natural products. We show that one of these BGCs is on the pathogenicity megaplasmid of the plant pathogen *Rhodococcus fascians* and produces lydiamycin A, a macrocyclic pentapeptide. The presence of genes predicted to make an HPS-like chemophore informed the structural recharacterization of lydiamycin via NMR and crystallography to show that it features a rare 2-pentyl-succinyl chemophore. We demonstrate that lydiamycin A inhibits bacterial PDF in vitro and show that a cluster-situated PDF gene confers resistance to lydiamycin A, representing an uncommon self-immunity mechanism associated with the production of a PDF inhibitor*. In planta* competition assays showed that lydiamycin enhances the fitness of *R. fascians* during plant colonization. This study highlights how a BGC can inform the structure, biochemical target, and ecological function of a natural product.

## Significance

There is an urgent requirement to discover new antibiotics. One approach is to use natural products, which are small bioactive molecules that provide producing organisms with beneficial biological traits. Genomic data can be used to rationally identify biosynthetic pathways predicted to make antibiotics. Here, we use genomic data to find that a plant pathogen, *Rhodococcus fascians*, makes the known antibiotic lydiamycin. Based on our prediction of its biosynthesis, we propose and prove a revised structure for lydiamycin. Genetic data also guided the identification of its molecular target, as well as an ecological role for lydiamycin during plant colonization. Collectively, these data highlight the power of using biosynthetic gene cluster data to identify the structure and function of antibiotics.

**Fig. 1.**
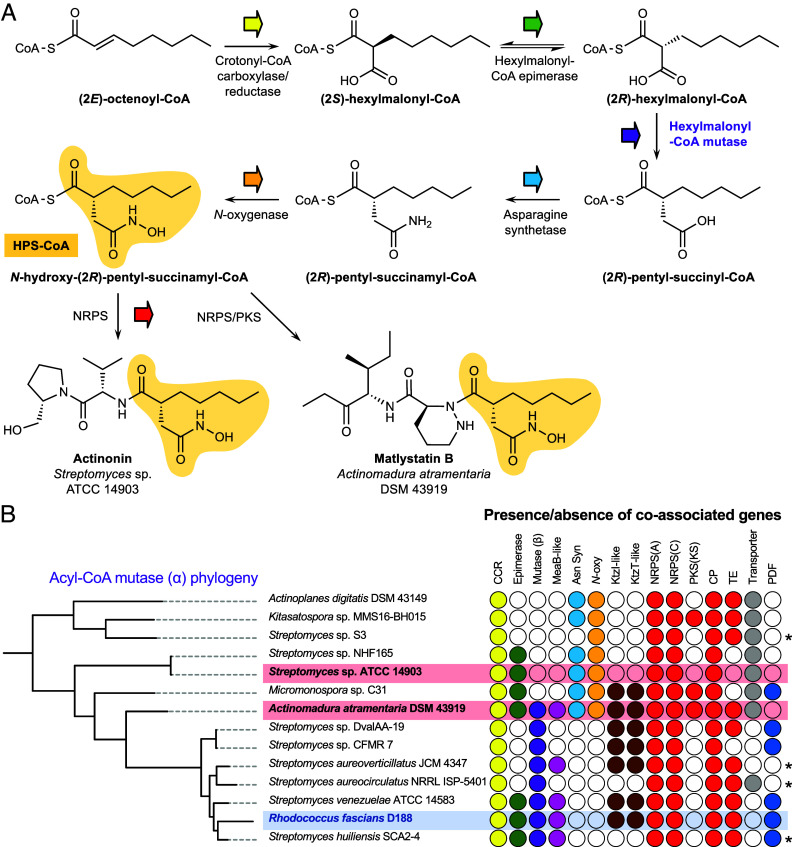
Mutase-guided BGC discovery. (*A*) Proposed HPS biosynthesis for actinonin and matlystatin. (*B*) Clade of actinobacterial mutases that are highly coassociated with the proposed HPS biosynthesis genes (color-coded as in panel *A*), NRPS/PKS genes (red), and PDF gene (green). A filled circle indicates the presence of a homologous gene in proximity to the mutase gene. The actinonin and matlystatin producers are indicated in red. Asterisks indicate where BGCs are at the end of a contig and may therefore be incomplete. Abbreviations: CCR = crotonyl-CoA carboxylase-reductase, Asn Syn = asparagine synthetase, *N*-oxy = *N*-oxygenase, NRPS(A) = NRPS adenylation domain, NRPS(C) = NRPS condensation domain, PKS(KS) = PKS ketosynthase domain, CP = acyl or peptidyl carrier protein, TE = thioesterase.

Natural products (NPs) provide producing organisms with beneficial traits, such as intraspecies communication, nutrient acquisition, or growth inhibition of competing organisms (1). NPs are also critical in medicine and agriculture, where they have diverse uses, including as immunosuppressive, antiparasitic, antifungal, and anticancer medicines (2), as well as accounting for the majority of approved antibiotics (3). This success can be attributed to the evolutionarily beneficial selection of potent chemotypes that strongly bind relevant biological targets. Actinonin and matlystatin are structurally related actinobacterial NPs that feature the same *N*-hydroxy-(*R*)-2-pentyl-succinamyl (HPS) chemophore (4, 5) (Fig. 1*A*). This chemophore chelates metal ions in the active sites of metalloproteins via a hydroxamate group (6). This activity means that actinonin exhibits nanomolar activity toward multiple targets that have been validated in disease models in mice, including as an antitumor candidate (7), acute renal failure (8), antimalarial (9), antiobesity (10), and antibacterial, where the HPS moiety is a structural mimic of *N*-formylmethionine (11), the substrate of the metalloproteinase peptide deformylase (PDF). PDF catalyzes the deformylation of the initiator methionine residue on the growing nascent peptide, which is necessary for protein maturation, and is essential for the viability of most bacteria. Synthetic metalloprotease inhibitors inspired by actinonin have also been investigated in clinical trials as antibiotics, including LBM415 and GSK1322322 (12).

We previously identified BGCs for actinonin and matlystatin (13), which showed that these NPs are biosynthesized by either a nonribosomal peptide synthetase [NRPS; actinonin (14)] or a hybrid NRPS/polyketide synthase (NRPS/PKS; matlystatin), where it was proposed that the HPS moiety was biosynthesized as a coenzyme A (CoA)-bound precursor and condensed at the beginning of the assembly line. A combination of bioinformatic analysis and stable isotope feeding led to a proposal for the biosynthesis of the HPS group (Fig. 1*A*), although the precise timing of biosynthetic steps remains to be determined. A key step in the proposed pathway is a mutase-catalyzed rearrangement to generate the characteristic HPS carbon skeleton. This proposed mutase specificity toward hexylmalonyl-CoA would be specific to HPS biosynthesis, so we hypothesized that it could serve as an effective genetic marker for finding novel HPS-containing NPs.

*Rhodococcus fascians* is a plant pathogen that causes leafy gall disease across a broad range of plants and provides a significant economic burden for the ornamental plant industry (15–17). *R. fascians* is the only plant pathogenic *Rhodococcus* species, where this pathogenicity is determined by the presence of a 199 kb linear virulence plasmid (18, 19) (pFiD188). Infection symptoms are a result of the production of *Rhodococcus* NPs that interfere with plant growth and development. *R. fascians* therefore represents an agriculturally important model for understanding how small molecules mediate microbial interactions in nature (20). pFiD188 harbors multiple BGCs, including the *fas* BGC responsible for the biosynthesis of cytokinins, which induce the growth of differentiated plant tissue and can lead to gall formation (21). The *att* BGC encodes for production of an autoregulatory compound proposed to be involved in the switch to an endophytic phase (22), while the function of a pFiD188 NRPS BGC was unknown (18).

Here, mutase-guided genome mining identified that the NRPS BGC on pFiD188 was predicted to make a putative metalloprotease inhibitor. We show that this BGC (*lyd*) produces the cyclic lipodepsipeptide lydiamycin A, which had been previously isolated from *Streptomyces* spp. as an antimycobacterial antibiotic (23–25), but was not reported to have an HPS-like moiety. The synteny to the actinonin and matlystatin BGCs prompted a detailed revision of the lydiamycin structure to show that it features a rare HPS-like (*R*)-2-pentyl-succinyl chemophore. We show that lydiamycin is a PDF inhibitor via the identification of a self-immunity gene in the *lyd* BGC and demonstrate that lydiamycin production contributes to microbial competition during leaf colonization.

## Results

### Identification of Putative Metalloprotease Inhibitor BGCs.

To identify BGCs that may produce novel HPS-containing NPs, a phylogenetic analysis of all actinobacterial mutases was performed. A coassociation analysis was then performed to determine whether homologues of proposed HPS biosynthesis genes and PKS/NRPS genes were present in the vicinity of mutase genes (*SI Appendix*, Fig. S1), which revealed that a single clade was enriched with potential metalloprotease inhibitor BGCs (Fig. 1*B* and *SI Appendix*, Table S5). This clade contained the known BGCs of actinonin (*Streptomyces* sp. ATCC 14903) and matlystatins (*Actinomadura atramentaria* DSM 43919), as well as uncharacterized BGCs from diverse genera, including *Actinoplanes*, *Micromonospora,* and *Rhodococcus* (*SI Appendix*, Fig. S2).

**Fig. 2.**
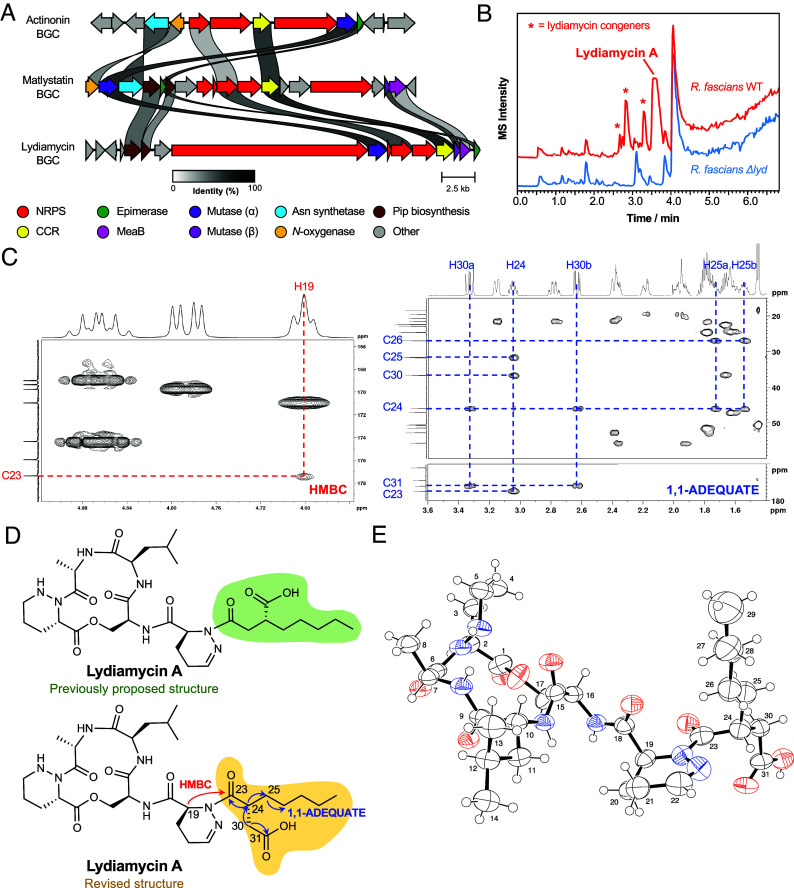
Characterization of lydiamycin from *R. fascians*. (*A*) Similarity comparison of the actinonin, matlystatin, and lydiamycin BGCs. Gene identity percentage is represented by the grayscale links between genes. Pip = piperazic acid. (*B*) Comparison of LC–MS chromatograms of WT R. fascians D188 and a strain with a deletion in the lydiamycin BGC (Δ*lyd*). (*C*) Representative regions of 2D HMBC and 1,1-ADEQUATE NMR spectra with key correlations highlighted for the 2-pentyl-succinyl portion of the molecule. (*D*) Comparison of the previously proposed lydiamycin A structure alongside the revised structure. (*E*) Crystal structure of lydiamycin A. Nitrogen atoms are blue and oxygen atoms are red.

**Fig. 3.**
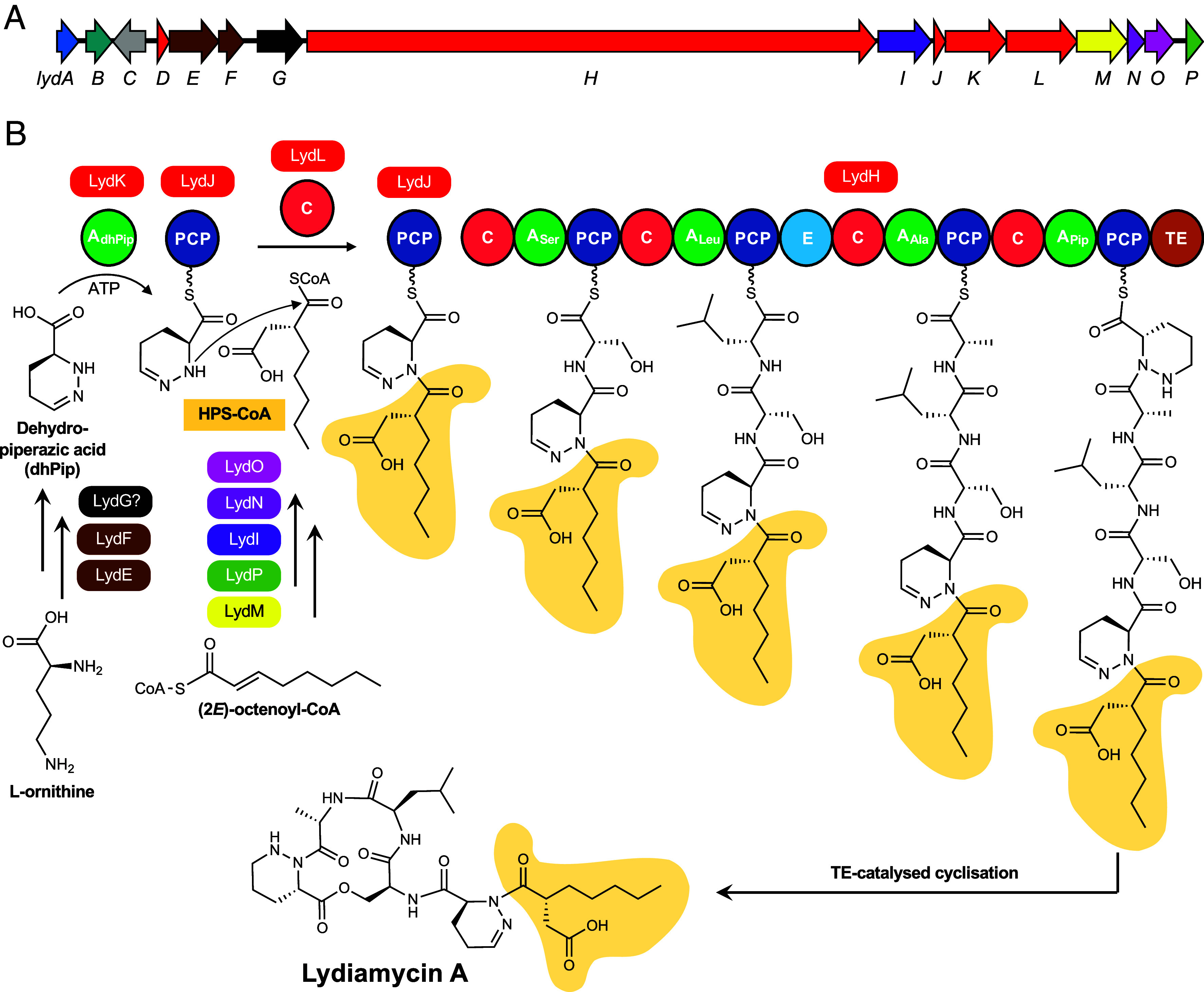
Proposed biosynthesis of lydiamycin A. (*A*) *lyd* BGC is color-coded as in Fig. 2*A* as well as genes encoding a PDF (LydA) in blue, a LuxR-type regulator in teal, a prenyltransferase in gray, a flavin-dependent oxidoreductase in black. See *SI Appendix*, Table S6 for further BGC details. (*B*) Biosynthetic proposal for lydiamycin. The timing of piperazic acid dehydrogenation is not known and may occur postassembly line, given that LydK has an identical recognition sequence to Pip-selective MatJ in matlystatin biosynthesis (*SI Appendix*, Fig. S3).

*R. fascians* D188 was further investigated as it is a well-studied plant pathogen (16) and the BGC is located on the pFiD188 pathogenicity megaplasmid, which implicated the associated NP as a potential pathogenicity determinant, although the product was unknown (18). This BGC (Figs. 2*A* and 3 and *SI Appendix*, Table S6), encodes multiple NRPS-associated proteins (LydDHJKL, *SI Appendix*, Fig. S3), a crotonyl-CoA carboxylase-reductase (CCR, LydM), a hexylmalonyl-CoA epimerase (LydP), α and β subunits of a putative hexylmalonyl-CoA mutase (LydIN) and a MeaB-like protein (LydO), which could aid with mutase function (26). The absence of genes homologous to the asparagine synthetase and *N*-oxygenase genes present in the actinonin and matlystatin BGCs suggested that the *R. fascians* product may instead feature a (*R*)-2-pentyl-succinyl chemophore (Fig. 1*A*) rather than the full hydroxamic acid moiety. The Stachelhaus specificity code (27, 28) of a standalone adenylation domain (LydK) is identical to the matlystatin NRPS MatJ (*SI Appendix*, Fig. S3), which is predicted to activate piperazic acid (13, 29). The predicted incorporation of a piperazic acid residue was supported by the similarity of LydE and LydF to the functionally characterized KtzI/KtzT piperazic acid biosynthetic machinery described in kutzneride biosynthesis (30, 31) (47 and 45% identity, respectively). Taken together, these analyses suggested that this BGC makes a nonribosomal peptide incorporating at least one piperazic acid residue and featuring an intermediate, carboxylated version of the chemophore.

### The *R. fascians* D188 Pathway Makes Lydiamycin A.

To identify the product of the pFiD188 BGC, wild-type (WT) *R. fascians* D188 and a mutant featuring a genetically disrupted adenylation domain (18) (*R. fascians* D188 Δ*lyd*) were fermented in multiple media and sampled at multiple time points. The metabolomes were then compared using liquid chromatography-mass spectrometry (LC–MS), which provided 20 potential products of the BGC (Fig. 2*B* and *SI Appendix*, Figs. S4–S6). Mass spectral networking analysis indicated that many of these compounds are likely to be structurally related (*SI Appendix*, Figs. S7 and S8). The mass of the major BGC-associated compound ([M + H]^+^ *m/z* 664.3680) (*SI Appendix*, Fig. S9) was determined to be identical to lydiamycin A ([M + H]^+^ *m/z* 664.3665; 2.3 ppm). Further LC–MS/MS and NMR spectroscopy analyses of this compound were also fully consistent with previously described lydiamycin A data (23, 25) (*SI Appendix*, Figs. S10–S16 and Tables S7–S9). These data indicate that the major product of the *R. fascians* D188 BGC is lydiamycin A and represents the first identification of the lydiamycin A BGC in a nonstreptomycete. The first lydiamycin BGC was reported (32) from a *Streptomyces* species, which is identical to the *Streptomyces aureoverticillatus* BGC in our phylogenetic analysis (Fig. 2*A*) and is very similar to the *R. fascians* lydiamycin BGC (*SI Appendix*, Fig. S2).

Lydiamycins are cyclic depsipeptides first identified from *S. lydicus* HKI0343 and have been shown to inhibit mycobacteria, including activity toward a multidrug-resistant clinical *M. tuberculosis* strain (23). The published lydiamycin A structure is a macrocyclic pentapeptide with a pentyl-succinic tail (23–25) (Fig. 2*D*) which partially matched our prediction that the D188 BGC product would feature piperazic acid and a terminal carboxylated moiety. The molecular networking data indicate that *R. fascians* D188 makes lydiamycin congeners that are distinct from the previously described lydiamycins A-H (23, 33).

### Bioinformatic Analysis Informs a Structure Revision of Lydiamycin A.

A structural inconsistency of lydiamycin A is apparent when it is compared with actinonin and the matlystatins. Actinonin and matlystatin biosynthesis is proposed to proceed with the condensation of a peptide precursor with the carbonyl of the alpha-proximal thioester of a *N*-hydroxy-2-pentyl-succinamyl-CoA precursor (13) (Fig. 1*A*). However, the published lydiamycin A structure (Fig. 2*D*) necessitates the condensation of a dehydropiperazic acid residue to the carbonyl of the beta-proximal carboxylic acid of a 2-pentyl-succinyl precursor (*SI Appendix*, Fig. S17). This disparity is not supported by the presence of any additional genes in the *lyd* BGC that could rationalize this alternative connectivity. Therefore, we hypothesized that lydiamycin A could be biosynthesized via the alpha-proximal condensation and thus instead features an HPS-like carbon skeleton consistent with actinonin and the matlystatins (Fig. 2*D*).

To test this proposal, a greater quantity of lydiamycin A was purified and analyzed by NMR. A HMBC cross-peak with H19 allowed the distinction of the piperazic-proximal and piperazic-distal carbonyl carbon atoms as the δ_C_ 177.4 ppm and δ_C_ 176.0 ppm peaks, respectively (Fig. 2*C*). Consideration of the relative intensity of HMBC cross-peaks for the 2-pentyl-succinyl carbonyl carbon atoms was more supportive of the revised lydiamycin A structure than the published structure (*SI Appendix*, Fig. S18). To further ratify this proposal, 1,1-ADEQUATE NMR (Fig. 2*C* and *SI Appendix*, Fig. S19) showed that the piperazic-distal carbonyl carbon atom (C31) is adjacent to a carbon with a pair of diastereotopic protons (H30a, H30b; δ_H_ 3.33 and 2.63 ppm) and the piperazic-proximal carbonyl carbon atom (C23) is adjacent to a carbon with a single hydrogen (H24; δ_H_ 3.05 ppm). The proton multiplicity of these key carbon atoms was also confirmed by HSQC (*SI Appendix*, Fig. S20). These observations are consistent only with the proposed revised lydiamycin A structure.

To determine the stereochemistry at C24, we reanalyzed the phenylglycine methyl ester (PGME) derivatization experiments published by Hwang and coworkers on lydiamycin from *Streptomyces* sp. GG23 (25). We applied the modified Mosher methodology described by Yabuuchi and Kusumi for using PGME derivatives to determine the absolute configuration of β, β-disubstituted propionic acids (34). These data were fully consistent with *R* stereochemistry at C24 of lydiamycin (*SI Appendix*, Fig. S21), which is also consistent with the biosynthetic proposal, as the chemophores found in actinonin and matlystatin are stereospecifically biosynthesized as *R* enantiomers (5, 35). To further support this structural revision, we acquired X-ray crystallography data from ~30 µm^3^ crystals of lydiamycin A, which confirmed the structure at a 0.8 Å resolution (Fig. 2*D*).

### Lydiamycin A Inhibits PDF.

Actinonin is a potent inhibitor of PDF (6), whereas the mechanism of action of lydiamycin had not been determined and the structural similarity to actinonin had previously not been noted. We therefore hypothesized that a PDF gene at the edge of the *lyd* BGC (*lydA*) could represent a self-immunity mechanism and that PDF is the molecular target of lydiamycin. Lydiamycin lacks the hydroxamate functionality that enables actinonin and matlystatin to tightly bind active site metals, but the carboxylate functionality of the (*R*)-2-pentyl-succinyl moiety could potentially still bind into the PDF active site.

**Fig. 4.**
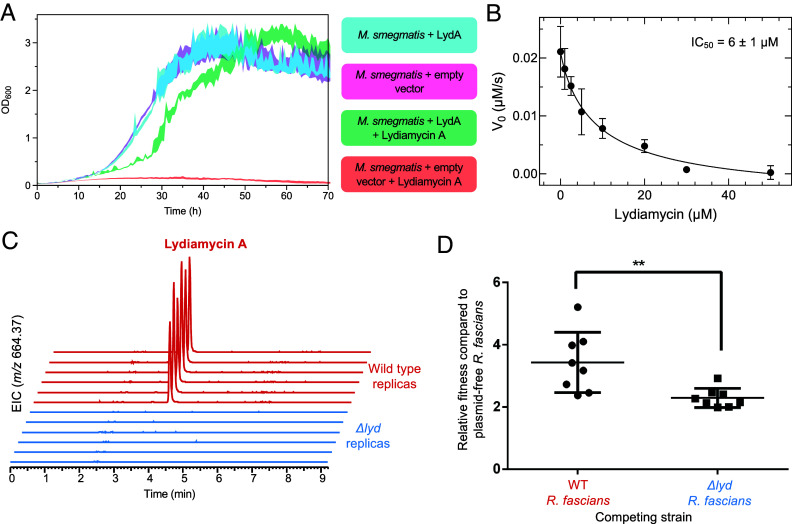
Biological activity of lydiamycin A. (*A*) Growth curves of *M. smegmatis* expressing either empty vector or the PDF gene encoded in the lydiamycin BGC (LydA), in the absence or presence of 50 μM lydiamycin A. The SE of the mean is illustrated by the shaded envelope (n = 3 for controls, n = 6 for experiments with lydiamycin). (*B*) In vitro inhibition of *E. coli* PDF by lydiamycin A. Initial velocities V_0_ of PDF activity with a model peptide (formyl-methionyl-leucyl-p-nitroaniline, *SI Appendix*, Fig. S24) were fitted to a sigmoidal function. Error bars represent the SD of three independent experiments, n = 3. (*C*) Production of lydiamycin A by *R. fascians* during colonization of *N. benthamiana* seedlings. Extracted ion chromatograms (EICs) are shown for *m/z* 664.3665 (± 5 ppm) from LC–MS experiments. (*D*) Competition assay of plasmid-free *R. fascians* (D188-5) against WT and Δ*lyd* strains. The relative fitness of each test strain was calculated as compared to the plasmid-free strain. Individual replicates are indicated as dots. The mean of these replicates is shown as a black cross-bar and error bars indicate the SEM. A two-tailed *t* test showed a significant different between WT and Δ*lyd* relative fitness values (*P* = 0.0068).

Since lydiamycin was previously shown to display antimycobacterial activity (23), the nonpathogenic model organism, *Mycobacterium smegmatis* mc^2^155 was used to assess the role of *lydA* in vivo. Exogenous overexpression of *lydA* rescued *M. smegmatis* to WT-like growth when exposed to 50 μM lydiamycin A (Fig. 4*A*), which was sufficient to prevent growth in the empty vector control or in a strain of *M. smegmatis* overexpressing its native housekeeping PDF gene (*SI Appendix*, Fig. S22). This suggests that the *lydA*-mediated resistance is not caused by a titration of lydiamycin A but rather an inherent resistance to the compound. Given that the *lyd* BGC is encoded on plasmid pFiD188, we assessed the lydiamycin sensitivity of WT *R. fascians* D188 and a pFiD188-free strain (*R. fascians* D188-5). *R. fascians* D188-5 was determined to be at least 60 times more sensitive to lydiamycin A than the *R. fascians* WT strain (MIC = 10 ug/mL versus >600 μg/mL, respectively; *SI Appendix*, Fig. S23). These data indicate that LydA functions as a self-immunity determinant.

To understand whether lydiamycin A directly inhibits PDF, in vitro assays were conducted with *Escherichia coli* PDF (36, 37), which showed that lydiamycin A exhibits dose-dependent inhibition of this PDF with an IC_50_ of 6 μM (Fig. 4*B* and *SI Appendix*, Fig. S24). There is precedence for an intermediate carboxylated group to confer PDF inhibitory bioactivity, as the same moiety is present in the PDF inhibitor Sch-382583 isolated from a *Streptomyces* species (38), while carboxylated variants of actinonin and matlystatin can inhibit PDF, albeit with significantly reduced potencies (39). These data indicate that lydiamycin is an antimycobacterial agent that functions by PDF inhibition. There are no PDF genes in the characterized actinonin or matlystatin BGCs, which means that *lydA* represents a novel self-immunity gene associated with the production of a PDF inhibitor. Our genome mining analysis identified multiple BGCs that encode PDFs (Fig. 1*B*), indicating that this strategy may exist in currently uncharacterized pathways. To identify residues that could potentially contribute to resistance, PDFs that are sensitive to lydiamycin were aligned with BGC-encoded PDFs hypothesized to be resistance to PDF inhibitors (*SI Appendix*, Fig. S25) and then compared a structural model of LydA with a structure of *E. coli* PDF in complex with actinonin (40) (*SI Appendix*, Fig. S26). A potentially important conserved difference close to the substrate binding site is Tyr113, although a Tyr is found in this position in housekeeping PDFs where there is no information on lydiamycin sensitivity (*SI Appendix*, Fig. S25). This position is different to residues that have previously been associated with resistance to PDF inhibitors via mutational studies (41, 42). It is feasible that LydA-mediated resistance involves multiple residues across the PDF, especially as it has moderate (31 to 41%) sequence identity to the three PDFs encoded on the *R. fascians* D188 chromosome, which are predicted to be sensitive based on the activity of lydiamycin toward plasmid-free *R. fascians* D188 (*SI Appendix*, Fig. S23). Detailed biochemical studies will be required to identify the key residues or regions required for resistance.

### Lydiamycin A Is an Important Ecological Agent During Niche Colonization.

As *R. fascians* is the causative agent of leafy gall disease, lydiamycin A was investigated for its involvement in leafy gall disease pathogenesis in *Nicotiana benthamiana* plants (19), especially as actinonin had previously been identified as a chloroplast PDF inhibitor (43). However, there was no significant difference between plant or leafy gall mass for plants infected with either WT *R. fascians* or *R. fascians* Δ*lyd* and grown for 4 wk (*SI Appendix*, Fig. S27). Equivalent results were obtained for assays for the root length of seedlings and gall development on excised leaves (*SI Appendix*, Figs. S28 and S29). These data indicate that the production of lydiamycin A is not required for leafy gall disease development and does not have phytotoxic activity. However, LC–MS analysis of *Nicotiana* seedlings and excised leaves infected with either WT *R. fascians* or *R. fascians* Δ*lyd* (Fig. 4*C*) indicates that lydiamycin A is reliably produced *in planta* during *R. fascians* infection and suggests that lydiamycin may have an ecologically important role.

We hypothesized that the production of lydiamycin during leaf colonization could instead reflect a role in microbial competition, where antibiotic production would reduce the fitness of co-occurring bacteria. This hypothesis was tested by conducting competition assays with plasmid-free *R. fascians* D188-5, which was chosen as it is sensitive to lydiamycin (*SI Appendix*, Fig. S23) and can colonize *Nicotiana* plants. Here, the impact of lydiamycin production was determined by inoculating sterile *N. benthamiana* seedlings with *R. fascians* D188 WT or Δ*lyd* in a 1:1 mixture with plasmid-free (and lydiamycin sensitive) D188-5. After 1 wk of plant growth, bacterial cells were recovered and the relative amounts of each strain were determined. This experiment showed that the WT strain was more effective than Δ*lyd* at out-competing plasmid-free D188-5, based on the relative fitness ratio of WT/D188-5 compared to Δ*lyd*/D188-5 (Fig. 4*D*). A repeat experiment showed a comparable competitive advantage for the WT strain (*SI Appendix*, Fig. S30). These data indicated that lydiamycin production provides a significant fitness benefit (Fig. 4*D*), which is consistent with production of an antibiotic that inhibits the growth of a sensitive competitor. More generally, carriage of pFiD188 provides a significant fitness benefit to *R. fascians* during plant colonization given that D188-5 was outcompeted in both competition assays. These data suggest that lydiamycin A production may be important for antagonizing rival bacteria that compete for similar resources and therefore enhances the likelihood of *R. fascians* successfully colonizing plants.

## Discussion

Metalloproteinase inhibitors are promising lead molecules for a variety of therapeutic targets (44). The HPS moiety in actinonin represents a potent chemophore for the inhibition of multiple therapeutically relevant proteases and hydrolases, including PDF (45). However, currently, few NPs have been discovered with a similar chemophore. Here, mutase-guided genome mining enabled us to identify multiple candidate BGCs that we hypothesized produce protease inhibitors. Our finding that a BGC in *R. fascians* makes lydiamycin was initially unexpected given the dissimilarity between the published lydiamycin structure and actinonin, but extensive NMR analysis, reanalysis of published PGME derivatization data (25), and X-ray crystallography fully supported the structural revision of lydiamycin A (Fig. 2*D*) to show that it features a (*R*)-2-pentyl-succinyl moiety with the same carbon connectivity and stereochemistry as HPS in actinonin.

This finding highlights how consideration of the biosynthetic pathway can be an important tool to inform structural nuances that could be overlooked by chemical derivatization or routine NMR analysis (46), which has previously been demonstrated with the biomimetic synthesis and subsequent structural revision of NPs such as hyperelodione D (47). Multiple previous synthetic and analytical studies had been unable to determine the true connectivity of lydiamycin (23, 24, 33), where each analysis lacked the critical HMBC correlation between C23 and H19. The connectivity at this part of the molecule was instead originally defined via MS/MS fragmentation (23). Subsequent total syntheses of the proposed structures of lydiamycins A and B yielded molecules that were spectroscopically different to the NPs, despite the synthesis of multiple diastereomers (24, 48). These discrepancies were attributed to stereochemical differences in the macrocycle, which were later reassigned by Hwang and coworkers following advanced derivatization and computational analysis that determined that lydiamycins have (*S*)-piperazic acid residues (25), although there was no suspicion that the side chain connectivity was incorrectly assigned.

A full understanding of the structure and stereochemistry of lydiamycin A now enables accurate structure–activity relationship experiments to be undertaken for both the peptide portion and the (*R*)-2-pentyl-succinyl moiety, especially as its affinity to metalloproteinases is predicted to be modulated by changing the carboxylate to a hydroxamate (39). Metabolomics-based identification of multiple novel lydiamycin congeners from *R. fascians* (*SI Appendix*, Figs. S4–S8) provides further natural structural diversity to test for activity, as the masses of these molecules differ to lydiamycin congeners previously identified in other producing organisms (23, 33). Notably, the medium had a significant effect on the array of lydiamycin-like molecules produced. These observations highlight the value of investigating related BGCs in different producers, as they may have the potential to make chemodiverse congeners for SAR studies and pharmacological optimization. For example, novel congeners of the lipodepsipeptide ramoplanin were obtained by the identification of related BGCs (49).

*R. fascians* produces the LydA PDF to confer immunity against lydiamycin. There are several reported methods for bacteria gaining resistance against PDF inhibitors, including by PDF mutations (41), maintaining protein synthesis by bypassing formylation (50, 51), efflux of the inhibitor (52) or PDF overexpression (53). Overexpression of the housekeeping *M. smegmatis* PDF gene did not confer resistance, which infers that LydA is truly resistant to lydiamycin. Further biochemical work is required to understand the precise structural determinants of this LydA immunity, especially as none of the mutations reported in the literature that confer PDF resistance to inhibitors are present in LydA (41, 42) (*SI Appendix*, Fig. S25), suggesting that it could confer resistance in a unique manner. Our sequence and structural analyses of PDFs (*SI Appendix*, Figs. S25 and S26) provide the foundation for prioritizing residues or wider regions for mutational analysis in biochemical experiments. The identification of PDF genes in multiple BGCs indicates that this mechanism of immunity may exist for other PDF inhibitors and that PDF genes may represent underexplored markers of antibiotic BGCs, as highlighted by the recent discovery of gammanonin (54). Coassociation of BGCs with copies of antibiotic target genes represents a promising method for antibiotic discovery and an associated understanding of mechanism of action (55–57), which can be aided by informatic tools such as ARTS (58).

PDFs constitute highly promising antimicrobial targets, given the fitness trade-off observed for acquired resistance phenotypes (51). However, hydroxamate-containing PDF inhibitors, such as actinonin, have potentially not succeeded in clinical trials due to their toxicity, which may be associated with the in vivo conversion of this chemical moiety into mutagenic isocyanates via Lossen rearrangement (59), which has been implicated in the mutagenicity of hydroxamate-based histone deacetylase inhibitors (HDACIs) (60). Our work highlights how multilayered genome mining can select for BGCs producing PDF inhibitors carrying the same chemophore backbone but armed with a nontoxic carboxylate as metal chelator. It is possible that natural PDF inhibitors have similar functional logic as synthetic hydroxamate-based HDACIs. These compounds are normally composed of three parts: a cap group that interacts with the surface of the enzymatic target, a linker, and a metal chelator moiety (60). Given that matlystatins and actinonin share the same HPS chemophore, it is conceivable that the rest of the molecules acts as the specificity determinant cap. Our definition of the lydiamycin NRPS machinery opens the possibility of creating optimized metalloproteinase inhibitors by combining natural specificity-determinant caps and nontoxic carboxylic acid chelators through combinatorial biosynthesis and NRPS engineering (61).

*R. fascians* is an increasingly economically relevant plant pathogen, where it can form long-term biotrophic interactions with the plant host and triggers development of differentiated leafy galls and fasciation (15, 16). The *lyd* BGC is present on the pFiD188 pathogenicity megaplasmid of *R. fascians*, although we found no evidence for lydiamycin involvement in pathogenesis, despite the sensitivity of *Nicotiana* chloroplast PDF to actinonin (43). Instead, leaf-based competition experiments indicate that lydiamycin may play a role in niche colonization (Fig. 4*D*). This activity may indirectly impact the establishment of disease and long-term plant colonization via competition within a complex plant microbiome. Bacterial communities associated with plants are known to be a rich source of antibiotics (62–64), where they can function to control plant disease (65, 66). The production of antibiotics by pathogens themselves is less well understood or investigated, despite the ability of multiple pathogens to make potent antibiotics (67–69). An alternative model for lydiamycin production could relate to plasmid fitness, where the capacity to make an antibiotic and confer resistance to the antibiotic could feasibly help counteract the fitness cost of plasmid carriage (70). Production of a diffusible antibiotic could also deter plasmid-free cheater *Rhodococcus* strains in a mixed population, as it is known that plasmid-free *R. fascians* is capable of effectively colonizing plants (20, 71, 72). These models should be investigated in future experiments with WT and Δ*lyd R. fascians*, such as longer-term plant colonization experiments to assess the impact of lydiamycin production on the population dynamics of synthetic or natural microbial communities, experiments to determine whether antibiotic production coordinates with key steps in *R. fascians* niche establishment (15), and quantification of plasmid maintenance and transmission in diverse environments.

In conclusion, our identification of the lydiamycin BGC on the *R. fascians* pFiD188 pathogenicity plasmid has guided the structural recharacterization of lydiamycin A, the identification of its molecular target and prompted an investigation of its role for plant colonization. Our work should stimulate the rational discovery of further PDF inhibitors, as well as highlighting the potential ecological functions of antibiotic production by microbes.

## Materials and Methods

### General Microbiology Methods.

Chemicals, reagents, and media are described in *SI Appendix* and *SI Appendix*, Table S2. Unless otherwise stated, *R. fascians* strains were grown in LB at 28 °C with shaking at 250 rpm for 24 to 48 h or on LB agar at 28 °C until colonies were visible (2 to 3 d). Unless otherwise stated, *M. smegmatis* cultures were grown aerobically in liquid MOADC at 37 °C with shaking at 250 rpm for 18 to 24 h or on solid MOADC for 3 d. Sterile glass beads and 0.25% TWEEN-80 were added to liquid cultures to reduce cellular aggregation. Unless otherwise stated, *E. coli* strains were grown in LB at 37 °C with shaking at 250 rpm for 16 to 18 h or on LB agar at 37 °C. Plates of all strains were stored at 4 °C. Long-term stocks of all strains were stored at −70 °C in 25% glycerol. gDNA was extracted from *R. fascians* and *M. smegmatis* using the Fast DNA SPIN Kit for Soil (MP Biomedicals), according to the manufacturer’s protocol. The concentration and purity of gDNA, purified amplified DNA, and digested vector backbones were determined using a NanoDrop 2000 spectrophotometer (Thermo Scientific) according to the manufacturer’s protocol.

### Bioinformatic Identification and Analysis of Biosynthetic Gene Clusters.

All actinobacterial protein sequences belonging to the methylmalonyl-CoA mutase IPR006099 InterPro family plus additional sequences absent from the InterPro database were dereplicated to 1,615 representative sequences using the Enzyme Similarity Tool [Enzyme Function Initiative; EFI-EST (73)]. Sequences were aligned using ClustalW (74) and a phylogenetic tree was inferred using RAxML (75) at the CIPRES science gateway (76). Coassociated genes encoding relevant biosynthetic domains were identified using the Enzyme Function Initiative Genome Neighborhood Tool (EFI-GNT) (73) and were mapped to the phylogenetic tree using iTOL (77). The tree can be accessed at https://itol.embl.de/shared/durSMyJ5S9C2. GenBank files associated with BGC-situated mutases (*SI Appendix*, Table S5) were retrieved from the NCBI GenBank database and representative BGCs were selected for visualization using clinker (78) (*SI Appendix*, Fig. S2). Further details of bioinformatic steps are described in *SI Appendix*.

### Bioinformatic Analysis of the LydA PDF.

PDF sequences were obtained from BGCs encoding a PDF, along with sequences of core housekeeping PDFs predicted to have sensitivity to lydiamycin (*E. coli*, *M. smegmatis*, and chromosome-encoded PDFs from *R. fascians*). Additional putative core housekeeping PDF sequences were obtained from the genomes of strains that also feature BGCs with PDFs. Sequences were aligned using Clustal Omega (79) and visualized using Espript 3.0 (80). A structural model of LydA was obtained using ColabFold v1.2 (81). The best scoring model was structurally aligned with *E. coli* PDF bound to actinonin (40) (PDB 1G2A) using Matchmaker in ChimeraX 1.5 (82). Accessions of all proteins are listed in *SI Appendix* along with further experimental details.

### *R. fascians* Metabolite Production.

*R. fascians* D188 and *R. fascians* D188 Δ*lyd* were grown in LB at 28 °C with shaking at 250 rpm for two days. Multiple liquid media (*SI Appendix*, Table S2) were inoculated with 1% v/v of the initial LB cultures to test for differential *R. fascians* metabolite production. Initial comparative metabolomics experiments were conducted as 10 mL cultures in sterile bunged 50 mL falcon tubes in duplicate. Cultures were incubated at 28 °C, 250 rpm and sampled at 5 and 12 d for LC–MS analysis using a Shimadzu Nexera X2 UHPLC coupled to a Shimadzu IT-ToF mass spectrometer. Full experimental details and MS parameters are described in detail in *SI Appendix*.

For mass spectral networking, *R. fascians* D188 WT and Δ*lyd* were grown in 10 mL YEB and SM12 in triplicate in 50 mL sterile bunged falcon tubes. All cultures were incubated at 30 °C with shaking at 250 rpm for 5 d. The resulting samples were subjected to LC–MS/MS analysis using a Waters Acquity UHPLC coupled to a Q-Exactive Orbitrap Mass Spectrometer (Thermo). mzML format data were used to create molecular networks with GNPS (83), which were visualized using Cytoscape 3.8.2 (84). Full experimental details and parameters are described in *SI Appendix*.

### Lydiamycin A Purification.

LB (10 mL) was inoculated with a single colony of WT *R. fascians* D188 and grown for 2 d at 30 °C. This culture was used to inoculate (1% v/v) LB (50 mL) in a 250 mL flask, which was cultured at 30 °C for 1 d with shaking at 250 rpm. This preculture was used to inoculate (1% v/v) 2 × 1 L of SM12 medium in 2 L flasks. These cultures were fermented at 30 °C for 5 d with shaking at 250 rpm. The cultures were centrifuged at 12,000×*g* for 30 min and the resulting supernatant was extracted in two volumes of ethyl acetate for 1 h. The organic extract was washed with an equal volume of water three times and then dried over MgSO_4_. The extract was then dried in vacuo to yield 140 mg of crude material, which was resuspended in MeOH (3 mL) and loaded onto a 30 g Sfär C18 cartridge (Biotage), pre-equilibrated with 5% acetonitrile in water. The sample was fractionated by flash chromatography (Biotage Isolera) and further purified by preparative HPLC using a Dionex UltiMate 3000 HPLC instrument (Thermo Scientific). Full details of chromatography parameters are provided in *SI Appendix*. The major peak was collected and dried in vacuo to afford 20 mg of lydiamycin A as a white powder.

### Lydiamycin A Structural Elucidation.

High-resolution MS/MS data were acquired on a Synapt G2-Si mass spectrometer equipped with an Acquity UPLC (Waters). Full details and parameters are described in *SI Appendix*. For NMR acquisition, pure lydiamycin A (7.0 mg) was dissolved in 200 µL CDCl_3_ and transferred into a 3 mm NMR tube. This sample was subjected to a series of 1D and 2D NMR experiments on a Bruker Avance Neo 600 MHz spectrometer equipped with a TCI cryoprobe at 298 K. The NMR experiments carried out were Proton (16 scans), Carbon (900 scans), HMBC (8 scans), COSY (1 scan), HSQC (6 scans), 1,1-ADEQUATE (197 scans). Spectra were analyzed using Bruker TopSpin 4.0.

### Lydiamycin A Crystal Structure.

A concentrated solution of lydiamycin A was prepared in toluene with gentle heating; then, DMSO was added dropwise close to insolubility. The flask was then sealed and stored at 4 °C and assessed for crystal formation. Suitable crystals were resuspended in 100% ethylene glycol and harvested using Litholoops (Molecular Dimensions), then flash-cooled by plunging into liquid nitrogen prior to transport to the synchrotron. X-ray data were recorded on beamline I04 at the Diamond Light Source (Oxfordshire, UK). The X-ray data were integrated and scaled using DIALS (85). The structure was solved using SHELXT-2018 (86) and refined on *F*^2^ using SHELXL-2018 (87). Two molecules of lydiamycin A were present in the asymmetric unit. Full experimental details for acquisition and solving the structure are described in *SI Appendix*. Data were deposited with the Cambridge Crystallographic Data Centre with reference 2377371 (88).

### PDF Gene Expression in *M. smegmatis* and Lydiamycin A Bioactivity.

Genes encoding the housekeeping *M. smegmatis* PDF (*MsPDF*, WP_011727226.1) and the PDF from the *R. fascians* lydiamycin BGC (*lydA*) were cloned into the pJAM2 vector (89). Electrocompetent *M. smegmatis* mc^2^155 cells were transformed with plasmid DNA, plated onto MOADC agar plates with kanamycin (20 µg/mL), and grown for 1 to 3 d. Resulting strains were grown in 200 µL of MOADC medium in 96-well microplates in triplicate to assess for sensitivity toward 50 µM lydiamycin A. Cultures were incubated at 37 °C with shaking at 500 rpm. Growth was measured by OD_600_ detection every 30 min for 72 h using a SPECTROstar Nano UV/Vis microplate reader (BMG Labtech). Full details of plasmid cloning, procedures for *M. smegmatis* transformation, and bioactivity assays are described in *SI Appendix*.

### In Vitro PDF Inhibition Assay.

PDF from *E. coli* was purified as previously described (37, 90). To determine the inhibitory effect of lydiamycin A on PDF, a colorimetric coupled enzyme assay was carried out using a model peptide, formyl-methionyl-leucyl-p-nitroaniline (fML-pNA, Bachem), as the substrate (36, 91). Formation of *p*-nitroaniline upon deformylation was monitored at 405 nm (ɛ_405_ = 10,600 M^−1^cm^−1^) every 5 s at room temperature. The initial velocity was determined by linear regression and the half-maximal inhibitory concentration (IC_50_) was estimated by sigmoidal fitting. The activity of PDF was verified by Michaelis–Menten kinetics. The determined parameters of K_M_ = 27 µM and k_cat_ = 7 s^−1^ are comparable to published values (37, 91). Full details of enzyme production and assay conditions are described in *SI Appendix*.

### Assays with *Nicotiana* Plants and *R. fascians*.

Methodology used in prior studies of *R. fascians* were adopted for the root growth assay (19), leafy gall assay (92, 93), and excised leaf assay (18). Full experimental details for these assays are described in *SI Appendix*, along with details of spot-on-lawn assays with *R. fascians*.

### Competition Assay.

*N. benthamiana* seedlings grown on plates (½ MS + 3% sucrose + 0.8% agar) were inoculated with 4 µL (approximately 2 × 10^5^ cells) of *R. fascians* D188-5 + WT and *R. fascians* D188-5 + Δ*lyd* that had been separately grown in LB. In the first experiment, 8 groups of 7 seedlings were infected per condition. In the second experiment, 7 groups of 8 seedlings per timepoint were infected per condition. The plates were grown under 16/8 h light/dark at 28 °C. In the first experiment, bacteria were isolated at 7 d postinoculation (dpi), whereas in the second experiment, bacteria were isolated at 3, 7, and 14 dpi. Colony-forming units (CFU) were measured for each sample with streptomycin selection (isolation of D188-5 only) and without selection (isolation of both strains). CFU/mL data were then used to determine relative fitness of competing strains. Full experimental details are described in *SI Appendix*.

## Supplementary Material

Appendix 01 (PDF)

## Data Availability

Mass spectrometry data are available in the MassIVE repository with accession MSV000097741 (94). All other data are included in the manuscript and/or *SI Appendix*.
