# Supplementary material for: Identification of the lydiamycin biosynthetic gene cluster in a plant pathogen guides structural revision and identification of molecular target
Source: Proc Natl Acad Sci U S A. 2025 May 19;122(21):e2424388122. doi: 10.1073/pnas.2424388122 (PMC12130866; doi:10.1073/pnas.2424388122)
Supplement: Supplementary file 1 — Appendix 01 (PDF) [file pnas.2424388122.sapp.pdf]

SUPPORTING INFORMATION

Identification of the lydiamycin biosynthetic gene cluster in a plant pathogen guides structural revision and identification of molecular target

Jonathan J. Ford[1], Javier Santos-Aberturas[1,2], Edward S. Hems[1,2], Joseph W. Sallmen[1], Lena A. K. Bögeholz[3], Guy Polturak[4‡], Anne Osbourn[4], Joseph A. Wright[5], Marina V. Rodnina[3], Danny Vereecke[6], Isolde M. Francis[7], Andrew W. Truman[1,2*]

1. Department of Molecular Microbiology, John Innes Centre, Norwich, NR4 7UH, United Kingdom.
2. Centre for Microbial Interactions, Norwich Research Park, Norwich, NR4 7UG, United Kingdom.
3. Department of Physical Biochemistry, Max Planck Institute for Multidisciplinary Sciences, 37077, Göttingen, Germany.
4. Department of Biochemistry and Metabolism, John Innes Centre, Norwich, NR4 7UH, United Kingdom.
5. School of Chemistry, University of East Anglia, Norwich Research Park, Norwich, NR4 7TJ, United Kingdom.
6. Howest University of Applied Sciences, 8200 Bruges, Belgium.
7. Department of Biology, California State University, Bakersfield, California 93311, USA.

‡ Current affiliation: Institute of Plant Sciences and Genetics in Agriculture, The Hebrew University of Jerusalem, Rehovot, 7610001, Israel.

***Corresponding author**
andrew.truman@jic.ac.uk

MATERIALS AND METHODS

Chemicals and reagents

All chemicals and media components were purchased from Sigma Aldrich, except for the following: agar (Melford), NaCl and glucose (Fisher Scientific), yeast extract (Merck), soya flour (Holland and Barrett) and peptone (BD Biosciences). All enzymes were supplied by New England Biolabs (NEB) unless otherwise specified. All solvents for extractions and chromatography were supplied by Fisher Scientific. Ultrapure water was obtained using a Milli-Q purification system (Merck). All media were autoclaved prior to use and chemical solutions were filter sterilised using a 0.22 µM syringe filter. All media used are defined in Table S2 and were made up in 1 L of Milli-Q water. Solid media were made with 2% agar unless stated otherwise. Antibiotics were added where necessary to the following concentrations: kanamycin (50 µg/mL for *E. coli* and 20 µg/mL for *M. smegmatis*); chloramphenicol (25 µg/mL), streptomycin (100 µg/mL). All primers were synthesised by Eurofins Genomics or Integrated DNA Technologies at a 25 nmol synthetic scale with standard desalting. Primers were diluted to 100 µM in Milli-Q water and stored at -20 °C. Working stocks were prepared at 10 µM and stored at 4 °C.

Identification and phylogeny of actinobacterial acyl-CoA mutases

The MatB protein sequence (WP_019634562.1) was used in an InterPro[1] search to identify the methylmalonyl-CoA mutase InterPro entry (IPR006099). All actinobacterial protein sequences belonging to the IPR006099 InterPro entry (~13,000 sequences at time of analysis, February 2022) were downloaded in .FASTA format. Mutase protein sequences identified in a MatB BLAST[2] analysis that were highly similar (>60% identity) but absent in the InterPro database were added to the sequence list (WP_019634562.1, WP_176725504.1, WP_210837750.1, WP_063760750.1, WP_189742219.1, WP_223771548.1, WP_104814753.1, WP_184992507.1, QES31823.1,

QNF54062.1, QHF95772.1). Sequences were submitted to the Enzyme Similarity Tool (Enzyme Function Initiative; EFI-EST[3]) to produce a protein sequence similarity network (SSN). Edges were drawn between nodes where the similarity e-value was less than e^{-50}. Protein sequences of >40% sequence similarity were collated into 1,615 representative nodes to generate the SSN. The SSN was exported and visualised using Cytoscape[4]. The SSN node table was exported as a .csv file and the UniProt retrieval tool was used to obtain the protein sequences of the first accession number of each representative node ("*representative FASTA file*"). Representative accessions associated with the chemophore clade are shown in Table S5. The resulting protein sequences were then aligned using ClustalW[5] with default parameters at the CIPRES science gateway[6]. A tree was then constructed with this alignment file using the 'RAxML-HPC Blackbox' method[7] with default parameters at the CIPRES science gateway[6], which determined that a bootstrap value of n=350 was sufficient. The tree was then visualised using iTOL[8].

Co-association analysis of mutases and other genes

In order to perform a co-association analysis with one protein sequence from each representative node of the SSN described above, the *representative FASTA file* was submitted to EFI-EST[3] to generate a network lacking clustering (ID=100%). This SSN was then submitted to the Enzyme Function Initiative Genome Neighbourhood Tool (EFI-GNT)[3]. Parameters of neighbourhood size = 15 and minimal co-occurrence percentage lower limit = 20 were set. The resultant SSN was viewed in Cytoscape[4] and the node table exported as a .csv file, which was then assessed for Pfam domains of interest for each accession number. The Pfam domains of interest were as follows: PF00107 (CCR), PF13669 (acyl-CoA epimerase), PF02310 (acyl-CoA mutase β subunit), PF03308 (MeaB-like), PF00733 (asparagine synthetase), PF11583 (*N*-oxygenase), PF13434 (KtzI-like; piperazic acid biosynthesis), PF04299 (KtzT-like; piperazic acid biosynthesis) PF02770 (acyl-CoA dehydrogenase), PF00501 (NRPS, adenylation), PF00668 (NRPS, condensation), PF00109 (PKS, ketosynthase), PF00550 (carrier protein), PF00975 (thioesterase), PF07690 (transporter), and PF01327 (peptide deformylase). Co-associated protein domains were mapped to the phylogenetic tree using iTOL[8]. The tree can be accessed at https://itol.embl.de/shared/durSMyJ5S9C2.

Phylogeny and co-association analysis of the BGC clade

A clade was identified in the above analysis that co-associated with multiple biosynthetic Pfam domains. The representative nodes of this clade were therefore subjected to another phylogenetic and co-association analysis. All protein sequences from each representative node were retrieved from the original EFI-EST SSN, in addition to a phylogenetic outgroup (UniProt accession A0A166QLU4). The >60% identity sequences absent in the UniProt database were also added, as detailed above. In total, 45 sequences were submitted to CIPRES for ClustalW alignment and RAxML-HPC Blackbox tree building, using default parameters. The program determined that a bootstrap value of n=300 was sufficient and de-replicated 20 sequences that were 100% identical. The co-association analysis was performed using the same parameters as detailed above. This co-association analysis was converted into a binary annotation file and displayed on the tree using iTOL[8]. The tree was manually trimmed to remove phylogenetically identical acyl-CoA mutase leaves that had identical co-association results. GenBank sequence files associated with each mutase (see Table S5) were retrieved from the NCBI GenBank database and representative BGCs were selected for visualisation using clinker[9].

Bioinformatic analysis of the lydiamycin BGC

antiSMASH 6.2[10] was used for general analysis of the lydiamycin BGC. NRPSsp[11], PKS/NRPS Analysis[12] and NRPSpredictor2[13] were used for predicting the adenylation specificity of the *lyd* NRPS genes. Stachelhaus sequences were obtained from the antiSMASH output.

Bioinformatic analysis of the LydA peptide deformylase

Peptide deformylase (PDF) sequences were obtained from a selection of BGCs encoding a PDF, along with sequences of core housekeeping PDFs predicted to have sensitivity to lydiamycin (*E. coli*, *M. smegmatis*, plus chromosomally-encoded PDFs from *R. fascians*). Additional putative core housekeeping PDF sequences were obtained from the genomes of strains that also feature BGCs with PDFs (*Micromonospora* sp. C31, *Streptomyces venezuelae* ATCC 14583 and *Streptomyces* sp. CFMR-7). In total, the following sequences were obtained. The naming used in Figure S25 is shown:

AET25244.1 (LydA_BGC_PDF)
UUG66826.1 (Streptomyces_Lyd_BGC_PDF)
QES30052.1 (S_venezuelae_BGC_PDF)
ALC26904.1 (CFMR-7_BGC_PDF)
WP_210837776.1 (Micromonospora_C31_BGC_PDF)
WP_113847892.1 (E_coli_house_PDF)
WP_011727226.1 (M_smegmatis_house_PDF)
AMY54002.1 (D188_house_PDF1)
AMY54659.1, (D188_house_PDF2)
AMY55275.1 (D188_house_PDF3)
QES29386.1 (S_venezuelae_house_PDF1)
QES31188.1 (S_venezuelae_house_PDF2)
ALC27624.1 (CFMR-7_house_PDF1)
ALC31120.1 (CFMR-7_house_PDF2)
WP_029395812.1 (CFMR-7_house_PDF3)
WP_210834615.1 (Micromonospora_C31_house_PDF1)
WP_210838500.1 (Micromonospora_C31_house_PDF2)
WP_210832813.1 (Micromonospora_C31_house_PDF3)

Sequences were aligned using Clustal Omega[14] with default settings and visualised using Espript 3.0[15]. A structural model of LydA was obtained using ColabFold v1.2[16] (AlphaFold2[17] using MMseqs2[18]) with default settings. The best scoring model was structurally aligned with *E. coli* PDF bound to actinonin[19] (PDB 1G2A) using Matchmaker with default settings in ChimeraX 1.5[20].

Initial screening of *R. fascians* metabolite production

Rhodococcus fascians D188 and *R. fascians* D188 Δ*lyd* were grown in liquid LB (Table S2) at 28 °C, 250 rpm under aerobic conditions. *R. fascians* D188 Δ*lyd* was first described in Francis *et al.*[21] as Δ*nrp5.* Pre-cultures were grown for 2 days and 1% used for inoculations. The following liquid media were selected for testing *R. fascians* metabolite production and are described in Table S2: aurachin production medium[22], rhodostreptomycin production medium[23], minimal media A (MinA)[24], screening medium 7 (SM7), SM12, yeast extract broth (YEB), actinonin production medium[25], matlystatin production medium[25], and bottromycin production medium[26]. In the comparative metabolomics experiment, 10 mL of each of the above media, in sterile bunged 50 mL falcon tubes, were inoculated with 1% *R. fascians* D188 WT and *R. fascians* D188 Δ*lyd* pre-cultures in duplicate.

Samples were incubated at 28 °C, 250 rpm and sampled at 5 and 12 days by removing 0.75 mL of culture. This was extracted in 0.75 mL MeOH (shaken, 1 hour), centrifuged (13,000 rpm, 30 minutes) and 0.6 mL of supernatant aliquoted for LC-MS analysis. Medium-only samples were prepared using the same method.

Samples were subjected to LC-MS analysis using a Shimadzu Nexera X2 UHPLC coupled to a Shimadzu IT-ToF mass spectrometer. Samples (5 µL) were injected onto a Phenomenex Kinetex 2.6 µm C18 column (50 x 2.1 mm, 100 Å), eluting with a linear gradient of 5 to 100% acetonitrile in water + 0.1% formic acid (FA) over 6 minutes with a flow-rate of 0.6 mL/min at 40 °C. Positive mode mass spectrometry data were collected between *m/z* 300 and 2000 with an ion accumulation time of 10 ms featuring an automatic sensitivity control of 70% of the base peak. The curved desolvation line temperature was 300 °C and the heat block temperature was 250 °C. MS/MS data was collected in a data-dependent manner using collision-induced dissociation energy of 50% and a precursor ion width of 3 Da. The instrument was calibrated using sodium trifluoroacetate cluster ions prior to every run. LabSolutions software (Shimadzu) was used to identify molecules present in WT *R. fascians* and absent in *R. fascians* Δ*lyd*.

Mass spectral networking

Single colonies of *R. fascians* D188 WT and Δ*lyd* strains were used to inoculate 10 mL LB and grown for 2 days at 30 °C. These cultures were used to inoculate (1% v/v) 10 mL YEB and SM12 media in in triplicate in 50 mL sterile bunged falcon tubes. Media only samples were also prepared in triplicate. All cultures were incubated at 30 °C with shaking at 250 rpm for 5 days. 0.75 mL aliquots of the cultures were extracted in 0.75 mL MeOH for 1 hour, with shaking. Samples were centrifuged (15,871 x *g*) for 30 minutes. The resulting supernatants were subjected to LC-MS/MS analysis using a Waters Acquity UHPLC coupled to a Q-Exactive Orbitrap Mass Spectrometer (Thermo). Samples (5 µL) were injected onto a Phenomenex Kinetex 2.6 µm C18 column (50 x 2.1 mm, 100 Å), eluting with a linear gradient of 5 to 95% acetonitrile in water + 0.1% FA over 6 minutes with a flow rate of 0.6 mL/min at 40 °C. Positive mode mass spectrometry data was collected between *m/z* 150 and 2000. MS/MS data were collected in a data-dependent manner with the following parameters: chromatography peak width = 7 s; resolution = 70,000, AGC target = $3x10^6$, maximum IT = 100 ms, scan range 150 to 2000 *m/z*; dd-MS2 settings: resolution 17,500, AGC target = $1x10^5$, maximum IT = 50 ms, loop count = 5, isolation window 1.5 *m/z*, isolation offset = 0.0 *m/z*, stepped Normalized Collision Energy = 20, 40, 60; dd settings: minimum AGC target = $8x10^3$, Intensity threshold = 1.6 $x10^5$, exclude isotopes = ON, dynamic exclusion = 1 s.

Data was converted to mzML format and molecular networks were created using the online workflow on the GNPS website[27,28]. The data was filtered by removing all MS/MS fragment ions within +/- 17 Da of the precursor m/z. MS/MS spectra were window filtered by choosing only the top 6 fragment ions in the +/- 50 Da window throughout the spectrum. The precursor ion mass tolerance was set to 0.1 Da and a MS/MS fragment ion tolerance of 0.1 Da. A network was then created where edges were filtered to have a cosine score above 0.7 and more than 6 matched peaks. Further, edges between two nodes were kept in the network if and only if each of the nodes appeared in each other's respective top 10 most similar nodes. Finally, the maximum size of a molecular family was set to 100, and the lowest scoring edges were removed from molecular families until the molecular family size was below this threshold. The spectra in the network were then searched against GNPS spectral libraries. The library spectra were filtered in the same manner as the input data. All matches kept between network spectra and library spectra were required to have a score above 0.7 and at least 6

matched peaks. The network was visualised using Cytoscape (version 3.8.2). The GNPS results are available at:
https://gnps.ucsd.edu/ProteoSAFe/status.jsp?task=648c5adcb06b4733ba6ef092bcad9153

Chromatography conditions for lydiamycin A purification

Following the purification procedure reported in the main text, the initial flash chromatography fractionation on a Biotage Isolera was conducted at 12 mL/min with UV monitoring at 210 and 237 nm. Mobile phase A: water; mobile phase B: acetonitrile. Elution started from 5% B with a gradient to 100% B over 10 column volumes (CV), and then holding at 100% B for 3 CV. The fractions were analysed by LC-MS and those containing lydiamycin A were combined, dried *in vacuo* and further purified by preparative HPLC using a Dionex UltiMate 3000 HPLC instrument (Thermo Scientific). To do this, the sample was dissolved in MeOH to 9 mg/mL, filtered using a 0.45 µm PTFE filter (Whatman) and applied to a Gemini-NX C18 (150 mm x 21.1 mm) column (Phenomenex). The mobile phase used was A: water with 0.1% formic acid; mobile phase B: acetonitrile with 0.1% formic acid; flow rate 20 mL/min; injection volume 500 µL; elution gradient: T = 0 min, 5% B; T = 2 min, 5% B; T = 7 min, 45% B; T = 23 min, 70% B; T = 25 min, 95% B; T = 90 min, 95% B; T = 30.1 min, 5% B; T = 33 min, 5% B; UV absorbance monitoring at 237 nm. The major peak was collected and dried *in vacuo* to afford 20 mg of lydiamycin A as a white powder.

Lydiamycin A structural elucidation MS/MS parameters

High-resolution mass spectra were acquired on a Synapt G2-Si mass spectrometer equipped with an Acquity UPLC (Waters). Aliquots of the samples were injected onto an Acquity UPLC® BEH C18 column, 1.7 µm, 1x100 mm (Waters) and eluted with a gradient of acetonitrile/0.1% FA (B) in water/0.1% FA (A) with a flow rate of 0.08 mL/min at 45 °C. The concentration of B was kept at 1% for 1 min followed by a gradient up to 40% B in 9 min, ramping to 99% B in 1 min, kept at 99% B for 2 min and re-equilibrated at 1% B for 4 min. MS data were collected in positive mode with the following parameters: resolution mode, positive ion mode, scan time 0.5 s, mass range *m/z* 50-1200 calibrated with sodium formate, capillary voltage = 2.5 kV; cone voltage = 40 V; source temperature = 125 °C; desolvation temperature = 300 °C. Leu-enkephalin peptide was used to generate a lock-mass calibration with 556.2766, measured every 30 s during the run. For MS/MS fragmentation, a data directed analysis (DDA) method was used with the following parameters: precursor selected from the 4 most intense ions; MS2 threshold: 5,000; scan time 0.5 s; no dynamic exclusion. In positive mode, collision energy (CE) was ramped between 10-30 at low mass (*m/z* 50) and 15-60 at high mass (*m/z* 1200).

Lydiamycin A X-ray data collection and analysis

X-ray data were recorded on beamline I04 at the Diamond Light Source (Oxfordshire, UK) using an Eiger2 16M detector (Dectris) with the crystal maintained at 100 K by a Cryojet cryocooler (Oxford Instruments). The crystals diffracted very strongly, but at an X-ray wavelength of 0.6888 Å, the maximum resolution of the inscribed circle at the minimum possible detector distance was only 0.95 Å. In order to obtain complete data beyond this resolution, diffraction data were taken from two separate crystals, and for each of these, 4 x 360° passes were recorded at increasing χ offsets of 0, 15, 30 and 45°. Each pass comprised 3600 x 0.1° images with an estimated total dose of 0.5 MGy.

The X-ray data were integrated and scaled using DIALS[29]. Cell parameters for each sweep were refined independently using all strong indexed reflections. All sweeps were combined into a single file and reflections were scaled but not merged. An absorption correction was applied using the

Blessing model and a median unit cell, calculated from the individual sweeps, was applied to the output file. Data beyond 0.8 Å resolution were excluded from the subsequent analysis. The space group was *C*2 with cell parameters of a = 20.9, b = 14.7, c = 24.2 Å, β = 97.3°. The structure was solved using SHELXT-2018[30] and refined on F^2 using SHELXL-2018[31]. Two molecules of lydiamycin A were present in the asymmetric unit. In both molecules, the carbonyl oxygen (O(1)) of the ester linkage was disordered over two positions. In one of the molecules, the terminal CH3CH2 fragment of the pentyl side-chain (C(28) and C(29)) was disordered over two positions. After completion of the model for lydiamycin A, voids remained in the structure. A solvent mask was calculated in Olex2[32], and 262 electrons were found in a volume of 822 Å^3 in 2 voids per unit cell. This is consistent with the presence of six water molecules per asymmetric unit which account for 240 electrons per unit cell. At the completion of refinement, the values of the Flack and Hooft parameters were ambiguous: 0.97(18) and –0.2(2), respectively. Absolute configuration was therefore assigned by consideration of the known centres C(2), C(7), C(10), C(16) and C(19), and the NMR data for assignment of C(24). At the conclusion of refinement, R^1 = 6.45%, wR^2 = 19.73%, GOOF = 1.067.

Generation of pJAM vectors for PDF gene expression in *M. smegmatis*

Genes encoding the housekeeping *M. smegmatis* PDF (*MsPDF*, WP_011727226.1) and the PDF from the *R. fascians* lydiamycin BGC (*lydA*) were amplified from gDNA using Q5 polymerase (NEB) and a T100 Thermal Cycler (Bio-Rad). PCR reactions were separated by gel electrophoresis and purified using the GFX PCR DNA and Gel Band Purification Kit (Cytiva) according to the manufacturer's protocol. The *lydA* gene and pJAM2 vector[33] were digested with BamHI and XbaI and then gel purified. *lydA* was ligated into pJAM2 using T4 DNA ligase (Thermo) and the reaction was used to transform chemically competent *E. coli* DH5α cells. *MsPDF* was inserted into pJAM2 using NEBuilder HiFi DNA Assembly Master Mix (NEB). The reaction was incubated at 50 °C for 60 minutes and 2 µL of the reaction mixture used to transform chemically competent *E. coli* DH5α cells. All vectors were sequenced by Eurofins Genomics using a Mix2Seq Kit.

Genetic manipulation of *M. smegmatis*

10 mL of MOADC (Table S2) was inoculated with a single colony of *M. smegmatis* and grown overnight at 37 °C. 100 mL of MOADC (in 250 mL flask) was inoculated with the pre-culture (1% v/v) and grown at 37 °C until an OD_{600} of 0.2-0.8 was reached. The culture was incubated on ice for 1.5 hours. The following steps were performed at 4 °C or on ice. The culture was transferred to two 50 mL tubes and centrifuged at 5,000 x *g* for 10 minutes. The supernatant was discarded by decanting. Each pellet was resuspended in 50 mL sterile 10% glycerol and centrifuged as above. The supernatant was discarded by decanting. Each pellet was resuspended in 25 mL sterile 10% glycerol and centrifuged at 2,000 x *g* for 10 minutes. The supernatant was discarded by decanting. This wash step was repeated once more. Each pellet was resuspended in 500 µL sterile 10% glycerol and 50 µL aliquots were dispensed into microcentrifuge tubes. These were flash frozen in liquid N_2 and stored at -70 °C. Electrocompetent cells were thawed on ice and mixed with 1.0 – 5.0 µL of plasmid DNA. This was transferred to a 2 mm electroporation cuvette and placed on ice for 1 minute. The outside of the cuvette was dried, and it was ensured that no bubbles had formed. The cells were electroporated at 25 µFD, 2.5 KV, 1000 Ω. The cells were mixed with 1 mL MOADC medium and transferred to a 15 mL tube. The cells were incubated at 37 °C for 2 hours with shaking at 250 rpm. 0.1 – 1.0 mL of the culture was plated onto MOADC agar plates (with appropriate antibiotic) and grown for 1-3 days.

Lydiamycin A bioactivity with *M. smegmatis*

10 mL of MOADC with kanamycin (20 µg/mL) was inoculated with a single colony of *M. smegmatis* mc^2155 (containing either empty pJAM2 or pJAM2 harbouring a PDF, Table S4) and grown overnight at 37 °C with shaking at 250 rpm. The *M. smegmatis* cultures were inoculated into 200 µL of MOADC medium in 96-well microplates to a final concentration of OD_{600} 0.05, with between three and six replica cultures, depending on the experiment. All cultures were supplemented with 0.25% TWEEN-80, 0.2% acetamide and kanamycin (20 µg/mL). Each strain was grown either in the presence of methanol (5% v/v) or 50 µM lydiamycin A (in 5% v/v methanol), unless otherwise stated. Cultures were incubated at 37 °C with shaking at 500 rpm. Growth was measured by OD_{600} detection every 30 minutes for 72 hours using a SPECTROstar Nano UV/Vis microplate reader (BMG Labtech). The data were processed in Excel (Microsoft) and Datagraph 5.2 (Visual Data Tools).

***In vitro* PDF inhibition assay**

PDF from *E. coli* was purified as previously described[34,35]. In short, His_6-tagged PDF was expressed in *E. coli* BL21(DE3)pLysS cells (New England Biolabs) and purified by Cobalt-Talon affinity chromatography (Clontech) followed by ion exchange chromatography using a HiTrapQ column (Cytiva). PDF was stored in HEPES buffer (25 mM HEPES, pH 7.5, 70 mM NH_4Cl, 30 mM KCl, 7 mM $MgCl_2$, 0.2 mM $CoCl_2$, 1 mM TCEP, and 10% (v/v) glycerol) at -80 °C. To determine the inhibitory effect of lydiamycin on PDF, a colorimetric coupled enzyme assay was carried out using a model peptide, formyl-methionyl-leucyl-p-nitroaniline (fML-pNA, Bachem), as the substrate. PDF (10 nM) was incubated with fML-pNA (20 µM) and *Aeromonas* aminopeptidase (0.8 U/mL) in the presence of lydiamycin A (0-50 µM) in HEPES buffer with 1% DMSO[36,37]. Formation of *p*-nitroaniline upon deformylation was monitored at 405 nm (ε_{405} = 10,600 $M^{-1}cm^{-1}$) every 5 s at room temperature. The initial velocity was determined by linear regression and the half maximal inhibitory concentration (IC50) was estimated by sigmoidal fitting. The activity of PDF in the presence of 1% DMSO was verified by Michaelis Menten kinetics.

Spot-on-lawn assay with *R. fascians*

10 mL of LB was inoculated with a single colony of *R. fascians* and grown overnight at 28 °C with shaking at 250 rpm. This pre-culture was used to inoculate 50 mL of LB (in 250 mL flasks), which was grown at 28 °C with 250 rpm shaking until an OD_{600} of 0.40 was reached. SNA medium was melted and cooled to 42 °C in a water bath. The *R. fascians* culture was added to SNA at a concentration of OD_{600} 0.02 and poured into 120 x 120 mm plates (50 mL each). A concentration series was prepared by serial dilution of pure lydiamycin A in methanol from 600 µg/mL to 1 µg/mL. A positive control of 50 µg/mL kanamycin in water was prepared. 5 µL spots were applied to the plates and allowed to dry. The plates were incubated at 37 °C for 1-3 days and imaged.

Root growth assay

A root growth assay was conducted based on methodology in a prior study of *R. fascians*[38]. 120 x 120 mm plates were prepared, each with 50 mL of ½ MS + 3% sucrose + 0.8% agar media. *N. benthamiana* seeds were surface sterilised in 1.5% bleach solution for 30 minutes with strong vortexing. Under aseptic conditions, the seeds were washed in sterile Milli-Q water four times and aliquoted onto sterile filter paper. Twenty seeds were placed on each plate (in a line 2 cm from one edge). Plates were closed with micropore tape and stored in the dark at 4 °C for 3 days to align germination. The plates were placed vertically and grown under 16/8 hour light/dark at 28 °C for 5 days.

10 mL of LB was inoculated with a single colony of *R. fascians* and grown overnight at 30 °C with shaking at 250 rpm. 0.5 mL of culture was centrifuged (4,000 x *g* for 10 minutes), the supernatant discarded by decanting and the pellet resuspended in 1 mL sterile Milli-Q water. This step was repeated once more, and the pellet resuspended in sterile 100 mM $MgCl_2$ solution to an OD_{600} value of 0.5. The length of the plant root was marked on the plate. Each seedling was inoculated with 4 µL of culture. Each *R. fascians* strain and a mock infection using 4 µL of 100 mM $MgCl_2$ was tested with 60 seedlings. The plates were closed with micropore tape and returned to the same growth conditions for 7 days. The length of each plant root was marked and the distance between the root length at 0 and 7 days-post-inoculation (dpi) was measured. Data were processed using GraphPad Prism.

LC-MS analysis of seedlings

Following the root length assay described above, six groups of five seedlings from each condition were transferred separately to 2 mL centrifuge tubes containing 1 mL MeOH and shaken vigorously for 30 minutes. The tubes were centrifuged at 15,871 x *g* for 30 minutes and the resulting supernatants were analysed by LC-MS using instrument settings as described above for mass spectral networking.

Leafy gall assay

A leafy gall assay was conducted based on methodology from prior studies of *R. fascians*[39,40]. GA-7 Magenta vessels (Sigma) were prepared, each with 150 mL of ½ MS + 3% sucrose + 0.8% agar media. *N. benthamiana* seeds were surface sterilised in 1.5% bleach solution for 30 minutes with strong vortexing. Under aseptic conditions, the seeds were washed in sterile Milli-Q water four times and aliquoted onto sterile filter paper. One seed was placed into each Magenta vessel, and these were closed and grown with 16/8 hour light/dark at 28 °C for 6 weeks. 10 mL of LB was inoculated with a single colony of *R. fascians* and grown overnight at 30 °C with shaking at 250 rpm. 0.5 mL of culture was centrifuged (4,000 x *g* for 10 minutes), the supernatant discarded by decanting and the pellet resuspended in 1 mL sterile Milli-Q water. This step was repeated once more, and the pellet resuspended in sterile 100 mM $MgCl_2$ solution to an OD_{600} value of 0.5. The meristem of each plant was wounded with sterile forceps and infected with 5 µL of WT *R. fascians* culture, *R. fascians* Δ*lyd* culture or sterile 100 mM $MgCl_2$ solution (n = 9 plants each). The plants were returned to the same growth conditions as above for four weeks and imaged. The galls were also removed, and the masses of gall and plant measured.

Excised leaf assay

An excised leaf assay was conducted based on methodology in a prior study of *R. fascians*[21]. GA-7 Magenta vessels (Sigma) were prepared, each with 150 mL of ½ MS + 3% sucrose + 0.8% agar media. *Nicotiana tabacum* cv. Samsun NN seeds were surface sterilised in 1.5% bleach solution for 30 minutes with vortexing. Under aseptic conditions, the seeds were washed in sterile Milli-Q water four times and aliquoted onto sterile filter paper. One seed was placed into each Magenta vessel, and these were closed and grown with 16/8 hour light/dark at 28 °C for 4 weeks. Leaves were aseptically excised and placed onto 120 x 120 mm ½ MS + 3% sucrose + 0.8% agar plates. 10 mL of LB was inoculated with a single colony of *R. fascians* and grown overnight at 30 °C with shaking at 250 rpm. Leaves were infected with 5 µL of WT *R. fascians* culture, *R. fascians* Δ*lyd* culture or sterile 100 mM $MgCl_2$ solution (n = 15 leaves each). The plates were returned to the same growth conditions as above for three weeks and imaged.

Competition assay

120 x 120 mm plates were prepared, each with 50 mL of ½ MS + 3% sucrose + 0.8% agar media. *N. benthamiana* seeds were surface sterilised in 1.5% bleach solution for 30 minutes with strong vortexing. Under aseptic conditions, the seeds were washed in sterile Milli-Q water four times and aliquoted onto sterile filter paper. 36 seeds were placed onto each plate. Plates were closed with micropore tape and stored in the dark at 4 °C for 3 days to align germination. The plates were grown under 16/8 hour light/dark at 28 °C for 7 days. 10 mL of LB was inoculated with a single colony of *R. fascians* and grown overnight at 30 °C with shaking at 250 rpm. 0.5 mL of culture was centrifuged (4,000 x *g* for 10 minutes), the supernatant discarded by decanting and the pellet resuspended in 1 mL sterile Milli-Q water. This step was repeated once more, and the pellet resuspended in sterile 100 mM $MgCl_2$ solution to an OD_{600} value of 0.5. 1:1 mixes of *R. fascians* D188-5/WT and *R. fascians* D188-5/Δ*lyd* were prepared. The second competition assay used biological replicates of *R. fascians* WT and Δ*lyd* strains (n = 7 each) from separate colonies, against the same D188-5 biological isolate.

A serial dilution of these inocula was prepared in LB (from 10^{-1} to 10^{-8} dilution) and 5 µL spots were plated onto both LB and LB + streptomycin plates. Plasmid-free *R. fascians* D188-5 is streptomycin resistant while the competing WT and Δ*lyd* strains are streptomycin sensitive. These plates were incubated at 30 °C for 1-3 days until individual colonies could be counted. The CFU/mL was calculated for each strain. The CFU/mL of the competing strain (WT or Δ*lyd*) was calculated by subtracting the CFU/mL grown under streptomycin selection (count of only D188-5) from the total CFU/mL grown without selection (count of both strains). The same inoculum was used to infect the seedlings. 4 µL of the inoculum (approximately $2x10^5$ cells) was applied to each seedling. In the first experiment, 8 groups of 7 seedlings were infected per condition. In the second experiment, 7 groups of 8 seedlings per timepoint were infected per condition. The plates were grown under 16/8 hour light/dark at 28 °C. In the first experiment, bacteria were isolated at 7 dpi, whereas in the second experiment, bacteria were isolated at 3, 7 and 14 dpi. For both experiments, the seedlings were aseptically transferred to a 2 mL microcentrifuge tube containing 1 mL sterile 100 mM $MgCl_2$ and vigorously mixed for 1 hour. These were serially diluted in LB (from 10^{-1} to 10^{-8} dilution) and 5 µL spots were plated onto both LB and LB + streptomycin plates. These plates were incubated at 30 °C for 1-3 days until individual colonies could be counted. The CFU/mL were then calculated as described above. The data and statistics were processed in GraphPad Prism, where relative fitness was calculated: ln(test CFU 7dpi/ test CFU 0dpi)/ln(D188-5 CFU 7dpi/D188-5 CFU 0dpi).

SUPPLEMENTARY TABLES

Table S1 Bacterial strains used in this study.

Strain	Genotype/Description	Application	Reference
E. coli DH5α (NEB)	*fhuA2 Δ(argF-lacZ)U169 phoA glnV44 Φ80 Δ(lacZ)M15 gyrA96 recA1 relA1 endA1 thi-1 hsdR17*	Plasmid host for molecular cloning	
M. smegmatis mc^{2}155 (ATCC 700084)	Wild Type	Mycobacterial reporter strain	[41]
R. fascians D188	Wild Type	Lydiamycin producer	[42]
R. fascians D188-5	pFiD188-free. Strep^R	Avirulent strain lacking the pFiD188 plasmid.	[42]
R. fascians D188 Δ*nrp5* (Δ*lyd*)	Disruption in *lydH*	Disrupted lydiamycin production	[21]
M. smegmatis mc^{2}155::pJAM2-*lydA*	Heterologous expression of *lydA* PDF gene	Testing LydA-mediated lydiamycin resistance	This study
M. smegmatis mc^{2}155::pJAM2-*MsPDF*	Heterologous expression of *MsPDF* PDF gene	Testing MsPDF-mediated lydiamycin resistance	This study
M. smegmatis mc^{2}155::pJAM2	*M. smegmatis* strain harbouring empty pJAM2 plasmid	Control strain for testing lydiamycin activity	This study

Table S2 Media used in this study. All percentages are w/v for solid media components.

Medium	Application	Ingredients
Lysogeny Broth (LB)	*E. coli* culture, *R. fascians* culture	1% Bacto-tryptone, 0.5% yeast extract, 1% NaCl, pH 7.0
SOC	*E. coli* transformation recovery medium	2% tryptone, 0.5% yeast extract, 0.058% NaCl, 0.2% $MgCl_2$, 0.25% $MgSO_4$, 0.36% glucose
Middlebrook 7H9 Broth with OADC Enrichment (MOADC liquid)	*M. smegmatis* liquid culture	0.47% Middlebrook 7H9 broth base, 0.2% glycerol, 10% MOADC growth supplement (after autoclaving)
Middlebrook 7H10 Agar with OADC Enrichment (MOADC solid)	*M. smegmatis* solid culture	1.9% Middlebrook 7H10 agar base, 0.5% glycerol, 10% MOADC growth supplement (after autoclaving)
Aurachin production medium (PM)[22]	*R. fascians*, comparative metabolomics	1% starch, 1% glucose, 1% glycerol. 0.25% corn steep powder, 0.5% bacto peptone, 0.2% yeast extract, 0.1% NaCl, pH 7.3
Rhodostreptomycin PM[23]	*R. fascians*, comparative metabolomics	1% starch, 2% glucose, 2.5% soytone. 0.4% dry yeast, 0.1% beef extract, 0.005% K_2HPO_4, 0.2% NaCl, pH 7.0
Minimal media A (MinA)[24]	*R. fascians*, comparative metabolomics	1.05% K_2HPO_4, 0.45% KH_2PO_4, 0.1% $(NH_4)_2SO_4$, 0.05% sodium citrate, 0.025% $MgSO_4$-$7H_2O$, 0.001% thiamine with 20 mM pyruvate and 5 mM histidine
Yeast Extract Broth (YEB)	*R. fascians*, comparative metabolomics	0.5% peptone, 0.1% yeast extract, 0.5% beef extract, 0.5% sucrose, 0.5% $MgSO_4$-$7H_2O$, pH 7.2
Actinonin PM[25]	*R. fascians*, comparative metabolomics	1% glucose, 1% DIFCO soluble starch, 2% corn liquor step, 2% soy flour, 0.25% NH_4Cl, 0.3% NaCl, 0.6% $CaCO_3$, pH 6.2
Matlystatin PM[25]	*R. fascians*, comparative metabolomics	3% glucose, 7% glycerol, 1% bacto peptone, 1% soy flour, 1% corn steep liquor, 0.5% $MgSO_4$, 0.5% NH_4NO_3, 0.5% NaCl
Bottromycin PM[26]	*R. fascians*, comparative metabolomics	1% glucose, 1.5% starch, 0.5% yeast extract, 1% soy flour, 0.5% NaCl, 0.3% CaCO3, pH 7.0
Screening medium 7 (SM7)	*R. fascians*, comparative metabolomics	2.09% MOPS, 1.5% L-proline, 2% glycerol, 0.25% sucrose, 0.15% L-glutamate, 0.05% NaCl, 0.2% K_2HPO_4 with 2 mM $MgSO_4$, 0.2 mM $CaCl_2$, 0.5% trace salts*, pH 6.5
Screening medium 12 (SM12)	*R. fascians*, comparative metabolomics	1% soy flour, 5% glucose, 0.4% peptone, 0.4% beef extract, 0.1% yeast extract, 0.25% NaCl, 0.5% $CaCO_3$, pH 7.6
Soft Nutrient Agar (SNA)	Spot-on-lawn bioassays	0.8% Difco Nutrient Broth, 0.7% agar
½ MS + 3% Sucrose	Tobacco plant growth	0.22% Murashige & Skoog Medium including vitamins, 3% sucrose, 0.8% agar

* Trace salts (per L in distilled water dissolved in order): 1M H_2SO_4 (1 mL), $ZnSO_4.7H_2O$ (0.86 g), $MnSO_4.7H_2O$ (0.223 g), H_3BO_3 (62 mg), $CuSO_4.5H_2O$ (0.125 g), $Na_2MoO_4.2H_2O$ (48 mg) $CoCl_2.6H_2O$ (48 mg) $FeSO_4.7H_2O$ (1.8 g), KI (83 mg)

Table S3 Primers used in this study.

Primer Name	Sequence (5' to 3')	Application	Restriction Site
pJAM LydA For	GATACAGGATCCATGCCTGTCTCTGAACTTCTGC	Amplification of *lydA* for pJAM2-*lydA* construction	BamHI
pJAM LydA Rev	GATACATCTAGACTATCGTGTTGGCCAATCGTTG		XbaI
pJAM MsPDF For	CATGCCCGAGGTAGTTTTCGGATCCATGGCCGTCGTCCCGATCC	Amplification of *M. smegmatis* housekeeping PDF for pJAM2-*MsPDF* construction	Gibson
pJAM MsPDF Rev	AGTGGTGGTGGTGGTGGTGTCTAGATCAGTGCCCGAACGGATCG		Gibson

Table S4 Vectors used in this study.

Vector	Features	Application	Resistance Marker
pJAM2 [33]	Acetamide-inducible promotor. C-terminal His tagging.	Induced expression in *M. smegmatis*	Kanamycin
pJAM2-*lydA*	pJAM2 with *lydA* insert. C-terminal His-tag.	Expression of lydiamycin BGC PDF in *M. smegmatis*	Kanamycin
pJAM2-*MsPDF*	pJAM2 with *MsPDF* (WP_011727226.1) insert. C-terminal His-tag.	Expression of *M. smegmatis* PDF in *M. smegmatis*	Kanamycin

Table S5 Accessions of mutases from EFI-EST analysis associated with the chemophore clade.

Representative mutase accession	Mutase accessions grouped using EFI-EST[a]
NNN35785.1<br>*Streptomyces* sp. S3	
WP_104814753.1<br>*Kitasatospora* sp. MMS16-BH015	
ATJ34001.1<br>*Streptomyces* sp. ATCC 14903	WP_086815435.1<br>*Streptomyces cacaoi* NPDC 016406
	WP_159787793.1<br>*Streptomyces* sp. NHF165
	QNF54062.1<br>*Streptomyces* sp. KY2
	QHF95772.1<br>*Streptomyces* sp. NHF165
WP_210837750.1<br>*Micromonospora* sp. C31	
WP_019634562.1<br>*Actinomadura atramentaria*	
ALC26898.1<br>*Streptomyces* sp. CFMR 7	MBB4761836.1<br>*Actinoplanes digitatis* DSM 43149
	MBA3364174.1<br>MAG[b]: Actinomycetota bacterium
	WP_176725504.1<br>*Streptomyces* sp. DvalAA-19
	GGS27173.1<br>*Streptomyces aureoverticillatus*
	WP_223771548.1<br>*Streptomyces huiliensis*
AET25252.1<br>*Rhodococcus fascians* D188[c]	
AMY56278.1<br>*Rhodococcus fascians* D188[c]	OZE97895.1<br>*Rhodococcus* sp. 15-2388-1-1a
	OZE22244.1<br>*Rhodococcus* sp. 05-2255-1e
	OZF08685.1<br>*Rhodococcus* sp. 14-2686-1-2
	OZF10685.1<br>*Rhodococcus fascians* 14-2632-D2
	OZC51310.1<br>*Rhodococcus* sp. 06-621-2
	OZC60729.1<br>*Rhodococcus* sp. 06-469-3-2
	OZC94541.1<br>*Rhodococcus* sp. 06-412-2C
	OZD10640.1<br>*Rhodococcus* sp. 06-221-2
	OZD31149.1<br>*Rhodococcus* sp. 06-156-3
	OZD86423.1<br>*Rhodococcus* sp. 05-2256-B3
	OZE41003.1<br>*Rhodococcus* sp. 05-2254-4
	OZE57960.1<br>*Rhodococcus* sp. 02-925g
	OZE73377.1<br>*Rhodococcus* sp. 15-649-2-2
	OZE75211.1<br>*Rhodococcus* sp. 15-649-1-2

WP_051852612.1<br>*Streptomyces aureocirculatus*	
MYU63353.1[d]<br>*Streptomyces* sp. SID69	
MYY82164.1<br>*Streptomyces* sp. SID335	MYZ12566.1<br>*Streptomyces* sp. SID337
	NDZ85767.1<br>*Streptomyces* sp. SID10115
	NEA03361.1<br>*Streptomyces* sp.. SID10116
	NEB46693.1<br>*Streptomyces* sp. SID339
QES31823.1<br>*Streptomyces venezuelae* ATCC 14583	MBA2638190.1<br>MAG[b]: Solirubrobacterales bacterium

a. NCBI accessions of the proteins from the EFI-EST analysis that group with the representative accession. See Materials and Methods for details of settings.
b. MAG = metagenome-assembled genome.
c. Two alternative annotations for the D188 mutase. The separate grouping by EFI-EST likely relates to one accession being annotated with a different start codon.
d. Partial sequence of only 71 AA.

Table S6 Predicted functions of the proteins encoded in the *R. fascians* D188 *lyd* BGC.

Gene name	Protein accession number	Pfam domain	Predicted function	Size (AA)	Size (kDa)
lydA	WP_015586162.1	PF01327	Peptide deformylase	188	20.71
lydB	AET25245.1	PF00196	LuxR-type regulator	226	24.86
lydC	WP_015586164.1	PF01040	UbiA family prenyltransferase	282	29.44
lydD	WP_015586165.1	PF03621	MbtH-like	103	11.50
lydE	WP_015586166.1	PF13434	KtzI-like	424	46.95
lydF	WP_202902192.1	PF04299	KtzT-like	218	24.51
lydG	WP_015586168.1	PF01494	Monooxygenase	394	42.68
lydH	WP_015586169.1	Multiple	NRPS	4,925	537.17
lydI	AET25252.1	PF01642	Hexylmalonyl-CoA mutase (α subunit)	461	49.76
lydJ	AET25253.1	PF00550	NRPS	99	11.06
lydK	WP_015586172.1	PF00501	NRPS	527	56.99
lydL	WP_015586173.1	PF00668	NRPS	614	67.49
lydM	WP_015586174.1	PF08240	Crotonyl-CoA carboxylase/reductase (CCR)	440	47.68
lydN	WP_015586175.1	PF02310	Hexylmalonyl-CoA mutase (β subunit)	151	16.37
lydO	WP_015586176.1	PF03308	MeaB-like	251	26.66
lydP	WP_015586177.1	PF13669	Hexylmalonyl-CoA epimerase	144	15.89

Table S7 Lydiamycin A NMR assignment (carbon and proton) and observed 2D cross-peaks (HMBC, COSY, 1,1-ADEQUATE). All NMR recorded in $CDCl_3$. Atom numbering of the revised structure is used (Figure S11).

Position	δ_C ppm	δ_H ppm (multiplicity, *J* in Hz)	HMBC	COSY	1,1-ADEQUATE
**1**	169.8				
**2**	52.7	5.32, m	1, 3, 4	3	1, 3
**3**	24.4	2.38, 1.79, m	2 (weak), 5 (weak)	2, 4	2, 4
**4**	21.5	1.63, 1.59, m	2, 3, 5	3, 5	3, 5
**5**	47.1	3.16, br d (13.4)<br>2.78, qd (13.1, 3.5)	3 (weak), 4 (weak)	4, 5-NH	4
**5-NH**		4.33, br d	4 (weak), 6 (weak)	5	
**6**	174.4				
**7**	50.5	4.86, qd (10.4, 7.3)	6, 8, 9	7-NH, 8	6, 8
**7-NH**		7.31, d (10.4)	6, 7, 9	7	
**8**	18.3	1.46, d (7.3)	6, 7	7	7
**9**	169.0				
**10**	51.4	4.35, m	9, 11, 12	10-NH, 11	9, 11
**10-NH**		7.47, d (8.9)	10, 11, 15	10	
**11**	36.5	1.77, m	10, 12, 13, 14	10	10, 12
**12**	24.6	1.63, m	10, 11, 13, 14	13, 14	11, 13, 14
**13**	22.8	0.94, d (6.6)	11, 12, 14	12	12
**14**	22.3	0.89, d (6.6)	11, 12, 13	12	12
**15**	169.4				
**16**	50.7	5.31, m	15, 17, 18	16-NH, 17	15, 17
**16-NH**		8.31, d (9.9)	16, 18	16	
**17**	68.3	4.79, dd (11.4, 4.3)<br>4.06, d (11.4)	1, 16	16	16
**18**	171.0				
**19**	55.6	4.68, t (5.0)	18, 20, 21, 23	20, 21	18, 20
**20**	19.4	2.38, 1.95, m	18, 19, 21, 22	19, 21, 22	19, 21
**21**	20.7	2.17, 1.95, m	19, 20, 22	19, 20, 22	20, 22
**22**	142.7	6.93, m	20, 21	20, 21	21
**23**	177.4				
**24**	46.0	3.05, m	23, 25, 26, 30	25, 30	23, 25, 30
**25**	31.7	1.75, 1.54, m	23, 24, 26, 27	24, 26	24, 26
**26**	26.9	1.37, 1.26, m	24, 27, 28	25, 27	25, 27
**27**	31.5	1.29, 1.26, m	26, 29	25, 26	26, 28
**28**	22.4	1.28, m	29	29	27, 29
**29**	14.0	0.87, t (6.9)	27, 28		28
**30**	36.6	3.33, dd (17.0, 12.3)<br>2.63, dd (17.0, 5.5)	23, 24, 25, 31	24	24, 31
**31**	176.0				
**31-OH**		11.41, br s			

Table S8 Comparison between published lydiamycin A ^{1}H NMR spectra and data collected in this current study. All NMR recorded in $CDCl_3$.

**Position in original structure (from ref. 43)**	**Position in revised structure[a] (see Fig. S11)**	**Reported ref. 43 δ_H (*J* in Hz)**	**Reported ref. 44 δ_H (*J* in Hz)**	**Observed δ_H (*J* in Hz)**
**1**	**1**			
**2**	**2**	5.30, m	5.28, m	5.32, m
**3**	**3**	2.38, 1.77, m	2.35, 1.77, m	2.38, 1.79, m
**4**	**4**	1.63, 1.59, m	1.60, m	1.63, 1.59, m
**5**	**5**	3.14, m, 2.77, qd (12.0, 3.0)	3.12, br d (13.5), 2.75, br dq (13.5, 3.8)	3.16, br d (13.4) 2.78, qd (13.1, 3.5)
**5-NH**	**5-NH**	4.32, d (12.0)	4.31, br d	4.33, br d
**6**	**6**			
**7**	**7**	4.85, qd (10.5, 7.0)	4.83, qd (10.5, 7.25)	4.86, qd (10.4, 7.3)
**7-NH**	**7-NH**	7.30, d (10.5)	7.27. d (10.5)	7.31, d (10.4)
**8**	**8**	1.44, d (7.0)	1.43, d (7.25)	1.46, d (7.3)
**9**	**9**			
**10**	**10**	4.35, td (8.5, 8.5)	4.33, m	4.35, m
**10-NH**	**10-NH**	7.46, d (8.5)	7.43, d (7.8)	7.47, d (8.9)
**11**	**11**	1.78, m	1.77, m	1.77, m
**12**	**12**	1.65, m	1.63, m	1.63, m
**13**	**13**	0.93, d (6.5)	0.93, d (6.5)	0.94, d (6.6)
**14**	**14**	0.88, d (6.5)	0.87, d (7.8)	0.89,(6.6)
**15**	**15**			
**16**	**16**	5.31, m	5.28, m	5.31, m
**16-NH**	**16-NH**	8.30, d (10.0)	8.25, d (9.85)	8.31, d (9.9)
**17**	**17**	4.77, dd (11.5, 4.5), 4.04, d (11.5)	4.75, dd (11.4, 4.4), 4.03, br d (11.4)	4.79, dd (11.4, 4.3) 4.06, d (11.4)
**18**	**18**			
**19**	**19**	4.67, t (5.0)	4.65, br t (5.7)	4.68, t (5.0)
**20**	**20**	2.35, 1.92, m	2.35, 1.93, m	2.38, 1.95, m
**21**	**21**	2.17, 1.95 m	2.15, 1.92, m	2.17, 1.95, m
**22**	**22**	6.92, m	6.90, br s	6.93, m
**23**	**31**			
**24**	**30**	3.32, dd (17.0, 12.0), 2.62, dd (17.0, 5.5)	3.30, dd (16.8, 12.2), 2.60, dd (16.8, 5.3)	3.33, dd (17.0, 12.3) 2.63, dd (17.0, 5.5)
**25**	**24**	3.04, m	3.02, m	3.05, m
**26**	**25**	1.72, 1.53, m	1.71, 1.52, m	1.75, 1.54, m
**27**	**26**	1.35, 1.25, m	1.35, 1.24, m	1.37, 1.26, m
**28**	**27**	1.29, 1.25, m	1.27-1.26, m	1.29, 1.26, m
**29**	**28**	1.27, m	1.27-1.26, m	1.28, m
**30**	**29**	0.86, t (7.0)	0.85, t (6.5)	0.87, t (6.9)
**31**	**23**			
**31-OH**	**31-OH**	11.37, br s		11.40, br s

[a]Atom numbering that differs with the prior assignment is highlighted in red.

Table S9 Comparison between published lydiamycin A ^{13}C NMR spectra and data collected in this current study. All NMR recorded in $CDCl_3$.

Position in original structure (from ref. [43])	Position in revised structure[a] (see Fig. S11)	Reported ref. [43] δC, multi.	Reported ref. [44] δC, multi.	Observed δC
1	1	169.8, qC	169.1, qC	169.8
2	2	52.7, CH	52.7, CH	52.7
3	3	24.4, CH2	24.4, CH2	24.4
4	4	21.4, CH2	21.4, CH2	21.5
5	5	47.1 CH2	47.1, CH2	47.1
6	6	174.3, qC	174.3, qC	174.4
7	7	50.5, CH	50.5, CH	50.5
8	8	18.3	18.2	18.3
9	9	169.0, qC	169.3, qC	169.0
10	10	51.4, CH	51.4, CH	51.4
11	11	36.4, CH2	36.6, CH2	36.5
12	12	24.6, CH	24.6, CH	24.6
13	13	22.8, CH3	22.7, CH3	22.8
14	14	22.3, CH3	22.2, CH3	22.3
15	15	169.3, qC	169.7, qC	169.4
16	16	50.6, CH	50.7, CH	50.7
17	17	68.3, CH2	68.2, CH2	68.3
18	18	171.0, qC	170.9, qC	171.0
19	19	55.6, CH	55.6, CH	55.6
20	20	19.4, CH2	19.3, CH2	19.4
21	21	20.6, CH2	20.6, CH2	20.7
22	22	142.7, CH	142.7, CH	142.7
23	31	175.9, qC	175.9, qC	176.0
24	30	36.6, CH2	36.5, CH2	36.6
25	24	46.0, CH	46.0, CH	46.0
26	25	31.6, CH2	31.6, CH2	31.7
27	26	26.9, CH2	26.8, CH2	26.9
28	27	31.5, CH2	31.5, CH2	31.5
29	28	22.4, CH2	22.4, CH2	22.4
30	29	14.0, CH3	13.9, CH3	14.0
31	23	177.3, qC	177.3 qC	177.4

[a]Atom numbering that differs with the prior assignment is highlighted in red.

SUPPLEMENTARY FIGURES

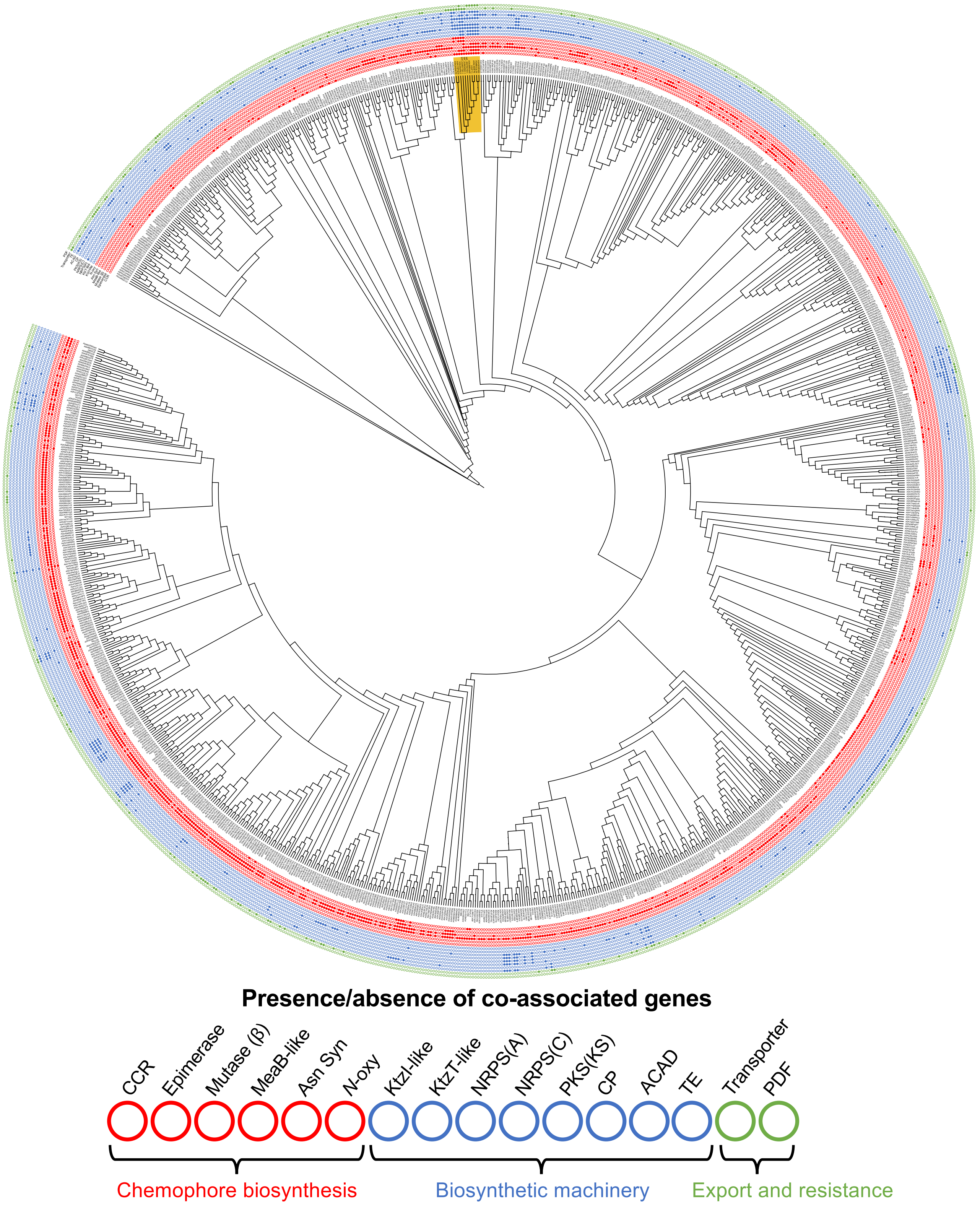

Figure S1 Phylogenetic tree of actinobacterial methylmalonyl-CoA mutases (α subunit). Proteins of <300 AA were pruned from the tree (likely truncated sequences) and branch lengths are not shown for clarity. The tree is annotated with the co-association analysis of pfam domains that are proposed to be involved in HPS analysis or assembly line biosynthesis (NRPS or PKS) and are encoded within 15 genes each side of the mutase gene. A filled red circle indicates the presence of a proposed HPS chemophore gene, a filled blue circle indicates the presence of an NRPS or PKS gene, and a filled green circle indicates an export or putative resistance gene. The 'chemophore clade' is coloured in

orange. Abbreviations are defined in Figure 1B of the main text, plus ACAD (acyl-CoA dehydrogenase; proposed to be involved in the dehydrogenation of the carboxy terminus of matlystatin). An interactive version of the tree is viewable at:
https://itol.embl.de/shared/durSMyJ5S9C2.

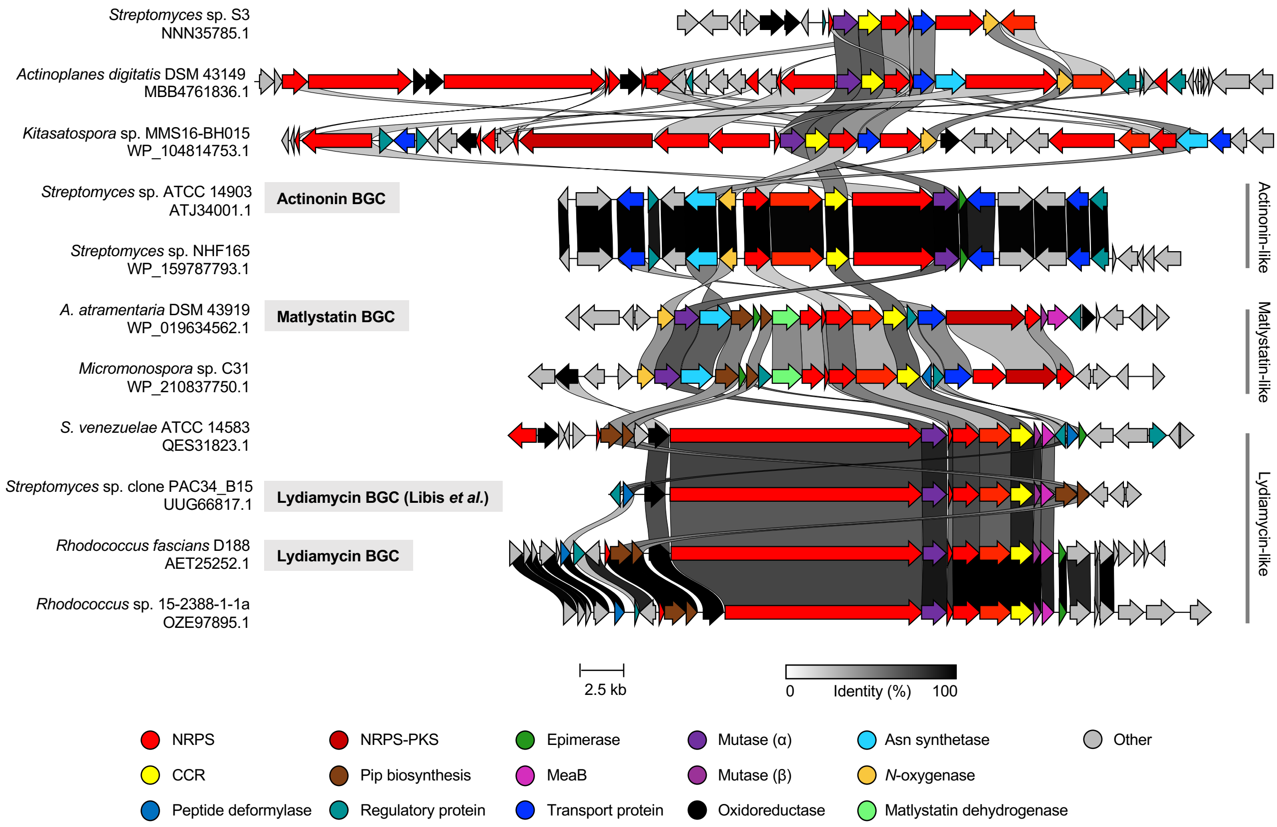

Figure S2 Comparison of mutase-containing BGCs. The accession of the mutase (α subunit) of each BGC is listed under each strain name, while characterised BGCs are highlighted. An example of a *Rhodococcus* BGC with a shorter NRPS than *R. fascians* is shown, along with the lydiamycin BGC previously identified by Libis *et al.* from a *Streptomyces* species[45]. The figure was generated using clinker[9], where shaded links indicate sequence identity of >30% between proteins encoded in each BGC.

A

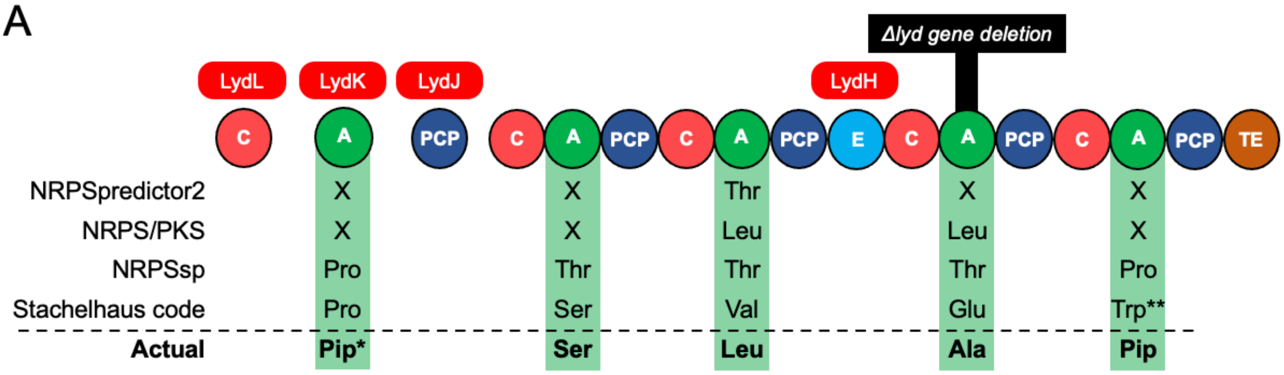

B

Adenylation Domain	Stachelhaus sequence										Nearest Stachelhaus code (% match)	Known Substrate	
LydH M1	D	V	W	S	V	A	M	V	H	K	Ser (90%)	Ser	
LydH M2	D	F	W	T	L	G	A	V	Y	K	Val (70%)	Leu	
LydH M3	D	V	W	T	F	G	S	V	Y	K	Glu (70%)	Ala	
LydH M4	D	V	W	N	F	G	G	F	A	K	N-(1,1-dimethyl-1-allyl)Trp (70%)	Pip	
LydK	D	V	Q	F	N	A	H	M	V	K	Pro (80%)	Pip*	▲
MatJ	D	V	Q	F	N	A	H	M	V	K	Pro (80%)	Pip	▲
MatO	D	A	W	F	L	G	N	I	I	K	Leu (80%)	Leu	
ActH	D	F	W	N	I	G	M	V	F	K	Thr (90%)	Val	
ActE	D	V	Q	F	V	A	H	V	V	K	Pro (90%)	Pro	

Figure S3 The domain specificity of the *R. fascians lyd* NRPS. A. Schematic of NRPS and predicted domain architecture. The predicted adenylation specificity using four different tools is denoted[10–13]. The domain that is disrupted in the Δ*lyd* mutant is highlighted. Abbreviations: A = adenylation, C = condensation, PCP = peptide carrier protein, E = epimerase, TE = thioesterase, Pip = piperazic acid. *Lydiamycin A features a dehydropiperazic acid predicted to be incorporated by LydK but the timing of dehydrogenation is not known. **See panel B for the full Trp-like molecule. B. Stachelhaus specificity sequences of *lyd* adenylation modules identified by antiSMASH 6.2[10]. Adenylation domains from the related actinonin and matlystatin BGCs are included as references. Amino acids are colour-coded using the RasMol Shapely scheme and identical specificity codes are denoted by triangles. Abbreviations: Pip = piperazic acid, M = module.

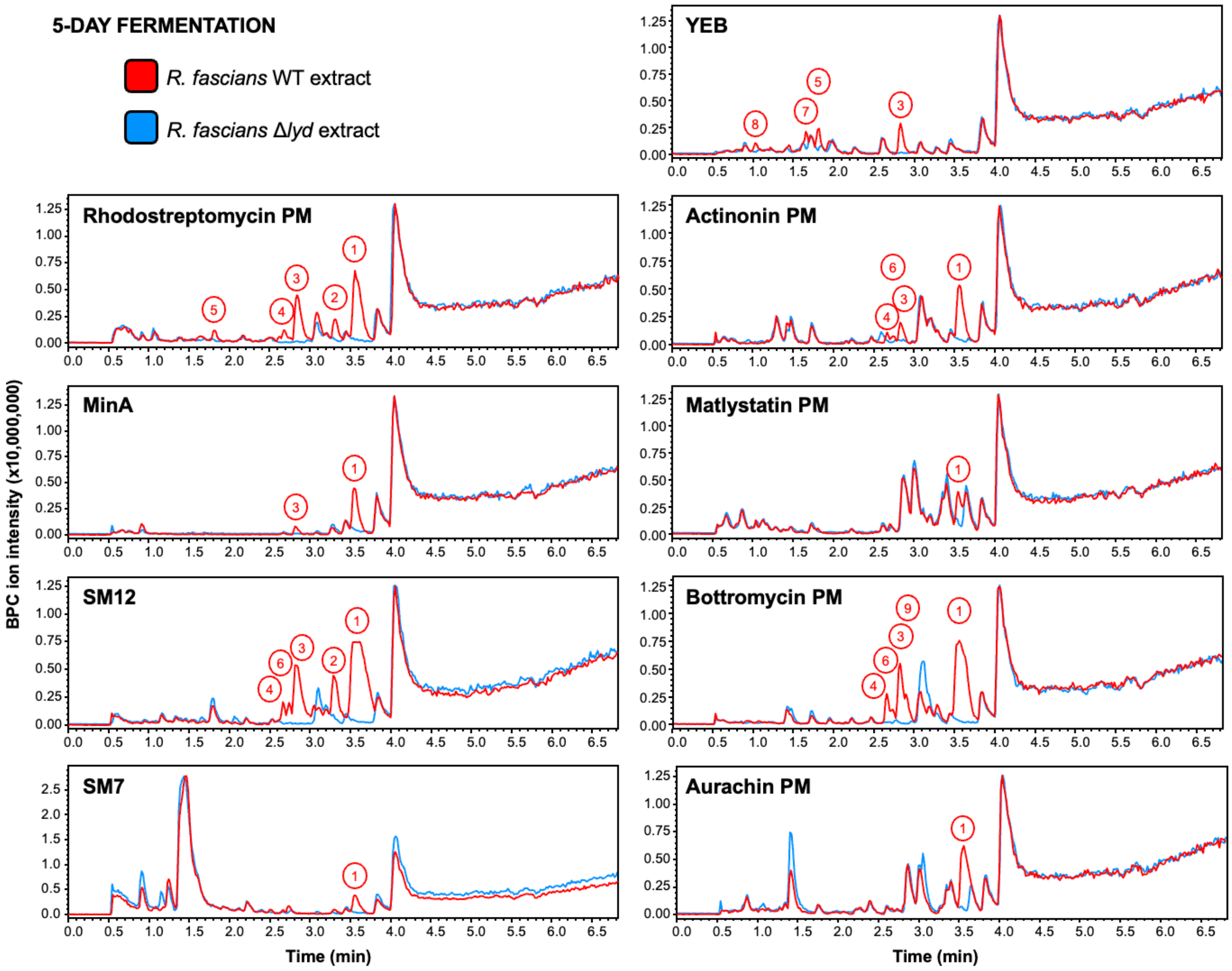

Figure S4 LC-MS chromatograms comparing *R. fascians* WT and Δ*lyd* strains fermented in different media for 5 days. BPC = base peak chromatogram; PM = production medium. Numbers highlight metabolites seen in WT extracts but not in Δ*lyd* and are listed in Figure S6.

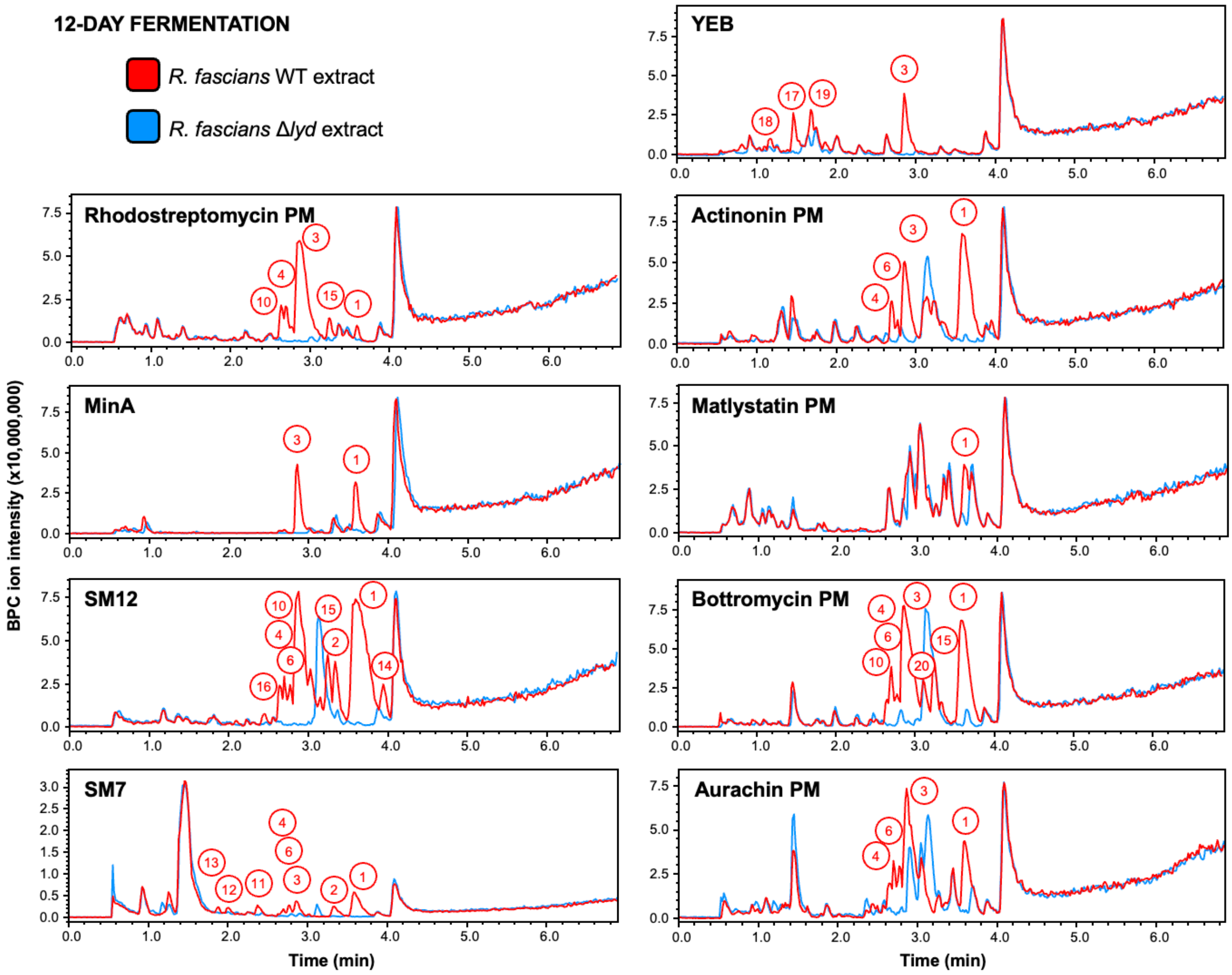

Figure S5 LC-MS chromatograms comparing *R. fascians* WT and Δ*lyd* strains fermented in different media for 12 days. BPC = base peak chromatogram; PM = production medium. Numbers highlight metabolites seen in WT extracts but not in Δ*lyd* and are listed in Figure S6.

A

Compound number	Retention time / min	*m/z*	Compound number	Retention time / min	*m/z*
1	3.55	664.37	11	2.37	580.31
2	3.31	650.35	12	2.00	577.27
3	2.84	682.38	13	1.88	605.26
4	2.67	570.30	14	3.94	700.36
5	1.81	530.30	15	3.25	664.37
6	2.74	689.35	16	2.44	654.35
7	1.66	659.34	17	1.48	553.34
8	1.04	603.31	18	1.16	594.33
9	2.90	703.36	19	1.70	659.34
10	2.64	668.36	20	3.10	655.28

B

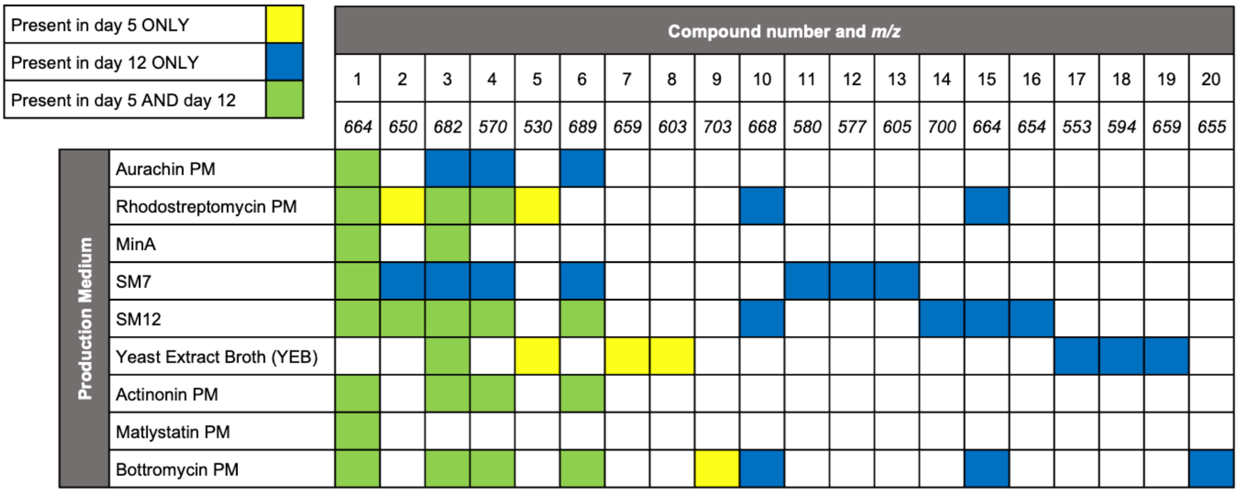

Figure S6 Summary of potential metabolites associated with the *lyd* BGC in different production media. A. List of metabolites observed in Figures S4 and S5. B. Matrix of metabolites versus production medium at different fermentation times.

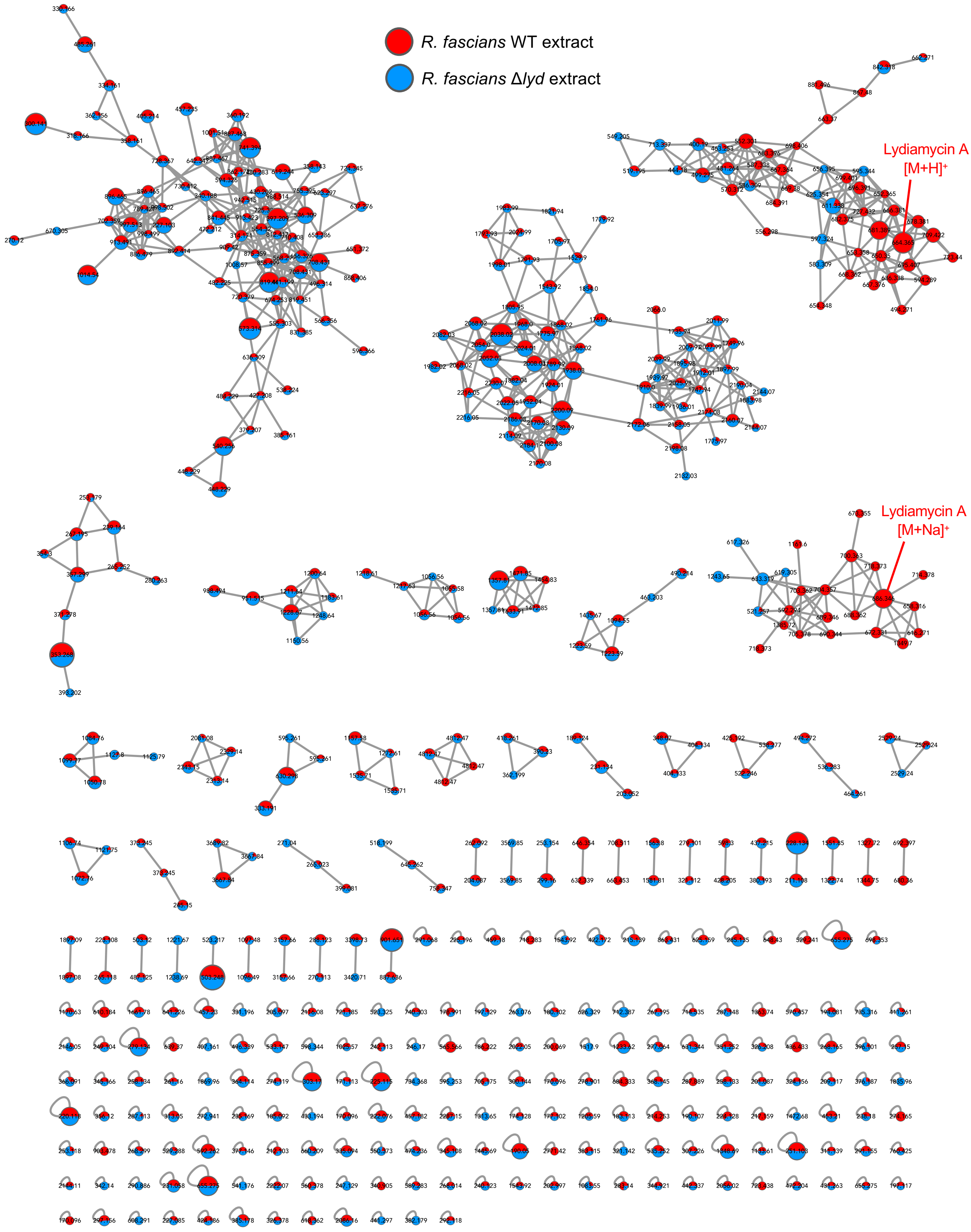

Figure S7 Mass spectral networking of extracts of *R. fascians* WT and Δ*lyd* strains from 5-day SM12 and YEB cultures. Molecular networking was performed with GNPS[28] and visualised using Cytoscape[4]. The node pie charts reflect the relative number of MS/MS spectra for each molecule between *R. fascians* WT and Δ*lyd* samples. Nodes are annotated with parent masses. The diameter of each node is relative to the total number of MS/MS spectra for each molecule: minimum (2 spectra) = 25 node size; maximum (76 spectra) = 75 node size.

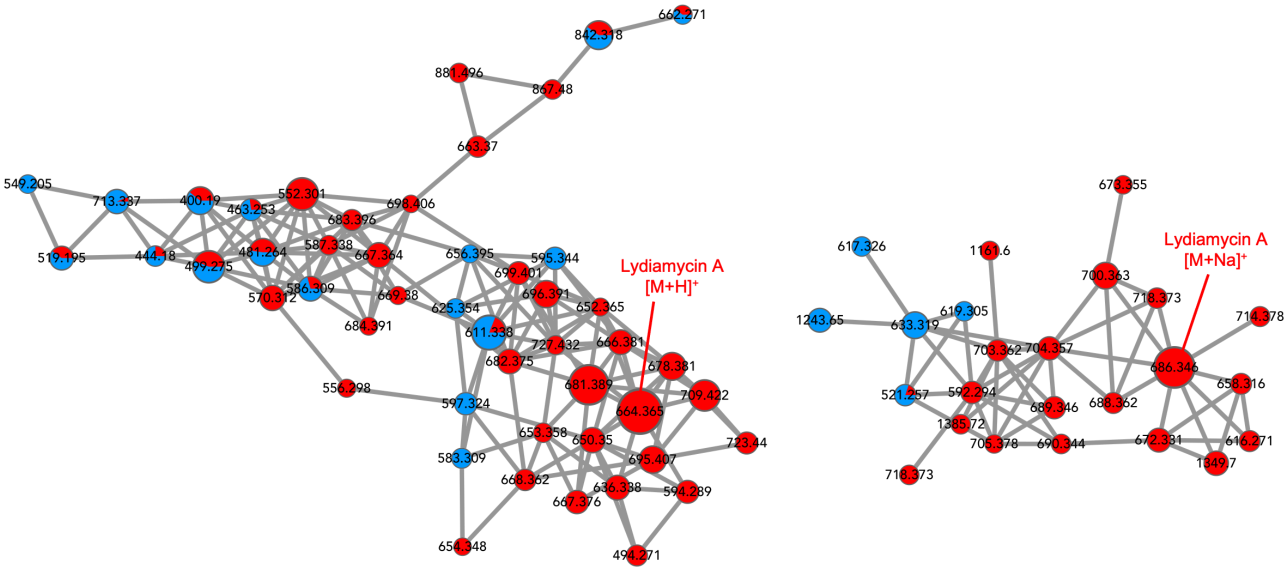

Figure S8 Magnified detail of networks 3 and 4 from Figure S7. The nodes that feature molecules present in Δ*lyd* may reflect low amounts of alternative products of the pathway arising from the in-frame deletion in the *lydH* gene corresponding to the NRPS adenylation domain (see Figure S3).

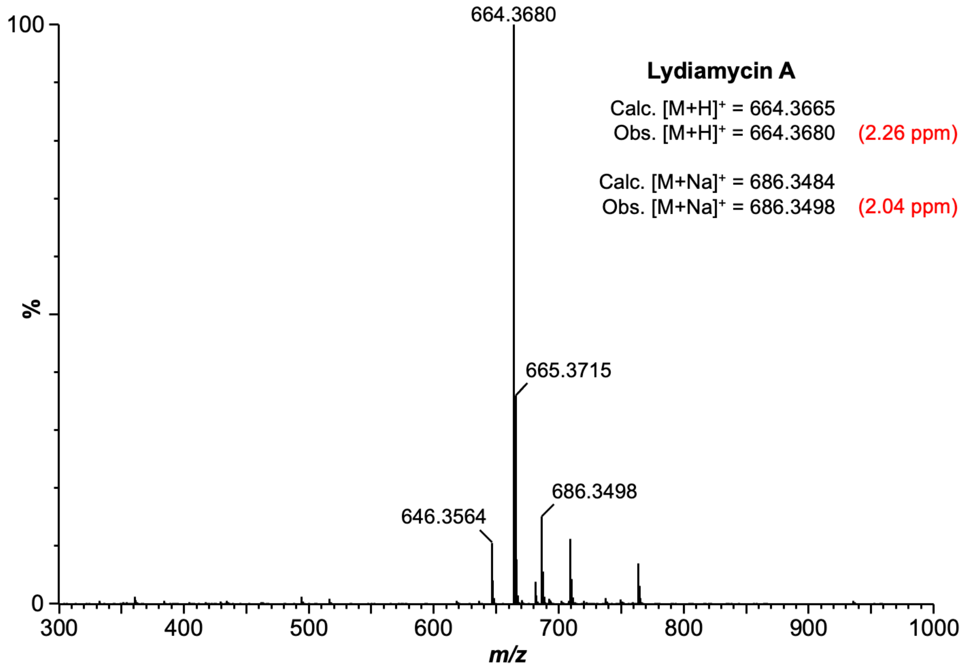

Figure S9 HR-MS spectrum of lydiamycin A, the major *lyd* BGC product. Data acquired on a Synapt G2-Si mass spectrometer.

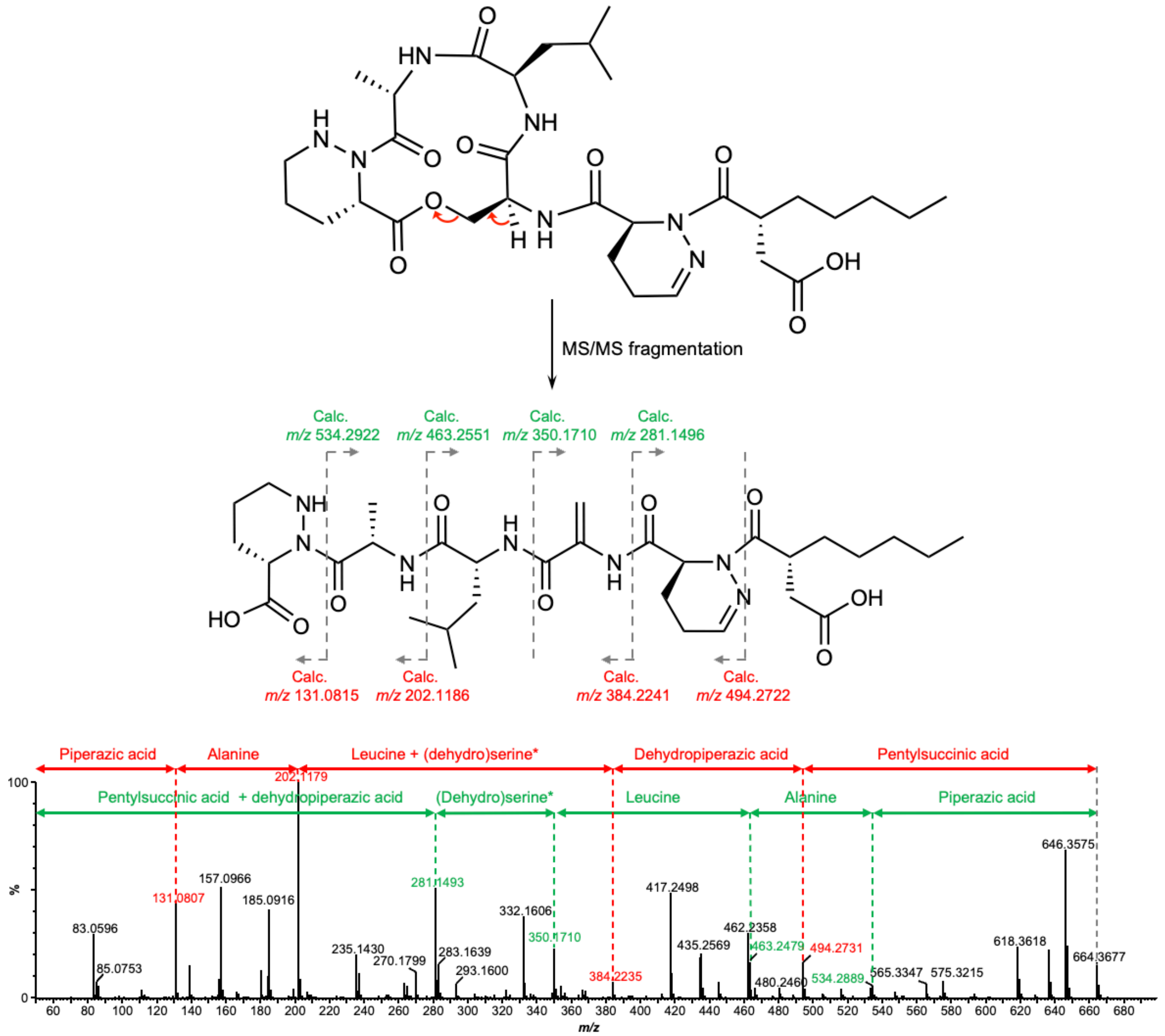

Figure S10 MS/MS fragmentation of lydiamycin A acquired on a Synapt G2-Si mass spectrometer. Observed *y*-ion and *b*-ion masses are annotated at each amide bond. Asterisk denotes that the serine residue is predicted to be dehydrated during molecule linearisation under MS/MS conditions.

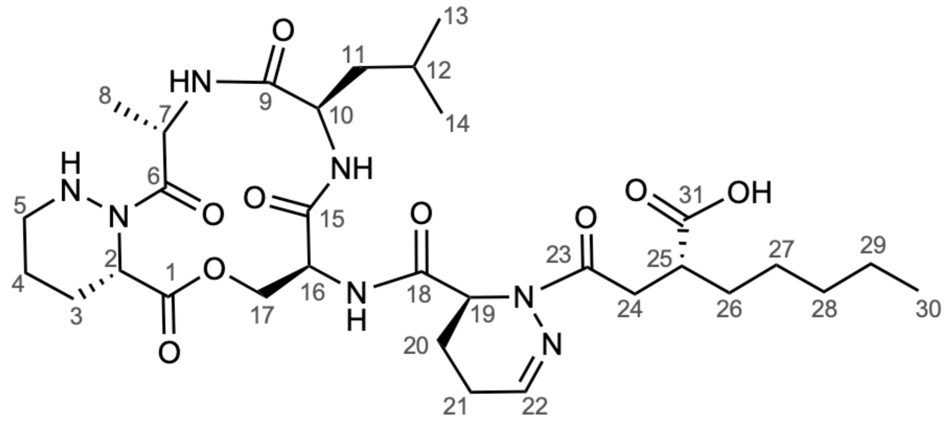

Lydiamycin A structure and numbering from Hwang *et al*.

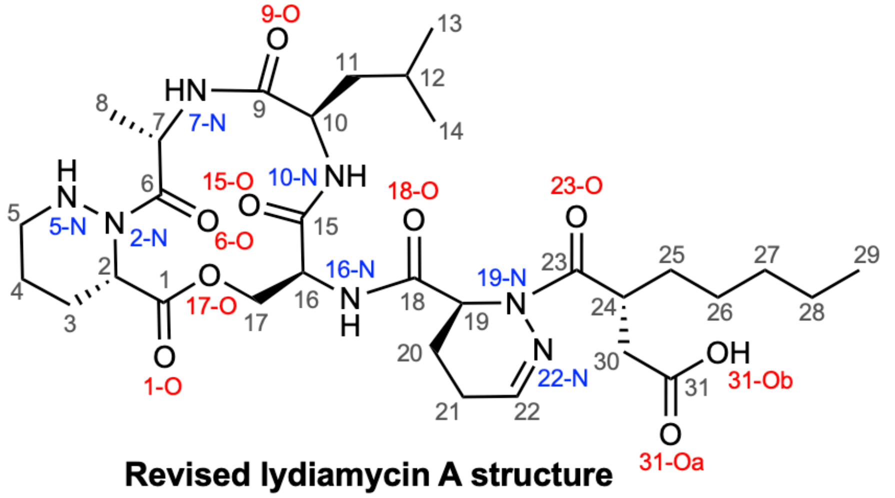

Revised lydiamycin A structure and numbering (this work)

Figure S11 Structures and atom numbering for lydiamycin A from the study of Hwang *et al*.[43] (top) and the revised version from this current work (bottom).

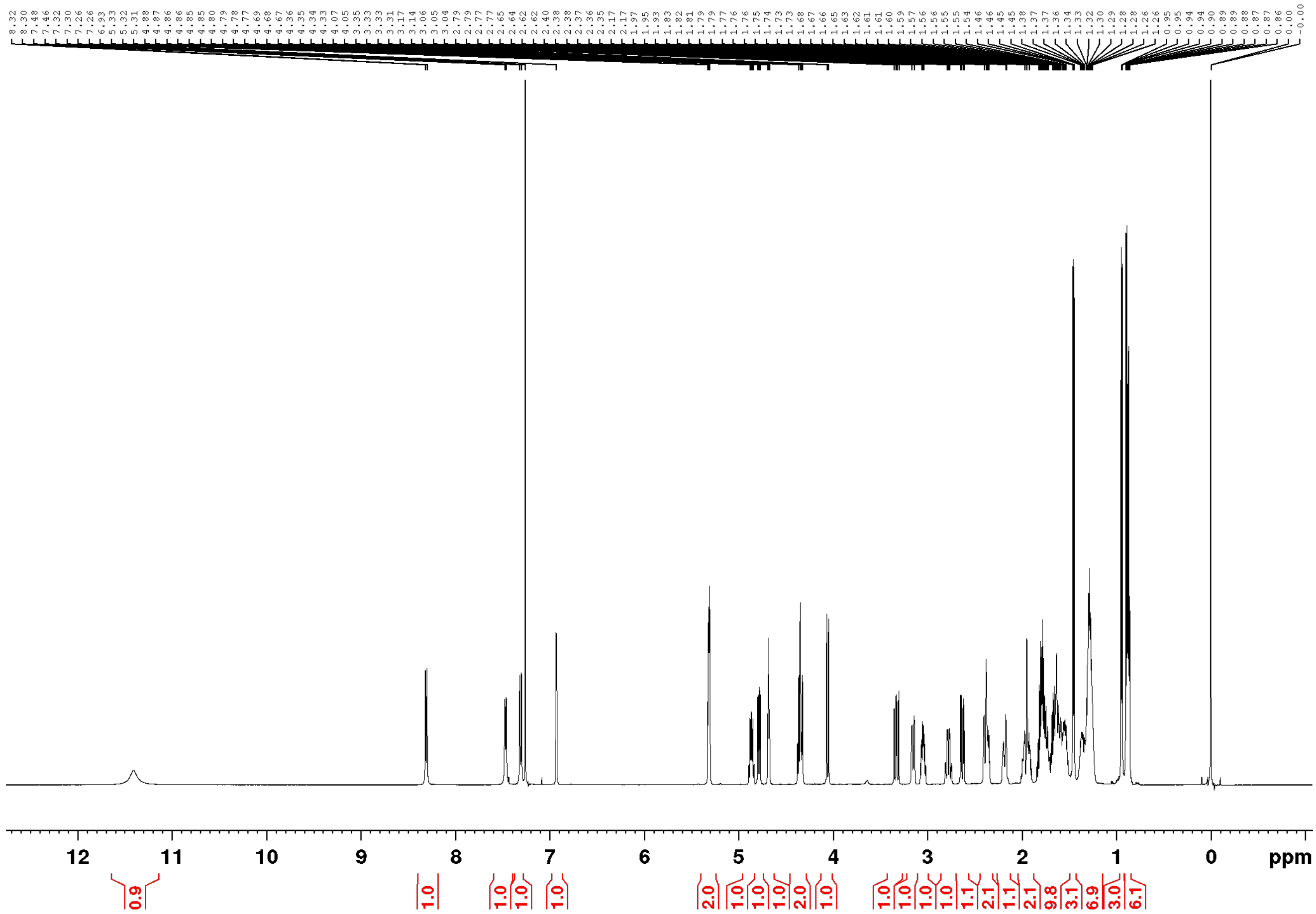

Figure S12 Lydiamycin A ^{1}H NMR spectrum (600 MHz, $CDCl_3$, 298 K).

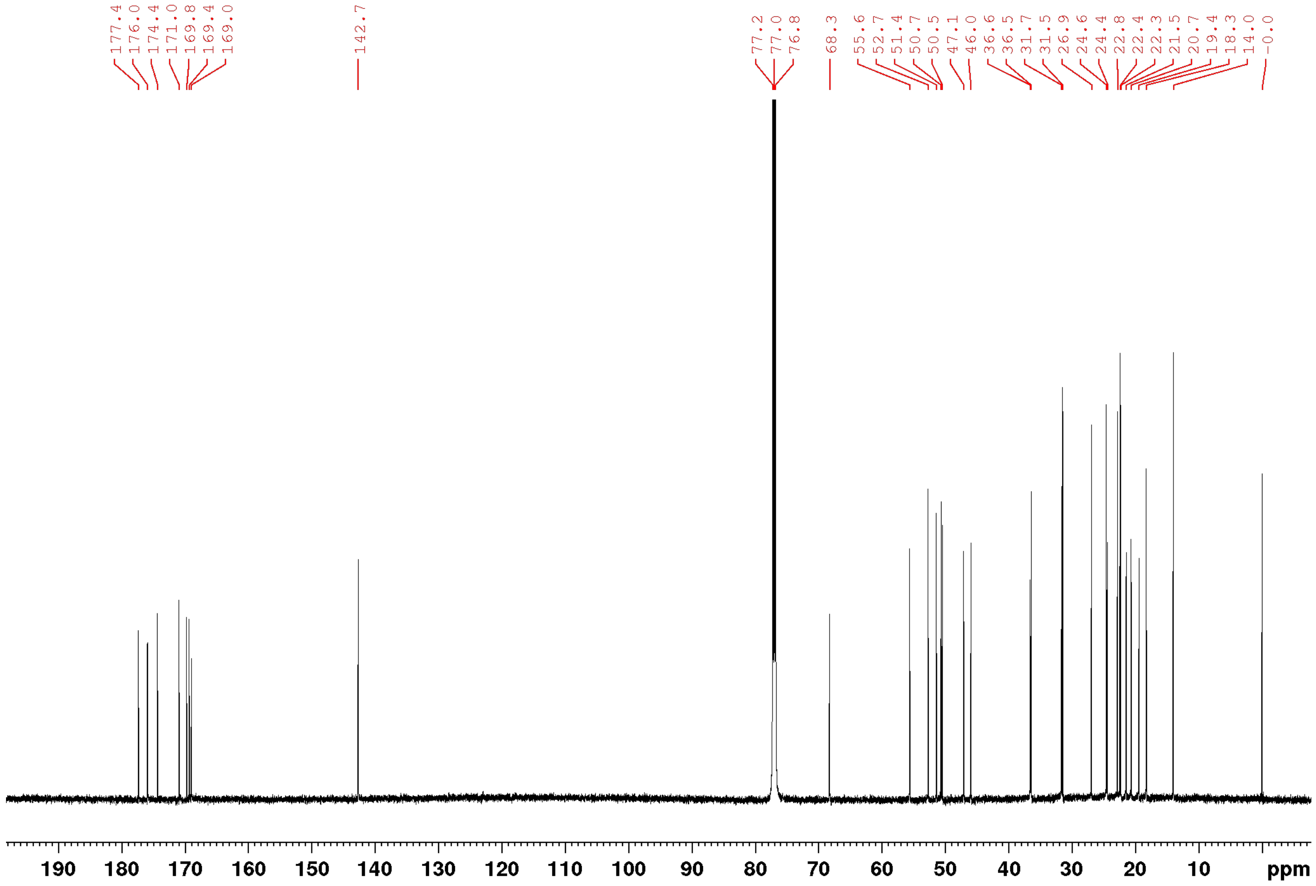

Figure S13 Lydiamycin A ^{13}C NMR spectrum (150 MHz, $CDCl_3$, 298 K).

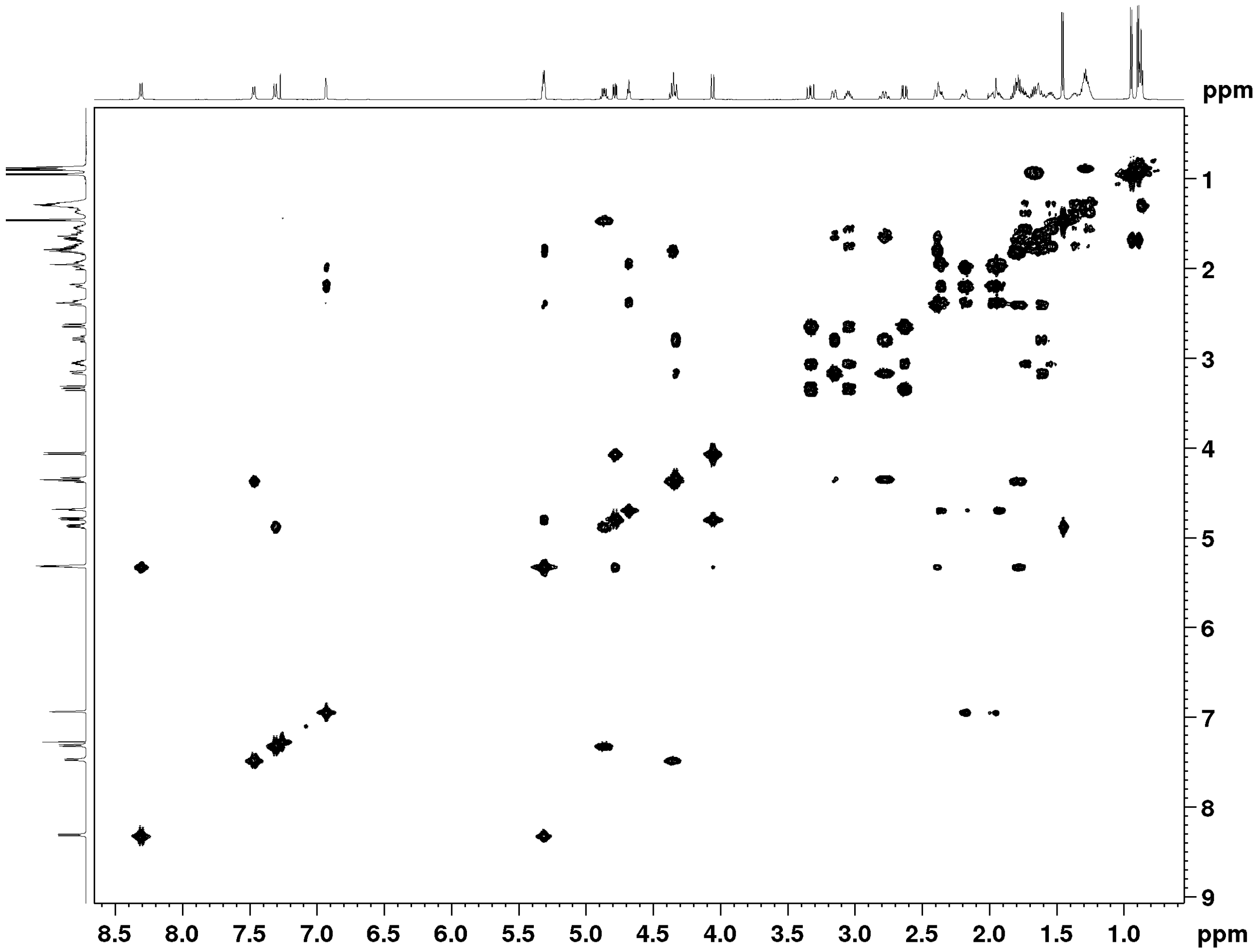

Figure S14 Lydiamycin A COSY NMR spectrum ($CDCl_3$, 298 K).

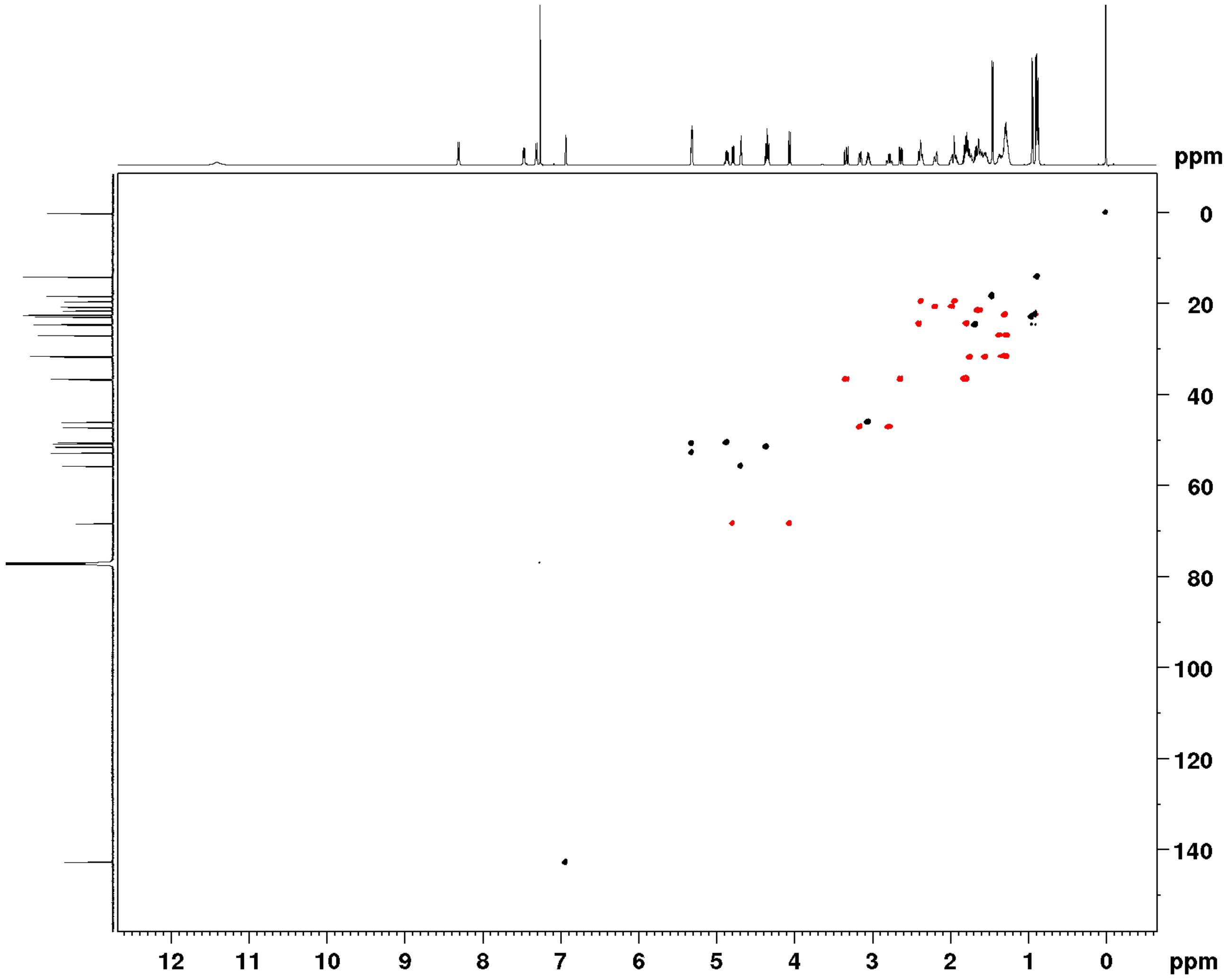

Figure S15 Lydiamycin A multiplicity-edited HSQC NMR spectrum ($CDCl_3$, 298 K).

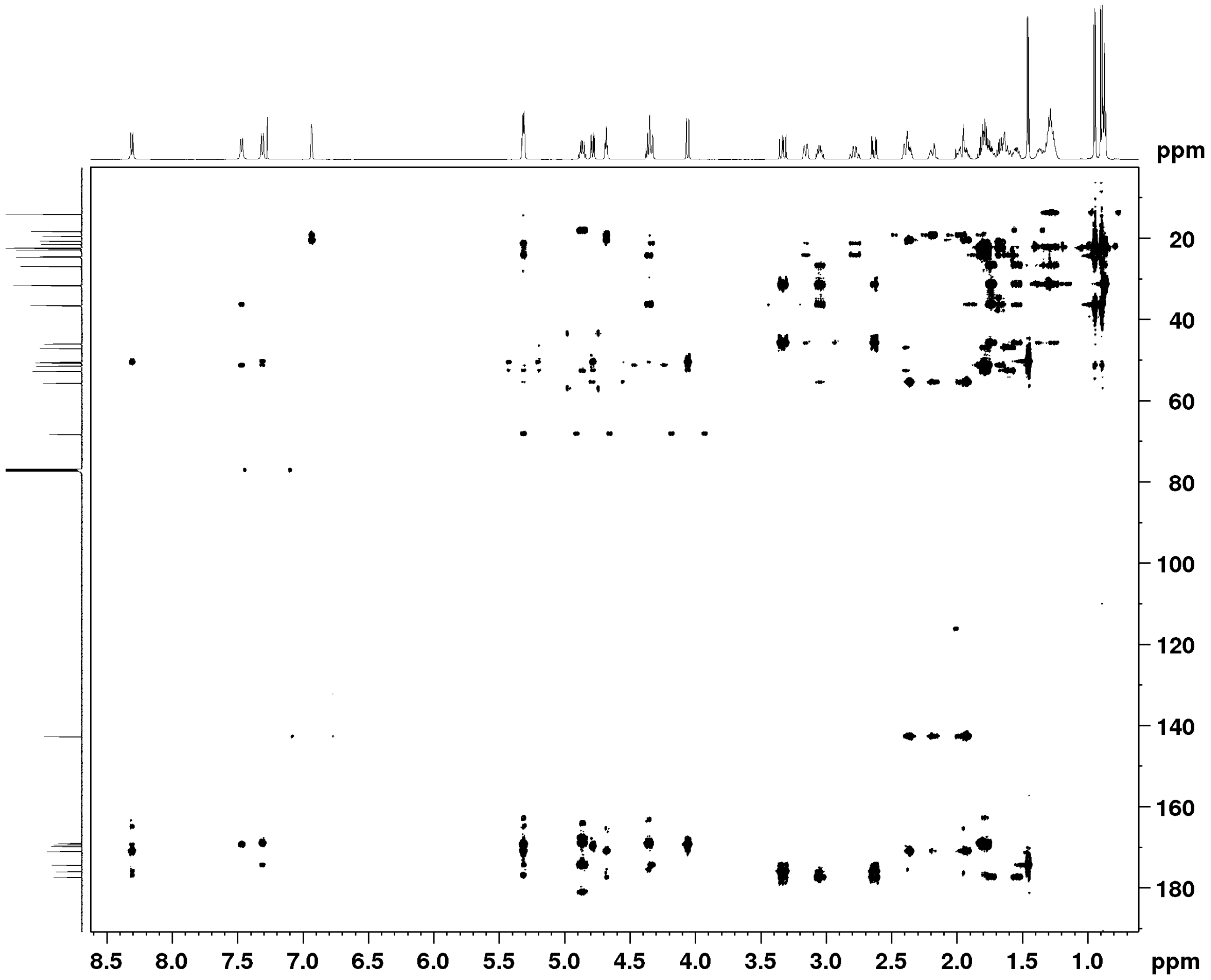

Figure S16 Lydiamycin A ^{1}H-^{13}C HMBC NMR spectrum ($CDCl_3$, 298 K).

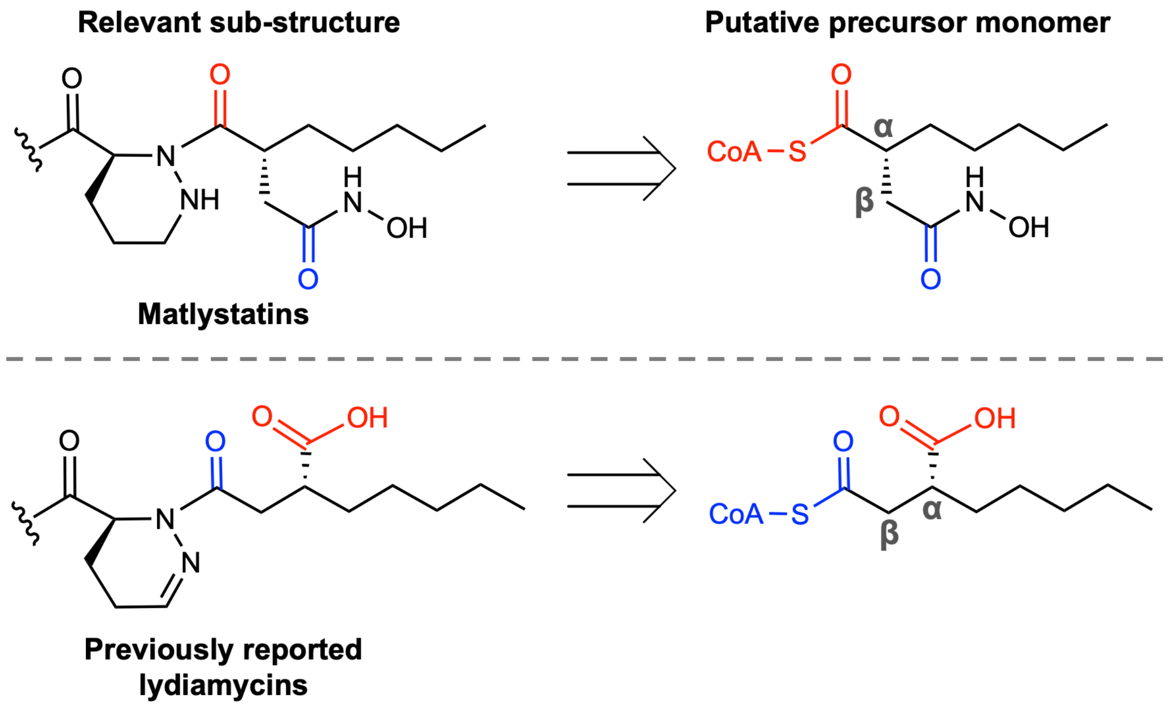

Figure S17 Schematic of potential structural inconsistency of the published lydiamycin A structure when compared to matlystatin. The alpha-proximal carbonyl groups are highlighted in red and the beta-proximal carbonyl groups are highlighted in blue.

A

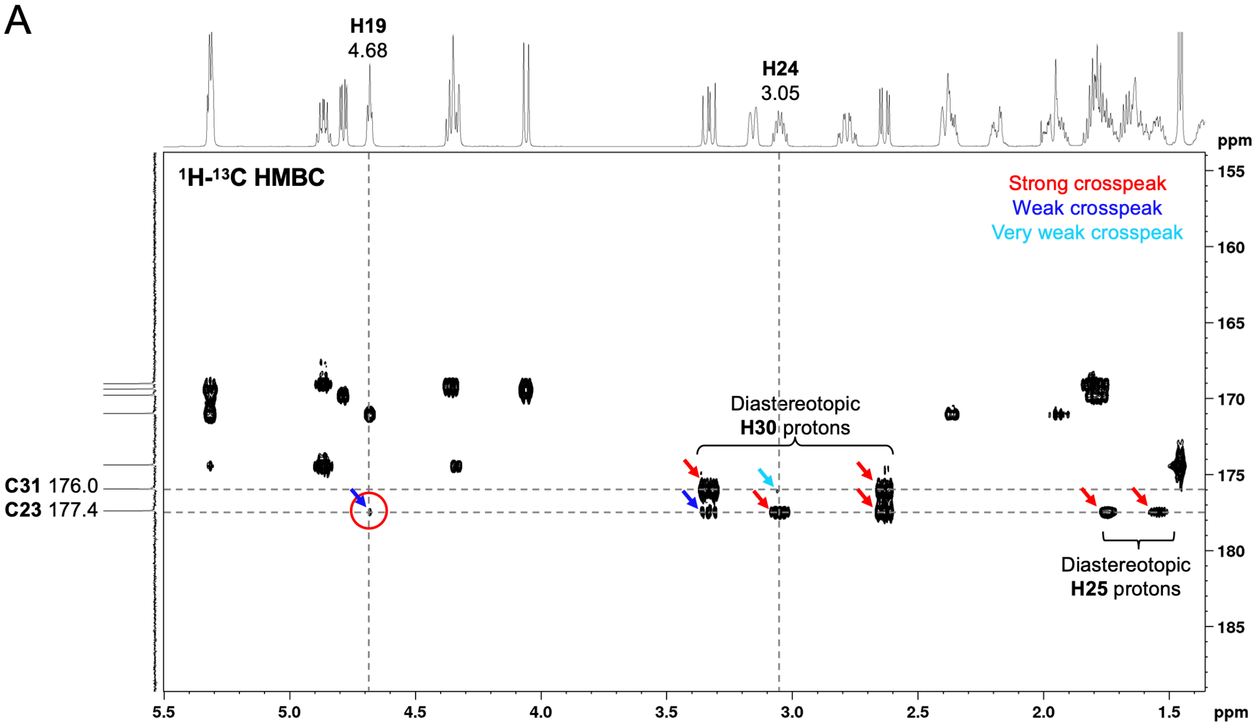

B

	Observed cross-peaks			
**Piperazic-Distal Carbonyl C31** (δ_C 176.0)		Diastereo CH_2 (**H30a**, **H30b**)	CH (**H24**)	
**Piperazic-Proximal Carbonyl C23** (δ_C 177.4)	CH (**H19**)	Diastereo CH_2 (**H30a**, **H30b**)	CH (**H24**)	Diastereo CH_2 (**H25a**, **H25b**)

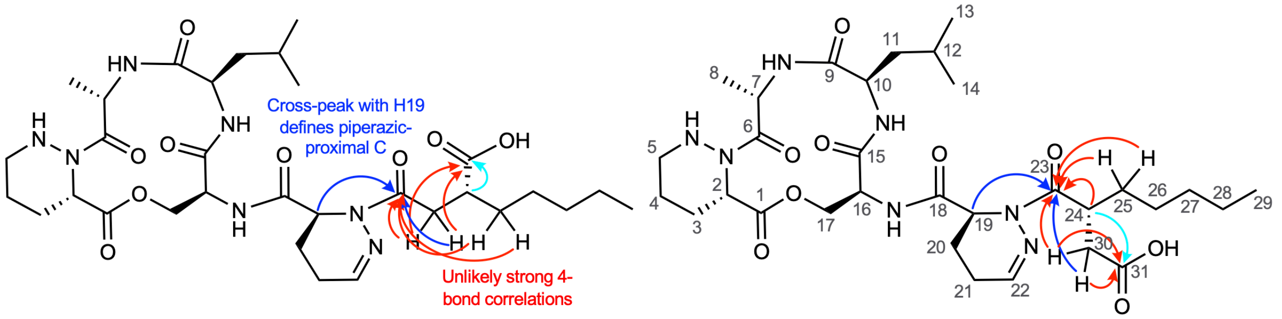

Previously published lydiamycin A structure

Revised lydiamycin A structure

Figure S18 Analysis of pentylsuccinyl region of HMBC spectrum. A. Selected HMBC correlations for C23 and C31. B. The HMBC cross-peak with H19 enabled the identification of the piperazic-proximal and piperazic-distal carbonyls. The remaining correlations provided unlikely strong correlations given the number of connecting bonds in the proposed published lydiamycin A structure, as well as an absence of correlations between H25 protons and the piperazic-distal carbonyl. A revised structure with an actinonin/matlystatin-like pentylsuccinyl moiety provides correlations that are consistent with the number of bonds between atoms.

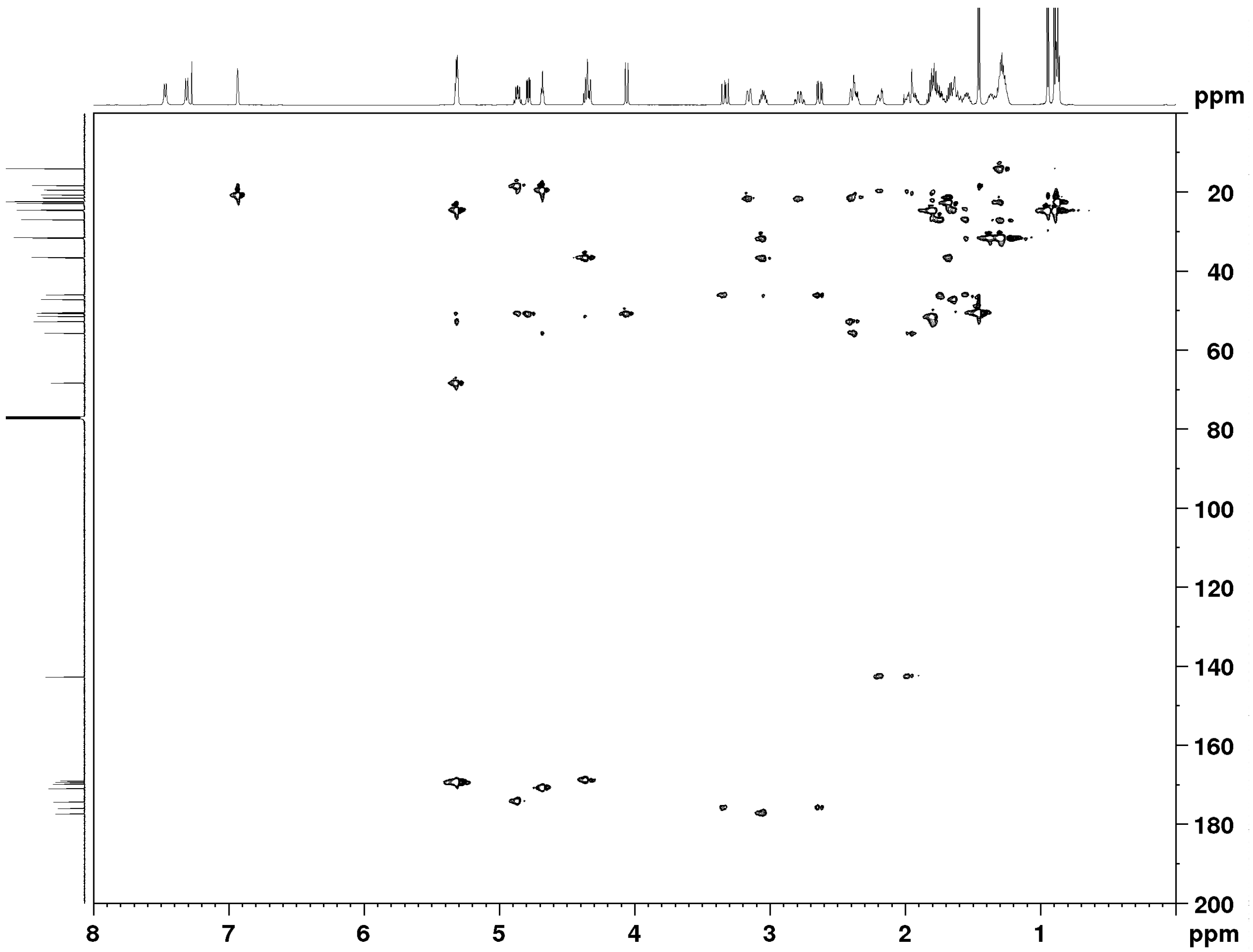

Figure S19 Lydiamycin A 1,1-ADEQUATE NMR spectrum ($CDCl_3$, 298 K).

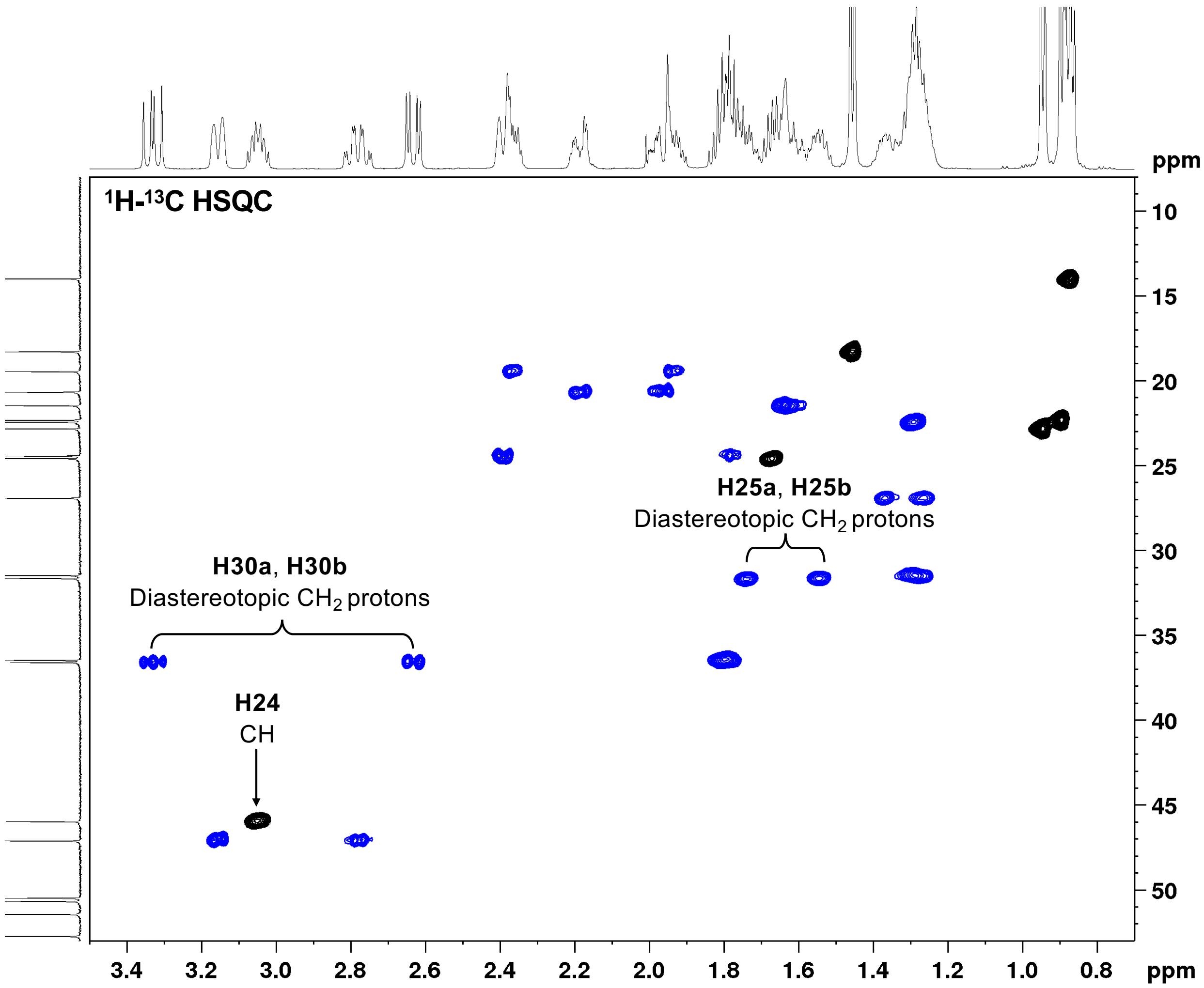

Figure S20 Region within a multiplicity-edited HSQC NMR spectrum of lydiamycin A. This confirms that the peak at 3.05 ppm (observed in the HMBC data) is from a single proton, which was identified as H24.

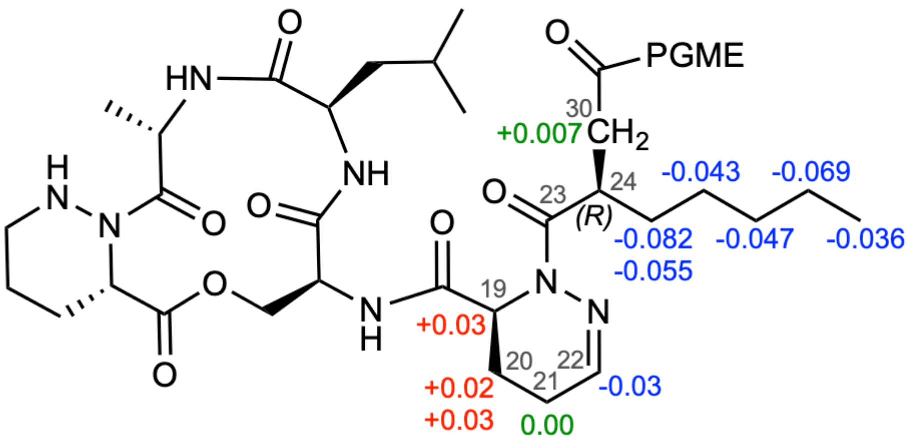

Proton	Lydiamycin δ_H	*R*-PGME product δ_H	*S*-PGME product δ_H	$\Delta\delta=\delta_{(R)}-\delta_{(S)}$
**19**	4.95	4.96	4.93	+0.03
**20a**	2.24	2.23	2.21	+0.02
**20b**	1.77	1.77	1.74	+0.03
**21a**	2.15	Insufficient data	Insufficient data	Insufficient data
**21b**	1.84	1.84	1.84	0.00
**22**	6.91	6.86	6.89	-0.03

Figure S21 Reanalysis of the previously published phenylglycine methyl ester (PGME) derivatisation work published by Hwang and co-workers[43]. The modified Mosher method described by Yabuuchi and Kusumi was applied for using PGME derivatives to determine the absolute configuration of β,β-disubstituted propionic acids[46]. This method involves calculating $\Delta\delta=\delta_{(R)}-\delta_{(S)}$, which meant using the published $\Delta\delta_{S-R}$ (ref. [43]) and inverting the sign of the value. The calculations were also extended to use the published ^{1}H NMR for the PGME derivatives to calculate the $\Delta\delta_{R-S}$ values for the dehydropiperazic acid protons 19-22. When the revised lydiamycin A structure is represented with the CH_2CO-PGME group with a wedge bond, the protons with $\Delta\delta_{R-S}<0$ are to the right and $\Delta\delta_{R-S}>0$ are to the left of this group. These data are consistent with absolute stereochemistry at C24 of *R*. We note that the PGME plane seems to pass through C21, which has a $\Delta\delta_{R-S}$ of 0.00. Furthermore, we note that the published Δδ value for what we have annotated as C30 is a very small magnitude (+0.007), which is expected as it is also in the PGME plane.

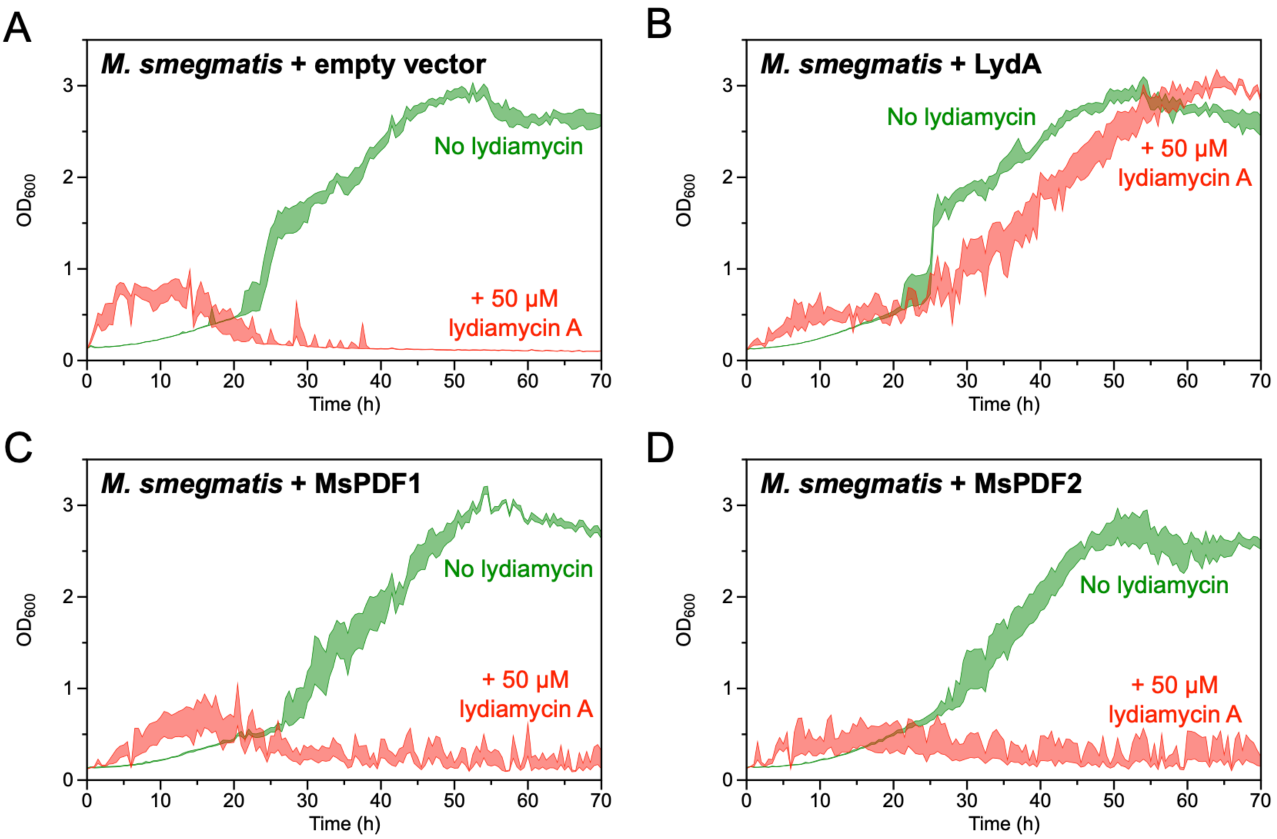

Figure S22 Assessment of lydiamycin A sensitivity for *M. smegmatis* mc^{2}155 overexpressing its native housekeeping PDF gene (*MsPDF*) compared to LydA. The standard error of the mean is illustrated by the shaded envelope for each strain in the absence or presence 50 μM lydiamycin A (n=6 for each condition). The fluctuations in the curves are a consequence of the non-disperse growth of *M. smegmatis*. A. *M. smegmatis* with empty pJAM2 vector. B. *M. smegmatis* with pJAM2-*lydA*. C and D. Two independent transformants of *M. smegmatis* with pJAM2-*MsPDF*.

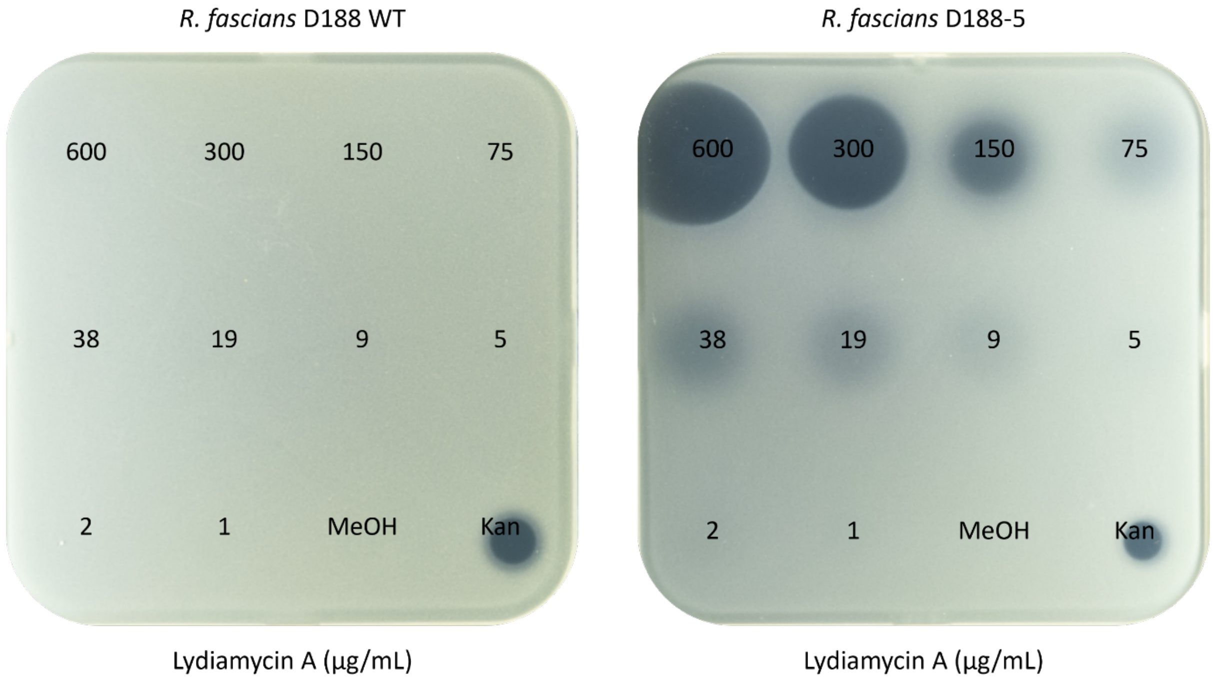

Figure S23 Spot-on-lawn assays of lydiamycin A (600 to 1 µg/mL) against *R. fascians* WT (left) and plasmid-free *R. fascians* D188-5 (right). Kan = 50 µg/mL kanamycin positive control.

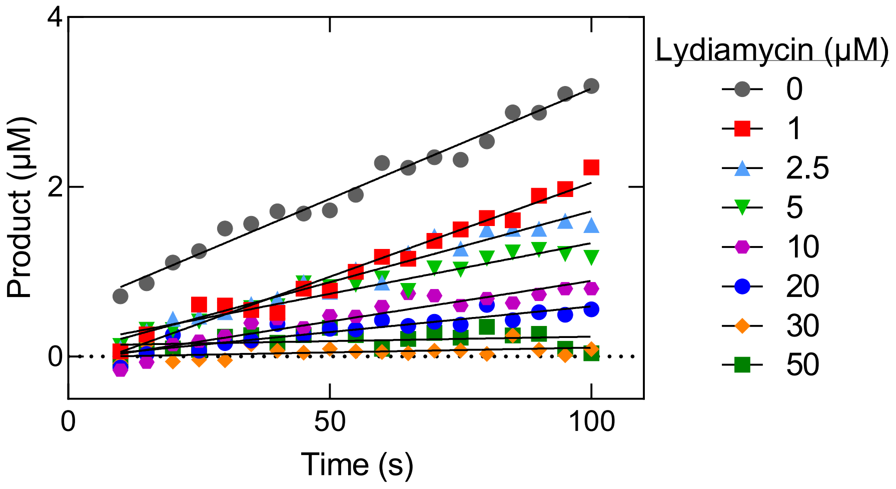

Figure S24 Examples of the deformylation time courses. fML-pNA (20 µM) was deformylated by *E. coli* PDF (10 nM) in the presence of increasing lydiamycin A concentrations. Lines represent the linear regression used to determine the initial velocity shown in Figure 4.

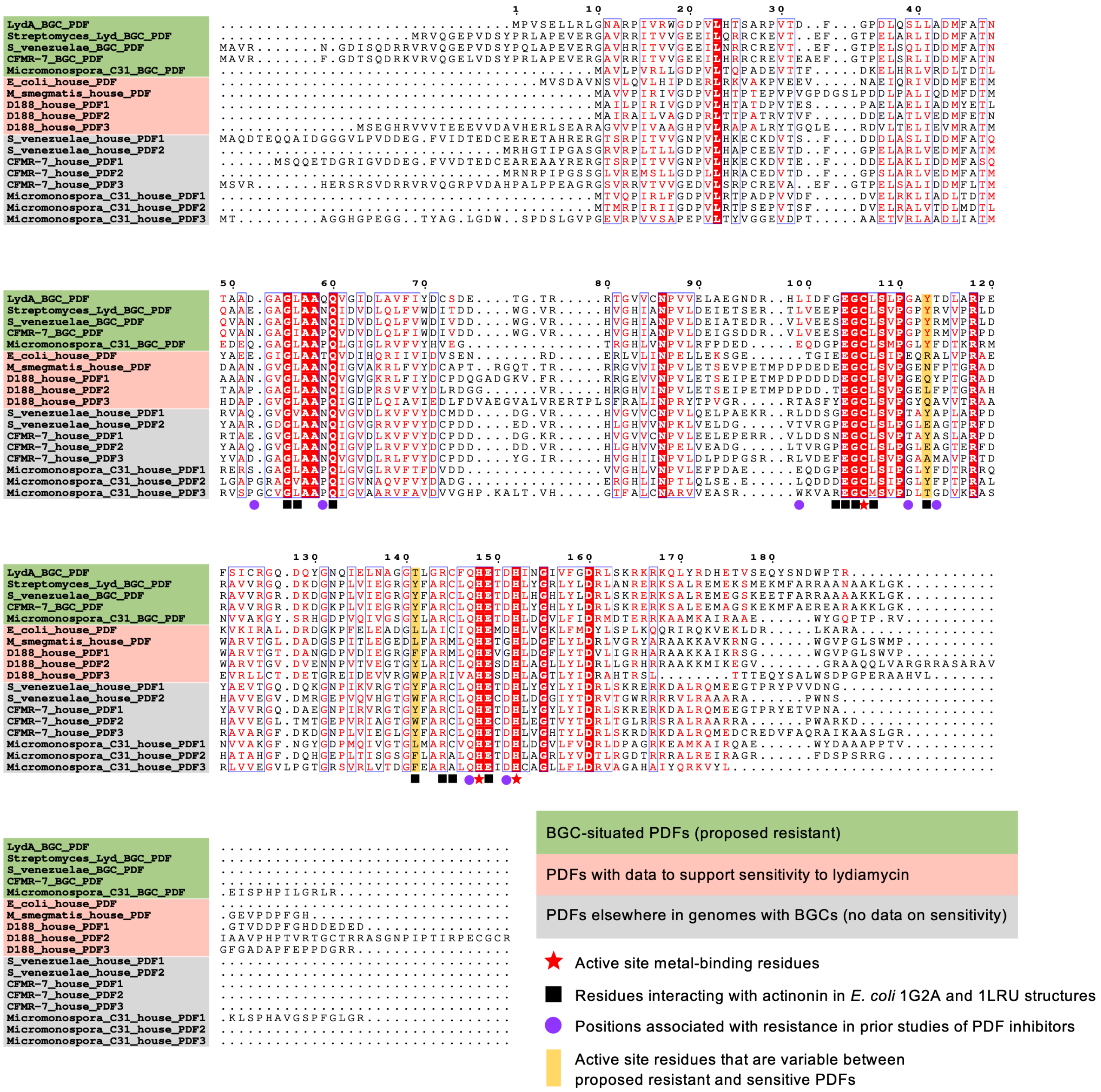

Figure S25 Alignment of PDFs highlighting key sequence features. The aligned sequences are ordered into three groups: (i) PDFs encoded in BGCs predicted or known to make PDF inhibitors (green); (ii) core "housekeeping" PDFs with evidence of sensitivity towards lydiamycin in this study (red); (iii) core PDFs from genomes that also encode BGCs containing PDFs (grey, unknown sensitivity to PDF inhibitors). Note that the number of core PDFs can vary from strain to strain. Sequences were aligned using Clustal Omega[14] and visualised using Espript 3.0[15] with colour-coding according to a Risler matrix with a 0.7 global score cut-off. Strictly conserved residues are shown as white characters on a red background. If the global score for a given position is over 0.7, the residues feature a blue box, while residues letters are coloured red if they have a similarity score over 0.7 within the groups defined above. Metal binding residues are starred, while positions identified as directly interacting with actinonin in prior studies of *E. coli* PDF[19,47] (PDB 1G2A and 1LRU) are denoted by black squares. Sites associated with resistance towards PDF inhibitors in prior studies[48,49] are denoted by purple circles (note: LydA position 99 corresponds to position 123 in *Streptococcus pneumoniae*, where a A123D mutation is associated with actinonin resistance, so the Leu in LydA position is not known to be associated with resistance). Accessions for each PDF are listed in Materials and Methods.

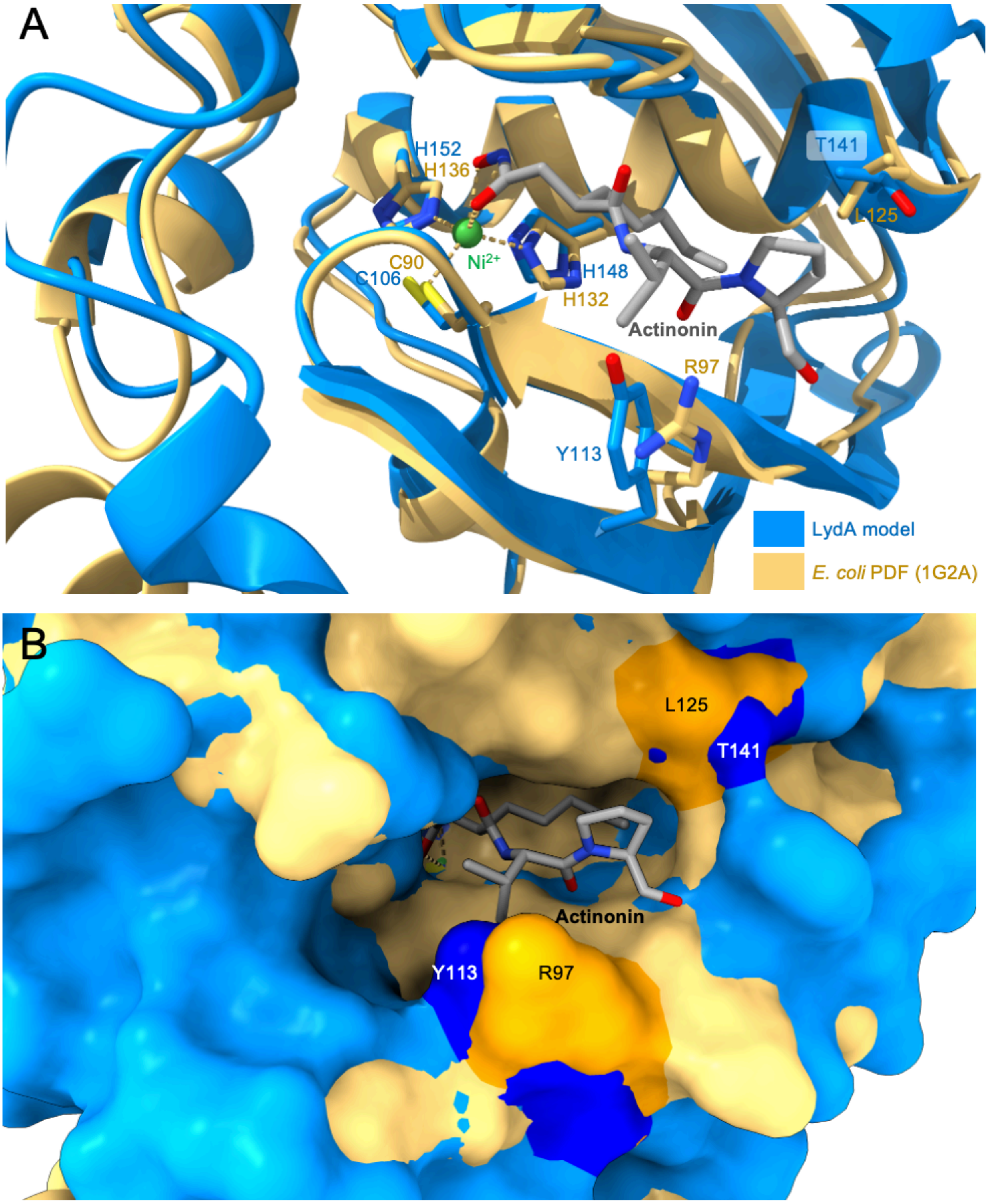

Figure S26 Structural model of LydA PDF generated with ColabFold v1.2[16]. A. Structural alignment of LydA with *E. coli* PDF bound to actinonin[19] (PDB 1G2A). Metal-binding residues are shown, along with residues that differ between resistant and sensitive PDFs (see Figure S25). LydA is blue and *E. coli* PDF is yellow. B. Surfaces of the aligned PDFs with the differential residues highlighted. Note the close proximity of Y113/R97 to the valine side chain of actinonin. Structures were visualised using ChimeraX 1.5[20].

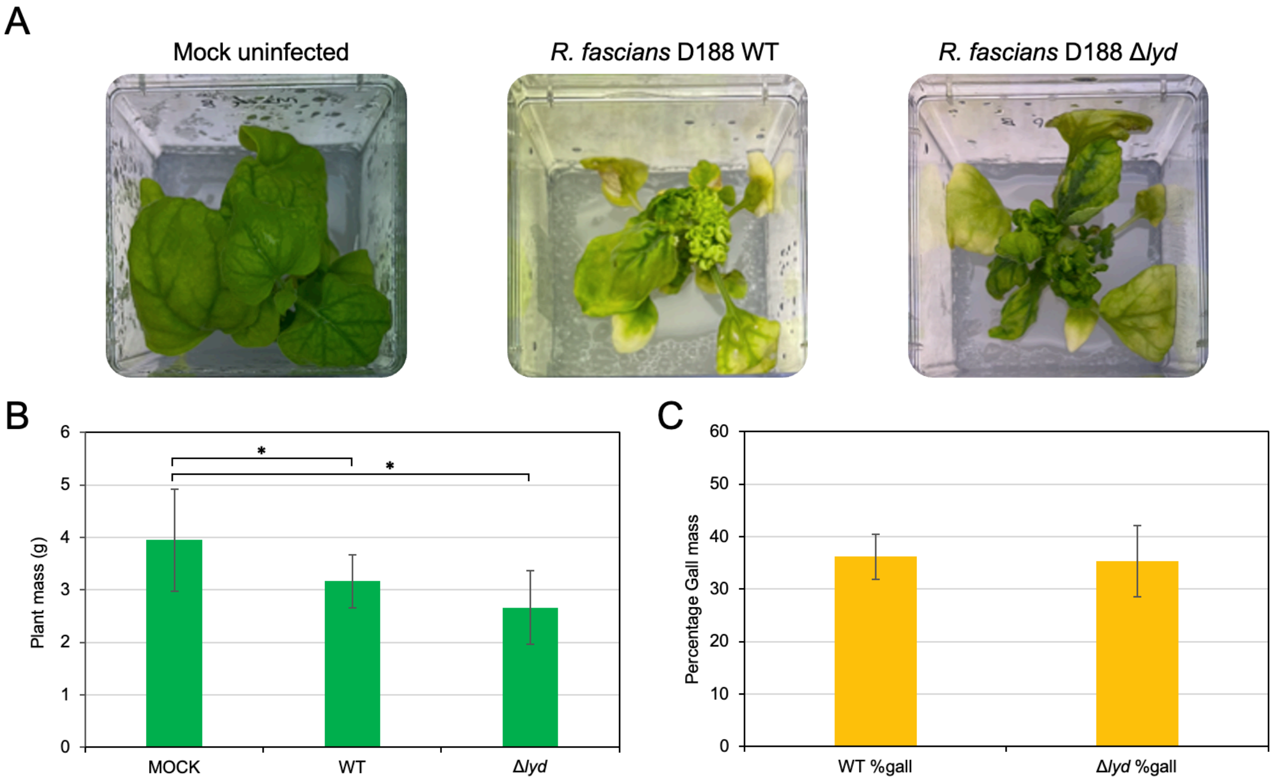

Figure S27 Infection assays with whole *Nicotiana benthamiana* plants (n = 9 plants each). A. Representative images of infected *N. benthamiana* plants. B. Average fresh plant mass (g +/- standard deviation) of plants 4 weeks post infection with either mock solution (100 mM $MgCl_2$), *R. fascians* D188 WT or *R. fascians* D188 Δ*lyd*. The masses of WT infected and Δ*lyd* infected plants were statistically lower than the masses of uninfected plants (Student's *t*-test: p=0.048, 0.024, respectively). There was no significant difference in plant mass between WT infected and Δ*lyd* infected plants. C. Average fresh gall mass in respect to whole plant mass for *R. fascians* D188 WT or *R. fascians* D188 Δ*lyd*.

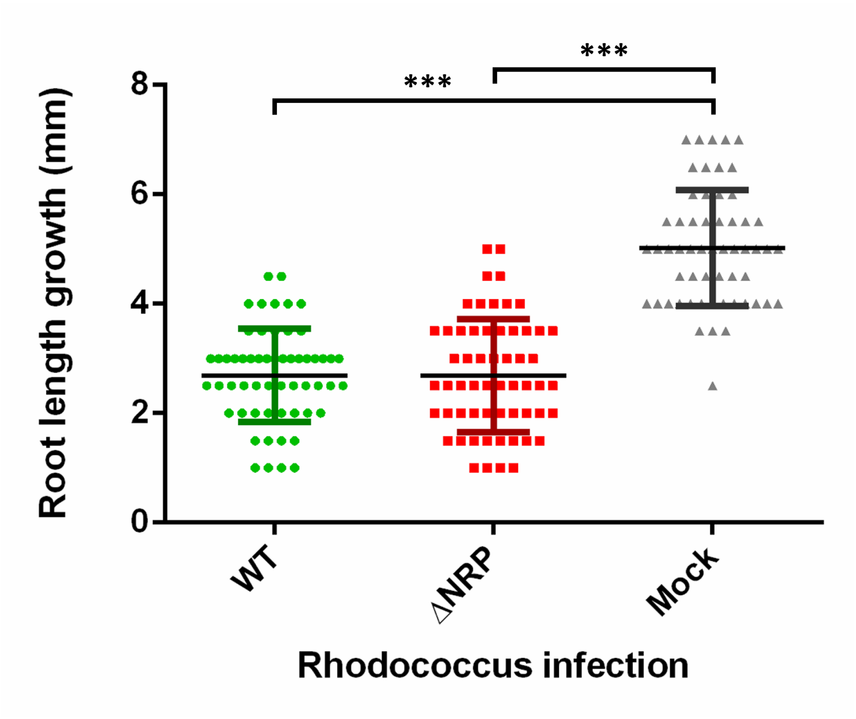

Figure S28 *N. benthamiana* root length assay with either mock solution (100 mM $MgCl_2$), *R. fascians* D188 WT or *R. fascians* D188 Δ*lyd* (ΔNRP). 60 seedlings were used per condition and root growth was assessed after 7 days. *** = Student's *t*-test significance where p<0.00005.

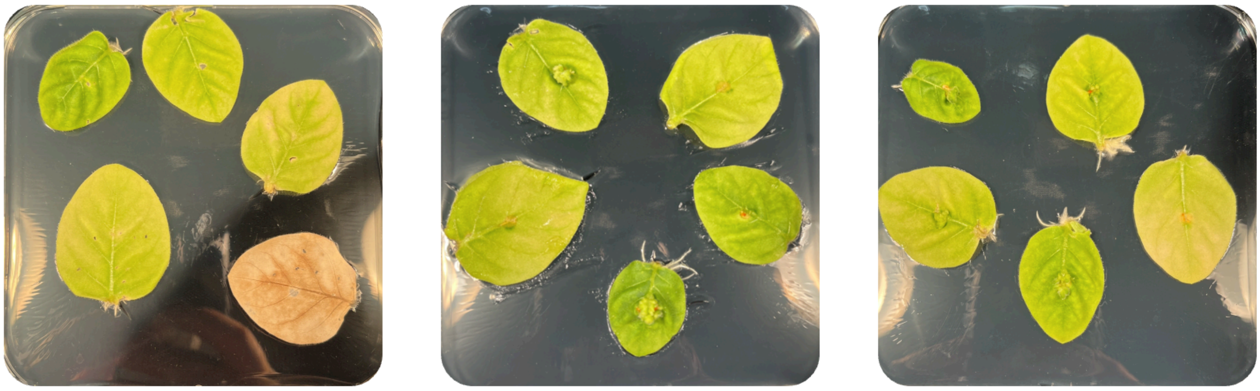

Mock
Infection

R. fascians D188 WT
Infected

R. fascians D188 Δ*lyd*
Infected

Figure S29 Representative images from an excised leaf assay with *N. tabacum* infected with mock solution (100 mM $MgCl_2$), *R. fascians* D188 WT or *R. fascians* D188 Δ*lyd* (n = 15 leaves per condition). Leaves were assessed three weeks after infection.

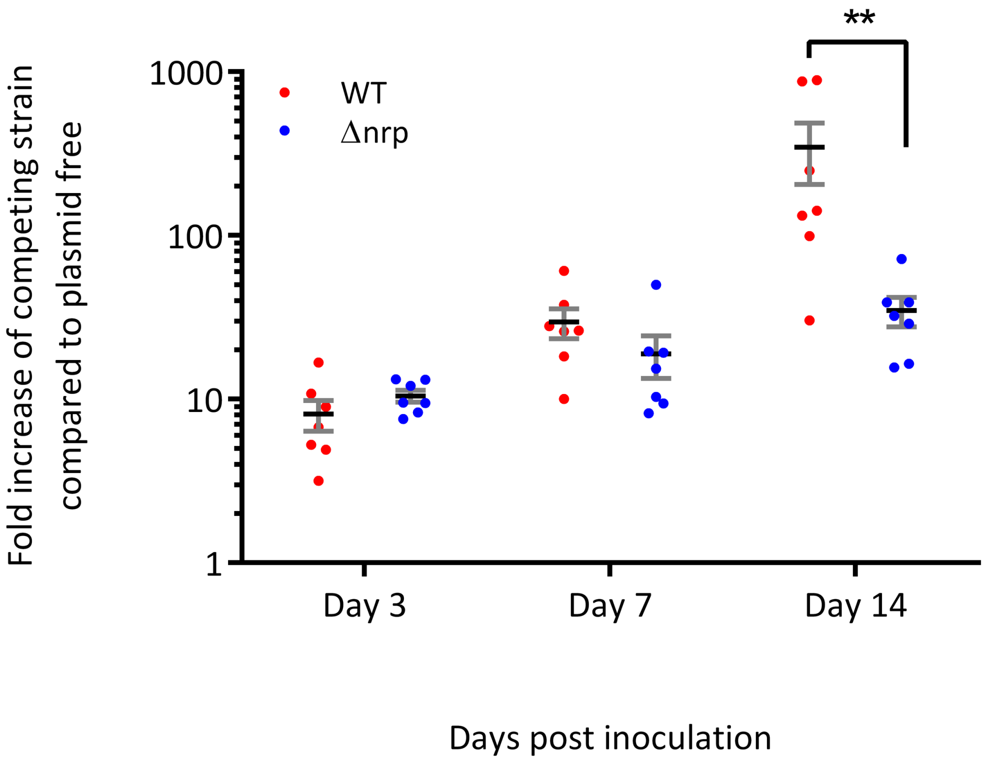

Figure S30 Competition assay of plasmid-free *R. fascians* D188-5 against WT and Δ*lyd* strains across three time points. Percentage of WT (red) and Δ*lyd* (blue) are plotted as a fold-increase compared to the plasmid-free strain. Individual biological replicates are indicated as dots and the mean of these biological replicates is shown as a black cross-bar. Error bars (grey) indicate the standard error of the mean. A Two-way ANOVA showed a significant interaction between strain and time point ($p<0.05$) with a significant effect of both days post inoculation ($p<0.01$) and competing strain ($p<0.05$). Sidak's multiple comparisons showed a significant difference between WT and Δ*lyd* at day 14 post inoculation ($p<0.01$, **).

SUPPLEMENTARY REFERENCES

1. Paysan-Lafosse, T. *et al.* InterPro in 2022. *Nucleic Acids Res.* **51**, D418–D427 (2022).
2. Camacho, C. *et al.* BLAST+: architecture and applications. *BMC Bioinformatics* **10**, 421 (2009).
3. Zallot, R., Oberg, N. & Gerlt, J. A. The EFI Web Resource for Genomic Enzymology Tools: Leveraging Protein, Genome, and Metagenome Databases to Discover Novel Enzymes and Metabolic Pathways. *Biochemistry* **58**, 4169–4182 (2019).
4. Shannon, P. *et al.* Cytoscape: a software environment for integrated models of biomolecular interaction networks. *Genome Res.* **13**, 2498–2504 (2003).
5. Larkin, M. A. *et al.* Clustal W and Clustal X version 2.0. *Bioinformatics* **23**, 2947–2948 (2007).

6. Miller, M. A. *et al.* A RESTful API for Access to Phylogenetic Tools via the CIPRES Science Gateway. *Evol. Bioinform. Online* **11**, 43–48 (2015).

7. Stamatakis, A. RAxML version 8: a tool for phylogenetic analysis and post-analysis of large phylogenies. *Bioinformatics* **30**, 1312–1313 (2014).

8. Letunic, I. & Bork, P. Interactive Tree Of Life (iTOL) v5: an online tool for phylogenetic tree display and annotation. *Nucleic Acids Res.* **49**, W293–W296 (2021).

9. Gilchrist, C. L. M. & Chooi, Y.-H. clinker & clustermap.js: automatic generation of gene cluster comparison figures. *Bioinformatics* **37**, 2473–2475 (2021).

10. Blin, K. *et al.* antiSMASH 6.0: improving cluster detection and comparison capabilities. *Nucleic Acids Res.* **49**, W29–W35 (2021).

11. Prieto, C., García-Estrada, C., Lorenzana, D. & Martín, J. F. NRPSsp: non-ribosomal peptide synthase substrate predictor. *Bioinformatics* **28**, 426–427 (2011).

12. Bachmann, B. O. & Ravel, J. Chapter 8 Methods for In Silico Prediction of Microbial Polyketide and Nonribosomal Peptide Biosynthetic Pathways from DNA Sequence Data. *Methods Enzym.* **458**, 181–217 (2009).

13. Röttig, M. *et al.* NRPSpredictor2--a web server for predicting NRPS adenylation domain specificity. *Nucleic Acids Res.* **39**, W362-7 (2011).

14. Sievers, F. *et al.* Fast, scalable generation of high-quality protein multiple sequence alignments using Clustal Omega. *Mol. Syst. Biol.* **7**, MSB201175 (2011).

15. Robert, X. & Gouet, P. Deciphering key features in protein structures with the new ENDscript server. *Nucleic Acids Research* **42**, W320-4 (2014).

16. Mirdita, M. *et al.* ColabFold: making protein folding accessible to all. *Nat Methods* **19**, 679–682 (2022).

17. Jumper, J. *et al.* Highly accurate protein structure prediction with AlphaFold. *Nature* **596**, 583–589 (2021).

18. Steinegger, M. & Söding, J. MMseqs2 enables sensitive protein sequence searching for the analysis of massive data sets. *Nat. Biotechnol.* **35**, 1026–1028 (2017).

19. Clements, J. M. *et al.* Antibiotic Activity and Characterization of BB-3497, a Novel Peptide Deformylase Inhibitor. *Antimicrob. Agents Chemother.* **45**, 563–570 (2001).

20. Pettersen, E. F. *et al.* UCSF ChimeraX: Structure visualization for researchers, educators, and developers. *Protein Sci.* **30**, 70–82 (2021).

21. Francis, I. *et al.* pFiD188, the linear virulence plasmid of Rhodococcus fascians D188. *Mol. Plant Microbe Interact.* **25**, 637–647 (2012).

22. Nachtigall, J. *et al.* Two new aurachins from Rhodococcus sp. Acta 2259. *J. Antibiot.* **63**, 567–569 (2010).

23. Kurosawa, K. *et al.* Rhodostreptomycins, Antibiotics Biosynthesized Following Horizontal Gene Transfer from Streptomyces padanus to Rhodococcus fascians. *J. Am. Chem. Soc.* **130**, 1126–1127 (2008).

24. Temmerman, W. *et al.* Leafy Gall Formation Is Controlled byfasR, an AraC-Type Regulatory Gene in Rhodococcus fascians. *J. Bacteriol.* **182**, 5832–5840 (2000).

25. Leipoldt, F. *et al.* Warhead biosynthesis and the origin of structural diversity in hydroxamate metalloproteinase inhibitors. *Nat. Commun.* **8**, 1965 (2017).
26. Crone, W. J. K. *et al.* Dissecting Bottromycin Biosynthesis Using Comparative Untargeted Metabolomics. *Angew. Chem. Int. Ed.* **55**, 9639–9643 (2016).
27. Wang, M. *et al.* Sharing and community curation of mass spectrometry data with Global Natural Products Social Molecular Networking. *Nat. Biotechnol.* **34**, 828–837 (2016).
28. Aron, A. T. *et al.* Reproducible molecular networking of untargeted mass spectrometry data using GNPS. *Nat. Protoc.* **15**, 1954–1991 (2020).
29. Winter, G. *et al.* DIALS: implementation and evaluation of a new integration package. *Acta Crystallogr. Sect. D* **74**, 85–97 (2018).
30. Sheldrick, G. M. SHELXT – Integrated space-group and crystal-structure determination. *Acta Crystallogr. Sect. A: Found. Adv.* **71**, 3–8 (2015).
31. Sheldrick, G. M. Crystal structure refinement with SHELXL. *Acta Crystallogr. Sect. C* **71**, 3–8 (2015).
32. Dolomanov, O. V., Bourhis, L. J., Gildea, R. J., Howard, J. A. K. & Puschmann, H. OLEX2: a complete structure solution, refinement and analysis program. *J. Appl. Crystallogr.* **42**, 339–341 (2009).
33. Triccas, J. A., Parish, T., Britton, W. J. & Gicquel, B. An inducible expression system permitting the efficient purification of a recombinant antigen from Mycobacterium smegmatis. *FEMS Microbiol. Lett.* **167**, 151–156 (1998).
34. Ranjan, A., Mercier, E., Bhatt, A. & Wintermeyer, W. Signal recognition particle prevents N-terminal processing of bacterial membrane proteins. *Nat. Commun.* **8**, 15562 (2017).
35. Bögeholz, L. A. K., Mercier, E., Wintermeyer, W. & Rodnina, M. V. Kinetic control of nascent protein biogenesis by peptide deformylase. *Sci. Rep.* **11**, 24457 (2021).
36. Wei, Y. & Pei, D. Continuous Spectrophotometric Assay of Peptide Deformylase. *Anal. Biochem.* **250**, 29–34 (1997).
37. Nguyen, K. T. & Pei, D. High-Throughput Screening of Peptide Deformylase Inhibitors. *Methods Mol. Med.* **142**, 117–130 (2008).
38. Savory, E. A. *et al.* Phytopathogenic Rhodococcus Have Diverse Plasmids With Few Conserved Virulence Functions. *Front. Microbiol.* **11**, 1022 (2020).
39. Pertry, I. *et al.* Rhodococcus fascians Impacts Plant Development Through the Dynamic Fas-Mediated Production of a Cytokinin Mix. *Mol. Plant-Microbe Interact.* **23**, 1164–1174 (2010).
40. Vereecke, D. *et al.* Chromosomal locus that affects pathogenicity of Rhodococcus fascians. *J. Bacteriol.* **184**, 1112–1120 (2002).
41. Snapper, S. B., Melton, R. E., Mustafa, S., Kieser, T. & Jr, W. R. J. Isolation and characterization of efficient plasmid transformation mutants of Mycobacterium smegmatis. *Mol. Microbiol.* **4**, 1911–1919 (1990).
42. Desomer, J., Dhaese, P. & Montagu, M. V. Conjugative transfer of cadmium resistance plasmids in Rhodococcus fascians strains. *J. Bacteriol.* **170**, 2401–2405 (1988).
43. Hwang, S. *et al.* Structural Revision of Lydiamycin A by Reinvestigation of the Stereochemistry. *Org. Lett.* **22**, 3855–3859 (2020).

44. Huang, X. *et al.* Lydiamycins A-D: cyclodepsipetides with antimycobacterial properties. *Angew. Chem. Int. Ed.* **45**, 3067–3072 (2006).

45. Libis, V. *et al.* Multiplexed mobilization and expression of biosynthetic gene clusters. *Nat. Commun.* **13**, 5256 (2022).

46. Yabuuchi, T. & Kusumi, T. Phenylglycine Methyl Ester, a Useful Tool for Absolute Configuration Determination of Various Chiral Carboxylic Acids. *J. Org. Chem.* **65**, 397–404 (2000).

47. Guilloteau, J.-P. *et al.* The crystal structures of four peptide deformylases bound to the antibiotic actinonin reveal two distinct types: a platform for the structure-based design of antibacterial agents. *J. Mol. Biol.* **320**, 951–962 (2002).

48. Margolis, P. *et al.* Resistance of Streptococcus pneumoniae to Deformylase Inhibitors Is Due to Mutations in defB. *Antimicrob. Agents Chemother.* **45**, 2432–2435 (2001).

49. Kosowska-Shick, K. *et al.* Multistep Resistance Selection and Postantibiotic-Effect Studies of the Antipneumococcal Activity of LBM415 Compared to Other Agents. *Antimicrob. Agents Chemother.* **51**, 770–773 (2006).
